# Strength and Conditioning Society (SCS) 7th Annual Meeting, Murcia, Spain, 2024

**DOI:** 10.3390/sports13050137

**Published:** 2025-04-29

**Authors:** Pedro E. Alcaraz, Konstantinos Spyrou, Anthony J. Blazevich, Tomás T. Freitas, Elena Marín-Cascales, Aarón Manzanares Serrano

**Affiliations:** 1 UCAM Research Center for High Performance Sport, UCAM Universidad Católica de Murcia, 30107 Murcia, Spain; palcaraz@ucam.edu (P.E.A.); tfreitas@ucam.edu (T.T.F.); emarin@ucam.edu (E.M.-C.); 2 Facultad de Deporte, UCAM Universidad Católica de Murcia, 30107 Murcia, Spain; amanzanares@ucam.edu; 3 Strength & Conditioning Society, 30008 Murcia, Spain; a.blazevich@ecu.edu.au; 4 Centre for Exercise and Sports Science Research, School of Medical and Health Sciences, Edith Cowan University, Joondalup 6027, Australia; 5 NAR-Nucleus of High Performance in Sport, São Paulo 04753-060, Brazil

**Keywords:** congress, performance, exercise, health, training, injury

## Abstract

On behalf of the Strength and Conditioning Society (SCS), we are pleased to present the abstracts submitted for the SCS 7th Annual Meeting. The event was held at the Universidad Católica de Murcia (UCAM) headquarters in Murcia, Spain, on 9–11 October 2024, and comprised several invited sessions by international and national speakers on a variety of topics related to biochemistry and exercise physiology, strength and conditioning practices and their application to health, injury prevention, and sports performance. These included strength training in high-performance sports, sport science and training–competition load management in elite environments, biochemistry and exercise physiology and prescription, nutrition and biomechanics, among others. The conference also included practical workshops by renowned academics and practitioners on eccentric training, change of direction ability, and strength and power training in professional team sports, combat sports, and ergospirometry and exercise prescription in specific populations. Finally, the event disseminated up-to-date strength and conditioning research by providing practitioners and researchers with the opportunity to present their most recent findings. All abstracts presented at the SCS 7th Annual Meeting can be found in this Conference Report.

## 1. Introduction

The Strength and Conditioning Society (SCS) is pleased to introduce the abstracts presented at the SCS 7th Annual Meeting. In accordance with the SCS’s vision and mission of disseminating high-quality evidence of the performance and health benefits of strength and conditioning practices worldwide, the 2024 Conference took place in Murcia, Spain, on 9–11 October. The SCS 7th Annual Meeting, held at the Universidad Católica de Murcia (UCAM) headquarters, brought together more than 400 attendees with different expertise (e.g., sports science, sports physiotherapy, sports nutrition, exercise physiology and biochemistry, and sports medicine, among others), which provided ample opportunities to exchange and discuss the latest evidence on strength and conditioning practices from multiple perspectives. In a stimulating social and professional environment, practitioners and academics from different countries had the opportunity to attend several thought-provoking sessions provided by international and national speakers on a variety of applied topics related to strength training in high-performance sports and children and the elderly, sport science and load management in elite environments or injury prevention and optimizing the rehabilitation process in different injuries. As in previous years, the conference also offered multiple practical workshops by renowned academics and practitioners on strength and conditioning and nutrition in Ultimate Fighting Championship (UFC), cancer and strength training, strength and power training in team sports, or recovery strategies in high-performance sports. The event also fostered the dissemination of up-to-date strength and conditioning research by providing practitioners and researchers with the opportunity to present and discuss their latest findings, which can be found in the abstracts that compose this Conference Report. Finally, the SCS recognized professional and academic excellence in the field of strength and conditioning and presented the “Female Strength and Conditioning Coach of the Year Award”, the “Male Strength and Conditioning Coach of the Year Award”, the “Emerging Strength and Conditioning Coach of the Year Award” and the “Strength and Conditioning Coach Career Achievement Award” to outstanding coaches, and the “Young Investigator Award” (YIA) and the “Applied Science Award” (ASA) to remarkable researchers. It should be noted that the selection process for our award winners was entirely blended, with a committee for both professional and academic awards. All the abstracts were reviewed by the Scientific Committee. For example, for both academic awards (YIA and ASA), we selected 18 semifinalists for each award, who presented their oral presentations under the supervision of a panel of three experts from the Scientific Committee. From among the 18 semifinalists, three finalists were chosen in each area, and they presented their work again on the final day of the event. The YIA finalists were the authors of the abstracts titled “Concurrent training in older people after valve replacement surgery. A randomized controlled study”, “Effects of a multicomponent training and a detraining period on cognitive and functional performance of older adults at risk of frailty”, and “Effects of different velocity loss thresholds with and without blood flow restriction during squat exercise on strength gains and jump performance.” The ASA finalists were the authors of the abstracts titled “Stair-climbing versus machine-based resistance exercise to improve muscle power among older adults”; “Effects of core centering training on balance, trunk control, and athletic performance in adolescent female volleyball players”; and “Eccentric phase velocity determines the load-velocity relationship in the squat jump and bench press throw exercise: a preliminary study”. The aforementioned committee selected the winners of the awards, which went to Anna Moradell Fernández from Spain for the YIA and to Evelien Van Roie from Belgium for the ASA.

## 2. Conference Abstracts

### 2.1. Comparison of Vastus Lateralis Hypertrophy in Normobaric Hypoxia: Effect of Environmental Conditions in Rooms vs. Tents

Abril Jiménez J. ^1,^*, Almeida F. ^2,^*, Pérez-Regalado S. ^2^, Benavente C. ^2^, Padial P. ^2^, Timón R. ^1^ and Feriche B. ^2^

^1^  Faculty of Sport Sciences. University of Extremadura, Cáceres (Spain); jabrilji@alumnos.unex.es
^2^  Department of Physical Education and Sport, Faculty of Sport Sciences, University of Granada, Granada (Spain)

* Correspondence: filipaalmeida@ugr.es

**Abstract:** Skeletal muscles adapt structurally and functionally in response to different stimuli, such as sports training or a combination of resistance training (RT) and systemic hypoxic conditions (1). A hypoxic environment may induce some beneficial responses in hypertrophic adaptations (2). Metabolite accumulation, cellular swelling, and acute hormonal response, combined with an increase in the production of local myokines and reactive oxygen species (ROS) in response to metabolic stress, are considered important regulators of muscle adaptive responses (3). Twenty-four individuals (22.16 ± 2.94 years old; 176.79 ± 7.47 cm; 76.32 ± 11.00 kg) participated in an 8-week hypertrophy RT program (three sessions/week) under systemic moderate normobaric hypoxia (NH; FiO_2_ = 15.9%) in a tent (NHTent; 32 m^3^; air flux rate of 50 L/min/person) or a room (NHRoom; 240 m^3^; air flux rate of 900 L/min/person). Environmental CO_2_ was monitored during the RT sessions. Muscle thickness in the vastus lateralis (VL) was measured before and after the RT program. The CO_2_ levels at the end of the sessions were significantly higher in the tent (6374 ± 1766 ppm) than in the room (2158 ± 629 ppm) (*p* < 0.001, ES = 3.04 [1.97, 4.09]). On the contrary, the pre and post differences in the VL was significantly higher in the room (∆ = 0.21 ± 0.18 mm, *p* < 0.001, ES = 1.57 [0.79, 2.30]) than in the tent (∆ = 0.08 ± 0.14 mm, *p* = 0.11, ES = 0.47 [−0.01, 0.95]) (*p* 0.062, ES = −0.807 [−1.66, −0.07 mm]). The fact that only the NHRoom showed significant increases in VL thickness after the RT program may be explained by the impact of the exposure to higher CO2 levels in the tent, which reduces the alveolar pressure of O_2_, increasing tissue hypoxia for the same FiO_2_, on the muscle activation myogenic process. Therefore, the harsher conditions in the tent seem to increase physiological stress, similar to conditions at high altitudes (4000–5000 m) compared with moderate altitudes, in which the structural adaptations are usually impaired due to the need to preserve skeletal muscle regeneration (4). Therefore, the device used to simulate hypoxia must be considered when developing this type of training.

**Keywords:** hypoxia; normobaric hypoxia; hypertrophy; strength; vastus lateralis

**Funding:** This research was funded by the Spanish Ministry of Science, Innovation and Universities under grant number PGC2018-97388-B-I00 and by the Andalusian FEDER Operational Program under grant numbers B-CTS-374-UGR20 and C-SEJ 015-UGR23.

**References**
  1. Kon M, Ohiwa N, Honda A, Matsubayashi T, Ikeda T, Akimoto T, Suzuki Y, Hirano Y, Russell AP. Effects of systemic hypoxia on human muscular adaptations to resistance exercise training. Physiol Rep. 2014, 6, 2(6).
  2. Faiss R, Girard O, Millet GP. Advancing hypoxic training in team sports: from intermittent hypoxic training to repeated sprint raining in hypoxia. Br J Sports Med. 2013, 47, 45–50.
  3. Schoenfeld BJ. Potential mechanisms for a role of metabolic stress in hypertrophic adaptations to resistance training. Sports Med. 2013, 43(3), 179–94.
  4. Mizuno M, Savard GK, Areskog NH, Lundby C, Saltin B. Skeletal muscle adaptations to prolonged exposure to extreme altitude: a role of physical activity? High Alt Med Biol. 2008, 9(4), 311–7.

### 2.2. Lab on a Strap: The Promising Future of Continuously Measuring Lactate in Sweat

Ramiro Alonso-Salinas ^1^, Francisco Javier Martínez-Noguera ^2^, Cristian Marín-Pagán ^2^, Xing Xuan ^3^, Daniel Rojas ^3^, Maarten Gijssel ^1^, Pedro E. Alcaraz ^2^, María Cuartero ^3,4^ and Gastón A. Crespo ^3,4,^*

^1^  IDRO B.V., Bd de Waterloo 65, 1000 Bruxelles, Belgium.; ramiro@idro.world; maarten@idro.world
^2^  UCAM Research Center for High Performance Sport, Murcia 30107, Spain.; fjmartinez3@ucam.edu, cmarin@ucam.edu, palcaraz@ucam.edu
^3^  UCAM-SENS, Universidad Católica San Antonio de Murcia, UCAM HiTech, 30107 Murcia, Spain.; xxuan@ucam.edu, jdrojas@ucam.edu
^4^  Department of Chemistry, KTH Royal Institute of Technology, SE-114 28 Stockholm, Sweden.; gacp@kth.se, mariacb@kth.se

* Correspondence: gacp@kth.se

**Abstract:** Lactate is one of the most important markers in the sports field. This metabolite is closely related to energy production in our muscles under anaerobic conditions, particularly during high-intensity exercise. Over the past few decades, its relationship with exercise intensity and the body’s ability to recover from physical activity has been well established [1,2]. Despite its significance, the only way to currently measure lactate is through blood tests, which require invasive skin pricks. Additionally, this method provides only snapshot measurements, potentially missing relevant data. However, there is another fluid in which this analyte can be monitored: sweat. Although the relationship between blood lactate and sweat lactate is not completely clear, it is a promising field of research. The UCAM-SENS research group, in collaboration with the company IDRO B.V., has developed the first sealable amperometric sensor for continuous lactate measurement in sweat [3,4]. This device, similar to a conventional heart rate monitor, contains a cartridge and the sensor inside. A microfluidic channel guides the user’s sweat to the sensor, which emits an electrical signal that can be translated into a continuous lactate concentration. This technology is minimally invasive, causing no more discomfort to the user than a Polar-type heart rate monitor. In collaboration with CIARD, we have tested this technology on both elite and amateur athletes, following various protocols. In these studies, particularly using the lactate interval protocol, we found correlations (Pearson coefficient values over 0.85) between sweat lactate and different parameters such as blood lactate, ventilation equivalent (VE), work (W), oxygen consumption (VO2), and carbon dioxide production (VCO2). These results are promising, suggesting that sweat lactate monitoring could offer a continuous and dynamic view of an athlete’s physiology during exercise. Continuous lactate monitoring in sweat would not only allow real-time assessment of the athlete’s physical state but also facilitate the development of personalized training programs. Coaches and athletes could adjust the intensity and duration of exercise based on precise, real-time data, thereby improving performance and minimizing the risk of injuries. Furthermore, this technology could be used to assess post-exercise recovery and provide crucial information on when it is safe and effective to resume intense physical activity, thus unlocking peak performance through sweat monitoring.

**Keywords:** sweat lactate; sport performance; sports physiology

**Funding:** This research was funded by IDRO B.V.

**References**
  1. Nichols, J.F.; Phares, S.L.; Buono, M.J. Relationship between Blood Lactate Response to Exercise and Endurance Performance in Competitive Female Master Cyclists. International journal of sports medicine 1997, 18, 458–463, doi:10.1055/s-2007-972664.
  2. Jamnick, N.A.; Pettitt, R.W.; Granata, C.; Pyne, D.B.; Bishop, D.J. An Examination and Critique of Current Methods to Determine Exercise Intensity. Sports Medicine 2020, 50, 1729–1756, doi:10.1007/s40279-020-01322-8.
  3. Xuan, X.; Chen, C.; Pérez-Ràfols, C.; Swarén, M.; Wedholm, L.; Cuartero, M.; Crespo, G.A. A Wearable Biosensor for Sweat Lactate as a Proxy for Sport Performance Monitoring. Analysis & Sensing 2023, 3, e202200047, doi:https://doi.org/10.1002/anse.202200047.
  4. Van Hoovels, K.; Xuan, X.; Cuartero, M.; Gijssel, M.; Swarén, M.; Crespo, G.A. Can Wearable Sweat Lactate Sensors Contribute to Sports Physiology? ACS Sensors 2021, 6, 3496–3508, doi:10.1021/acssensors.1c01403.

### 2.3. Psychological Factors Associated with Healthy Sports Practice

Francesca Ancarani ^1^, Óscar Gavín-Chocano ^2^, David Molero ^2,^*, and Germán Vicente-Rodríguez ^1,^*

^1^  EXER-GENUD (Growth, Exercise, Nutrition and Development) Research Group, University of Zaragoza, Spain; fancarani@gmail.com (F.A.); gervicen@unizar.es (G.V.-R.)
^2^  Department of Education, University of Jaén, Spain; ogavin@ujaen.es (O.G.-C.); dmolero@ujaen.es (D.M.)

* Correspondence: dmolero@ujaen.es; Tel.: +34 953 213436. Department of Education. University of Jaén. Campus las Lagunillas s/n, 23071 Jaén, Spain.
* Correspondence: gervicen@unizar.es EXER-GENUD (Growth, Exercise, Nutrition and Development) Research Group, Universidad de Zaragoza, 50009 Zaragoza, Spain

**Abstract:** This study aimed to determine the psychological characteristics associated with the performance of amateur athletes, exploring their relationship with key variables such as resilience, harmonious and obsessive passion, and perception of discomfort. The main objective was to establish the relationship between psychological characteristics and each of the variables used, i.e., resilience (personal competence and acceptance of self and life), and to consider whether these are related to passion (harmonious and obsessive) through structural equation analysis. The sample consisted of 110 people: 87 adults (22 females, mean age 40.7 ± 9.1 years, and 65 males, mean age 42.1 ± 11.9 years) and 23 adolescents (16 females, mean age 14.8 ± 1.3 years, and seven males, mean age 15.3 ± 2.1 years). Psychological characteristics were assessed using the Psychological Characteristics Related to Performance (CPRP) questionnaire, passion using the Passion Scale instrument, and the Resilience Scale -RS-14 questionnaire. The analyses performed were based on structural equation modeling (PLS-SEM) analysis. The results showed adequate coefficients of determination (R^2^ index) and Stone–Geisser predictive significance (Q^2^) for the resilience factors personal competence (R^2^ = 0.517; Q^2^ = 0.218) and acceptance of self and life (R^2^ = 0.415; Q^2^ = 0.231), and for the passion dimensions harmonious passion (R^2^ = 0.357; Q^2^ = 0.168) and obsessive passion (R^2^ = 0.085; Q^2^ = 0.034). In conclusion, a close relationship is demonstrated between psychological characteristics related to sport performance and the variables resilience and passion (both harmonious and obsessive). In particular, it is confirmed that mental skills and stress management are linked to dimensions of resilience, and these dimensions influence both types of passion. Furthermore, team cohesion and personal competence also play a crucial role in the development of passion, highlighting the importance of these factors in the assessment of sport performance and influencing the well-being of both professional and amateur athletes in their personal and sports development.

**Keywords:** health; passion; psychological characteristics; resilience; sport psychology

**Funding:** This research received no external funding.

### 2.4. Sex Differences in Hip and Groin Injury Prevalence: A Systematic Review and Meta-Analysis

Toni Bailén-García ^1,2^, Marcos Quintana-Cepedal ^1,2^, Omar de la Calle ^2^, María Medina-Sánchez ^2,3^, Irene Crespo ^1^, Hugo Olmedillas ^1,2,^*

^1^  Department of Functional Biology, University of Oviedo, 33006 Oviedo, Spain; tonibailenito@gmail.com (T.B.-G.); marcosquintana99@gmail.com (M.Q.-C.); crespoirene@uniovi.es (I.C.); olmedillashugo@uniovi.es (H.O.)
^2^  Asturian Research Group in Performance, Readaptation, Training, and Health (AstuRES), Oviedo, Spain: omarfdzdelacalle@gmail.com (O.C.); mmedina@uniovi.es (M.M.-S.)
^3^  Department of Surgery, University of Oviedo, 33006 Oviedo, Spain

* Correspondence: olmedillashugo@uniovi.es

**Abstract:** Hip and groin injuries (HGIs) are very common in elite sports that involve changes in direction, kicks, jumps, and sprints. While male sex has traditionally been viewed as a risk factor, recent studies have presented contradictory findings. The aim of this meta-analysis is to compare the prevalence of HGIs among male and female athletes. A systematic search for related studies published from database inception to February 2024 was conducted in the following databases: Cochrane, PubMed/MEDLINE, Web of Science, Embase, SPORT Discus, PEDro, and Scopus. A total of eight clinical trials, cohort studies, and case–control studies were included. Data recorded from each study included authors, year of publication, study design, number of participants, sport played, age, number of injured subjects, injury prevalence, and type of injury (time-loss (TL) and/or non-time-loss (NTL)). Pooled odds ratios (ORs) with 95% confidence intervals (CIs) were calculated using random-effects meta-analysis. The risk of bias (RoB) was evaluated with the Quality in Prognosis Studies (QUIPS) tool, and the certainty of the evidence was evaluated using the Grading of Recommendations, Assessment, Development, and Evaluations (GRADE) approach. The pooled results of 2358 athletes (1517 males and 841 females) showed no differences in HGIs between sexes, with an OR of 1.38 (95% CI: 0.92–2.06; PI: 0.39–4.90; *p* = 0.12). No differences were observed for TL (OR = 1.55) and NTL (OR = 0.97) injuries. However, male football players showed higher odds of suffering groin problems (OR = 2.25). This systematic review and meta-analysis reveals a similar prevalence of HGIs in males and females. The results highlight the importance of establishing screening measures for all athletes.

**Keywords:** inguinal pain; soccer; prevention; female; epidemiology

**Funding:** This research received no external funding.

**References**
  1. Orchard JW. Men at higher risk of groin injuries in elite team sports: A systematic review. Br J Sports Med. 2015;49:798–802.
  2. Whittaker JL, Small C, Maffey L, Emery CA. Risk factors for groin injury in sport: an updated systematic review. Br J Sports Med. 2015;49:803–809.
  3. Quintana-Cepedal M, De La Calle O, Medina-Sánchez M, Crespo I, Del Valle M, Olmedillas H. Characterising groin pain in rink hockey: Function and five-second squeeze in Spanish players. Phys Ther Sport. 2022;58:100–105.

### 2.5. Classification of Basketball Players Based on Multidirectional Speed Performance: Thresholds of Change of Direction Deficit

Francisco J. Barrera-Domínguez ^1,^*, Paul A. Jones ^2^, Bartolomé J. Almagro ^1^ and Jorge Molina-López ^1^

^1^  Faculty of Education, Psychology and Sport Sciences, COIDESO, University of Huelva, Huelva, Spain
^2^  Directorate of Sport, Exercise and Physiotherapy, University of Salford, Greater Manchester, United Kingdom

* Correspondence author: francisco.barrera@ddi.uhu.es

**Abstract:** High-intensity actions such as sprints and changes of direction (COD) significantly influence the outcome of a basketball game (1). The performance of these actions is often assessed by measuring the execution time of specific tests. However, this method might not provide relevant data about the individual needs of each athlete. In this context, COD deficit (CODD) offers valuable insights into a player’s trade-off between multidirectional and linear speed (2). However, established reference values for CODD are lacking. This study aims to determine CODD thresholds for various COD angles in basketball players and to analyze the relationships between CODD and execution time in speed tests. One hundred and thirty trained basketball players (46% female; age: 23.7 ± 5.29 years; height: 189.1 ± 11.1 cm; body mass: 84.3 ± 15.7 kg) undertook 10-metre linear and COD speed tests at 45°, 90°, and 180°. A k-means cluster analysis was conducted to standardize CODD thresholds, and a one-way analysis of variance was conducted to identify differences between clusters. The results showed a significant relationship between CODD and COD execution time for each specific cutting angle (r ≥ 0.43; *p* < 0.01), revealing angle-specific CODD thresholds ranging from 3% to 8%, 17% to 25%, and 43% to 51% for 45°, 90° and 180° cutting angles, respectively. Furthermore, significant differences (*p* < 0.05) were observed in execution times between clusters for all cutting angles. Players with a high CODD (classified as linear speed dominant) demonstrated higher performance in linear speed, while those with a low CODD at each cutting angle (classified as multidirectional speed dominant) showed significantly higher performance in COD. Balanced players, who achieved the best relationship between both actions, exhibited the best overall performance. Therefore, strength and conditioning coaches are encouraged to assess CODD as a highly valid variable for evaluating COD performance and to use current CODD thresholds to tailor training programs according to each athlete’s needs. These findings may empower coaches to effectively identify their athletes’ strengths and weaknesses in COD actions based on the specific cutting angles, thereby optimizing speed training in basketball players.

**Keywords:** agility; sprint; assessment; team sport; performance

**Funding:** This research was funded by the Formación del Profesorado Universitario Programme (Ministerio de Ciencias, Innovación y Universidades, Government of Spain), grant number FPU22/01057.

**References**
  1. Salazar, H.; Castellano, J.; Svilar, L. Differences in external load variables between playing positions in elite basketball match play. J Hum Kinet 2020, 75(1), 257–266.
  2. Freitas, T. T.; Pereira, L. A.; Alcaraz, P. E.; Azevedo, P. H. S. M.; Bishop, C.; Loturco, I. Percentage-based change of direction deficit: A new approach to standardize time- and velocity-derived calculations. J Strength Cond Res 2021, 36(12), 3521–3526.

### 2.6. Effects of Different Training Models on Neuromuscular Parameters and Functional Capacity of Older Individuals

Eduarda Blanco-Rambo ^1^, Nadyne Rubin ^2^, Marcelo Bandeira-Guimarães ^1^, Caroline Rosa Muraro ^4^, Débora Marques ^5^, Greyse Dornelles Mello ^6^, Andressa Fergutz ^7^, Caroline Pietta-Dias ^1^, Eduardo Lusa Cadore ^1,^*

^1^  Universidade Federal do Rio Grande do Sul, eduardarambo@grnail.com, adynerubin@yahoo.com.br, bgbandeirazz@gmail.com, caroline.rmuraro@gmai1.com, dedy.marques@hotmai1.com, greysedorne1les@gmail.com, andressaufrgs@rgmai1.com, carolpieta@yahoo.com.br

* Correspondence: edcadoreú@yahoo.com.br; Tel.: +55-(51)-99119-3651

**Abstract:** Functional capacity maintenance is an important outcome for a good quality of life throughout the aging process. It is affected by several factors, such as muscle strength and power, as well as cardiorespiratory fitness. Different exercise interventions have been applied to improve these parameters. This study aimed to compare the effects of combined strength and endurance training (SEG) and combined strength training with dance classes (SDG) on muscle strength, power output, and functional capacity of older individuals. Maximal strength was assessed using the one maximal repetition test (IRM) on knee extension exercise. Mean (Pmean) and maximal (Pmax) power outputs were evaluated through knee extension exercise at 30% and 70% of IRM using a linear displacement sensor. Functional capacity was assessed through the sit-to-stand test (STS) and the 6-min walking test (6WT). Training was performed twice weekly over 12 weeks, with progressive intensity and volume. Both interventions were composed of strength training followed by walking (SEG) or dance classes (SDG), with equivalent session times. Generalized Estimating Equations were used to verify the main effects of time, group, and time vs. group interaction, and pairwise comparisons were performed using Minimum Significant Difference post-hoc. Significance was accepted when *p* < 0.05. The results are presented as mean change + standard deviation. Forty-eight participants (81% women; mean age: 68.7 years) were randomized into two groups, and 28 completed the intervention (SEG n = 15; SDG n = 13). After intervention, there was a significant time effect for IRM, Pmean at 30% and 70%, Pmax at 30% and 70%, as well as STS and 6WT (*p* values ranging from <0.02 to <0.00) in both groups, without time vs. group interactions. Significant increases were observed for SEG in the IRM (fi% = 32.9 + 13.75), Pmean 30% (fi% = 44.7 + 25.2) and 70% (fi% = 39.2 + 49.5), Pmax 30% (fi% = 30.7 + 58.3) and 70% (fi% = 28.5 + 99.2), STS (fi% = 20.7 + 3.2) and 6WT (fi% = 3.5 + 86.5), and for SDG in the 1RM (fi% = 24.3 + 23), Pmean 30% (fi% = 34.8 + 45) and 70% (fi% = 36.2 + 75.3), Pmax 30% (fi% = 35.9 + 98.2) and 70% (fi% −38.2 + 128.9), STS (fi% −33.6 + 2.3) and 6WT (fi% = 3.8 + 48). Thus, both interventions were capable of improving neuromuscular variables and functional capacity. Dance classes seem to be an alternative to aerobic exercise in improving physical fitness in older individuals.

**Keywords:** muscle strength; power output; aging; resistance training, endurance training, combined training; dance classes

**Funding:** This research was funded by the National Council for Scientific and Technological Development (CNPq) and by the Coordination for the Improvement of Higher Education Personnel (CAPES).

**References**
  1. Cadore, EL; Pinto, RS; Pinto, SS; et al. Effects of Strength, Endurance, and Concurrent Training on Aerobic Power and Dynamic Neuromuscular Economy in Elderly Men. *J Strength Cond Res*. **2011**, 25, 758–766.
  2. Blanco-Rambo, E; Izquierdo, M; Cadore, EL. Dance as an Intervention to Improve Physical and Cognitive Functioning Older Adults. *Nutr Health Agíng.* **2023**, 27, 75–76.
  3. Blanco-Rambo, E; Rubin, N; Bandeira-Guimarães, M; et al. Efeitos do treinamento combinado tradicional e do treinamento multicomponente composto por treinamento de força e aulas de dança na capacidade funcional e cognitiva de idosos: protocolo de estudo. Ter. *Bras. Ativ.* fís. Sdude. **2024**, 29, 1–14.

### 2.7. Association Between Nutritional Status and Upper and Lower Extremity Muscle Strength in Children and Adolescents with Cystic Fibrosis

Ángela Blanco-Velasco ^1^, Lisset Pantoja-Arévalo ^1^, Thomas Yvert ^1^, Elena Tundidor Canfran ^1^, Tamara Iturriaga Ramirez ^2^, Olga Barceló Guido ^2^, Verónica Sanz Santiago ^3^, and Margarita Perez-Ruiz ^1,^*

^1^  ImFINE Research Group, Department of Health and Human Performance, Universidad Politécnica de Ma drid, 28040 Madrid, Spain
^2^  Departamento de Ciencias del Deportes, Faculty of Sport Sciences, Universidad Europea de Madrid, 28670 Madrid, Spain
^3^  Pulmonology Unit, Hospital Universitario Infantil Niño Jesús de Madrid, Madrid, Spain

* Correspondence: Departamento de Salud y Rendimiento Humano, Facultad de Ciencias de la Actividad Física y del deporte, Universidad Politécnica de Madrid, C/Martín Fierro, 7, Ciudad Universitaria, 28040 Madrid, Spain. E-mail address: margarita.perez@upm.es (M. Pérez-Ruiz)

**Abstract:** Nutritional status is often linked to good respiratory health and survival in people with cystic fibrosis (CF). Almost 85% of current CF patients have started a new course of treatment with cystic fibrosis transmembrane conductance regulator (CFTR) modulators, which improve lung function, without knowing the impact that this treatment may have on muscle function and nutritional status. The objective of this work was to analyze upper and lower extremity strength in children and adolescents with CF who were treated with CFTR modulating drugs to evaluate the associations between extremity strength, lung function, and body composition. The research was an observational study of Spanish children and adolescents between 6 and 18 years of age who had CF. Demographic and lung function data were collected using a spirometer (Jaeger^®^ Vyntus PNEUMO, Mettawa, IL, USA), anthropometric values were measured using a mechanical scale (Asimed^®^ Barys Plus C, Spain), height was measured using a telescopic measuring rod, and body composition was measured using a bioimpedance (Bodystat 1500 MMD, London, UK). Muscle strength was measured in the upper limbs using hand-grip strength (Jamar^®^ Smart-dynamometer, Green Bay, WI, USA), and in the lower extremities, muscle strength was measured using knee extensors and flexors (MicroFET 2-dynamometer, Hoggan, Salt Lake City, UT, USA). Twenty-four Spanish children and adolescents with CF were included in this study (62.5% male, mean age 12.00 ± 2.74 years, FEV1 z-score 0.11 ± 1.19, BMI z-score −0.08 ± 1.34, fat mass percentage 23.83 ± 9.63, and lean mass percentage 76.17 ± 9.63). Hand-grip strength was 17.45 ± 7.43 kg (percentile 19.71 ± 14.20), leg extension strength was 24.84 ± 12.39 kg (0.59 ± 0.19 relative to weight), and leg flexion strength was 13.18 ± 5.29 kg (0.32 ± 0.08 relative to weight). Positive associations were observed between grip strength and muscle mass percentage (*p* = 0.045) and lower extremity strength (*p* < 0.001), while a negative association was observed between grip strength and fat mass percentage (*p* = 0.045). Additionally, this may have an advantage in terms of respiratory status, as determined by absolute FEV1 values. In conclusion, the grip strength of children and adolescents with CF appears to be weaker than that of healthy children their age. In addition, there seems to be a positive correlation between grip strength and lower extremity muscle strength, as measured using knee extensors and flexors. Furthermore, these scores may be positively associated with nutritional status, which may be beneficial for their lung function.

**Keywords:** muscle strength; body composition; CFTR modulators; lung function; nutritional assessment; handgrip strength

**Funding:** This research was funded by INSTITUTO SANITARIO CARLOS III, grant number ISCIIIPI 23/00299.

**References**
  1. Corey, M.; McLaughlin, F. J.; Williams, M.; Levison, H. A comparison of survival, growth, and pulmonary function in patients with cystic fibrosis in Boston and Toronto. J Clin Epidemiol 1988, 41, 583–591.
  2. Stallings, V.A.; Stark, L.J.; Robinson, K.A.; Feranchak, A.P.; Quinton, H.; Clinical Practice Guidelines on Growth and Nutrition Subcommittee; Ad Hoc Working Groupet. Evidence-based practice recommendations for nutrition-related management of chil dren and adults with cystic fibrosis and pancreatic insufficiency: results of a systematic review. J Am Diet Assoc 2008, 108, 832–839.
  3. Kapouni, N.; Moustaki, M.; Douros, K.; Loukou, I. Efficacy and Safety of Elexacaftor-Tezacaftor-Ivacaftor in the Treatment of Cystic Fibrosis: A Systematic Review. Children (Basel) 2023, 10, 554.

### 2.8. Effectiveness of Physical Exercise and Dietary Intervention in Patients with NAFLD and Metabolic Syndrome

Pere A. Borras ^1,2,^*

^1^  Physical Activity and Sport Sciences Research Group. University of the Balearic Islands; pa-borras@uib.es
^2^  CIBEROBN; pa-borras@uib.es

* Correspondence: pa-borras@uib.es; Tel.: +34-606957135

**Abstract:** Non-alcoholic fatty liver disease (NAFLD) is currently the most common cause of liver disease in the Western world, affecting up to 30% of the population. It is characterized by the deposition of fat in hepatocytes, and is a chronic inflammatory liver disease that encompasses a spectrum of pathologies that range from simple accumulation of fat or hepatic steatosis, to final stages of the disease, such as cirrhosis, including non-alcoholic steatohepatitis and fibrosis. Its prevalence increases with age and obesity, and it is associated with the presence of metabolic syndrome, increased cardiovascular mortality, and cancer. Regular physical activity (PA) has been shown to have a positive relationship with PA levels and the risk of developing NAFLD, which has been consistently observed in case–control cohort studies. This association appears to operate in a linear dose–response manner in adults and remains statistically significant after adjusting for conventional NAFLD risk factors, suggesting that PA may be an independent protective factor against NAFLD. According to the World Health Organization (WHO), approximately 60% of the world’s population does not perform enough physical activity, i.e., has a sedentary lifestyle, which is considered one of the main risk factors for mortality. The study sample included 155 patients (women and men between 40 and 60 years old) who were overweight/obese, presented with at least three of the main features of metabolic syndrome, and received a diagnosis of non-alcoholic fatty liver disease via ultrasound and corroborated by magnetic resonance imaging. The sample was randomized into three intervention groups: conventional diet with basic PA recommendation, Mediterranean diet with a high frequency of meals and basic PA recommendation, and Mediterranean diet with personalized physical exercise (35 min of moderate intensity interval training sessions 3 times a week for one year). Six visits were scheduled for each patient, with measurement of numerous variables and close monitoring of the participants, focusing on imaging results, anthropometry, psychological well-being, severity of the metabolic syndrome, and indirect liver fibrosis data. The results show that, after one year, all participants showed improvements in most of the parameters regardless of their intervention group, gender, or hormonal status, with those whose elastography results improved most being those with the greatest improvement in fasting triglycerides, HOMA index, and fibrosis scores.

**Keywords:** NAFLD; intervention; diet; physical exercise; SMET severity; fibrosis score; ultrasound; elastography

**Funding:** This research was funded by “la Marato de TV3” (project reference 201630.10), “Instituto de Salud Carlos III, Fondo de Investigación para la Salud” (Project reference PI20/00456), and CIBEROBN (CB12/03/30038).

**References**
  1. ROMERO-GÓMEZ, Manuel; ZELBER-SAGI, Shira; TRENELL, Michael. Treatment of NAFLD with diet, physical activity and exercise. *Journal of hepatology*, 2017, vol. 67, no 4, p. 829–846.
  2. Montemayor, Sofía, et al. Adherence to mediterranean diet and NAFLD in patients with metabolic syndrome: the FLIPAN study. *Nutrients*, 2022, vol. 14, no 15, p. 3186.
  3. Mascaro, Catalina M., et al. Association between stages of hepatic steatosis and physical activity performance in adults with metabolic syndrome: A cross-sectional analysis in FLIPAN Study. *Nutrients*, 2022, vol. 14, no 9, p. 1790.
  4. Abbate, Manuela, et al. Energy expenditure improved risk factors associated with renal function loss in nafld and mets patients. *Nutrients*, 2021, vol. 13, no 2, p. 629.

### 2.9. Promoting Return to Play in Concussed Elite Rugby Players Using Transcutaneous Auricular Vagus Nerve Stimulation

**Luke Canavan Dignam ^1^, Linda H Chung ^2^**

^1^  Atlantic Technological University *
^2^  UCAM Research Center for High Performance Sport, Universidad Católica San Antonio de Murcia

* Correspondence: lukecanavand@gmail.com; Tel.: +353-896165711

**Abstract:** Concussion remains an ever-prevalent and underexplored domain in sports science, with increasing occurrence rates in contact sports such as rugby. Based on the findings of the Concussion in Sport Guidelines, we propose a method of neurorehabilitation for elite rugby players who have suffered a concussion during training or competition, using novel transcutaneous auricular vagus nerve stimulation. The vagus nerve is directly related to the autonomic nervous system and cardiovascular system and acts as a junction box for the two, where their relationship to concussion can be observed via autonomic dysregulation, with heart rate variability. Stimulating this nerve increases neuroplasticity and low-frequency power in the ANS, which in turn, promotes recovery. The objective of this study is to determine whether taVNS rehabilitation is effective in concussion recovery and to compare two different doses (1×/day vs. 2×/day) of taVNS with a sham control group. The study will divide 50 concussed elite rugby players into three cohorts, where group 1 will receive taVNS for 20 min @ 20 Hz/5 ma daily, group 2 will receive the same dose and frequency but will receive it on consecutive days, and group 3 acts as a control, receiving a sham treatment. The results are expected to show that both groups 1 and 2, which received treatment, show a decline in heart rate, an increase in HRV, and low-frequency vagal tone compared with group 3. The proposed study may provide strong conceptual evidence for taVNS to promote recovery in concussed athletes.

**Keywords:** neurorehabilitation; traumatic brain injury; return to play

**Funding:** No funding was received.

**References**
  1. Berger, A., Vespa, S., Dricot, L., Dumoulin, M., Iachim, E., Doguet, P., Vandewalle, G., & Tahry, R. E. (2021). How is the norepinephrine system involved in the antiepileptic effects of vagus nerve stimulation? *Frontiers in Neuroscience*, *15*. https://doi.org/10.3389/fnins.2021.790943.
  2. Obst, M. A., Heldmann, M., Alicart, H., Tittgemeyer, M., & Münte, T. F. (2020). Effect of Short-Term Transcutaneous vagus Nerve stimulation (TVNS) on brain processing of food cues: an electrophysiological study. *Frontiers in Human Neuroscience*, *14*. https://doi.org/10.3389/fnhum.2020.00206.
  3. Silberstein, S. D., Mechtler, L. L., Kudrow, D. B., Calhoun, A. H., McClure, C., Saper, J. R., Liebler, E. J., Engel, E. R., & Tepper, S. J. (2016). Non–Invasive vagus nerve stimulation for the ACUTE treatment of cluster headache: findings from the randomized, Double-Blind, SHAM-Controlled ACT1 study. *Headache*, *56*(8), 1317–1332. https://doi.org/10.1111/head.12896.
  4. Von Rosenberg, W., Chanwimalueang, T., Adjei, T., Jaffer, U., Goverdovsky, V., & Mandic, D. P. (2017b). Resolving Ambiguities in the LF/HF Ratio: LF-HF Scatter Plots for the Categorization of Mental and Physical Stress from HRV. *Frontiers in Physiology*, *8*. https://doi.org/10.3389/fphys.2017.00360.

### 2.10. Exploring Determinants of Agility in Futsal: A Cross-Sectional Study

Ángel Camacho-Carranza ^1,^* and Francisco J. Barrera-Domínguez ^1^

^1^  Universidad de Huelva; angel.camacho@ddi.uhu.es, francisco.barrera@ddi.uhu.es

* Correspondence: angel.camacho@ddi.uhu.es

**Abstract:** Agility in futsal is a key indicator of performance, emphasizing both physical and cognitive skills essential for optimal performance. This study examines the key determinants of agility performance in futsal players through a cross-sectional analysis. The methodology involved 25 male players categorized into two age groups: Youth (U-18) and Seniors. The tests included a 10 m sprint, a planned Y-shaped change of direction (COD) test, and an unplanned Y-shaped reactive agility (RAG) test [1]. A novel methodology for assessing COD performance, the Change of Direction Deficit Percentage (CODD%), was introduced [2]. Results indicated strong positive correlations between RAG, COD, and sprint performance. Significant correlations were observed between RAG and COD (r = 0.58, *p* < 0.01) and between RAG and sprint (r = 0.48, *p* < 0.01) [3]. Additionally, high correlations between sprint and COD variables (r = 0.97, *p* < 0.001) were noted. Further analysis revealed significant negative correlations between the RAG variable and the absolute value of the CODD (CODD-abs) (r = −0.50, *p* < 0.01) and the relative value of the Change of Direction Deficit (CODD-rel) (r = −0.52, *p* < 0.01). Conversely, positive correlations were found between COD and the absolute value of the agility deficit (RAGD-abs) (r = 0.77, *p* < 0.001) and the relative value of the agility deficit (RAGD-rel) (r = 0.62, *p* < 0.01). These findings highlight the complex interplay between agility performance and respective deficits in directional changes and reactive capabilities. The study concluded that both COD speed and sprint speed are primary indicators of agility in futsal, underscoring their importance in player training and development.

**Keywords:** performance; change of direction; change of direction deficit

**Funding:** This research received no external funding.

**References**
  1. Oliver, J.L.; Meyers, R.W. Reliability and generality of measures of acceleration, planned agility, and reactive agility. Int J Sports Physiol Perform 2008, 4, pp. 345–354.
  2. Freitas, T. T.; Pereira, L. A.; Alcaraz, P. E.; Azevedo, P.; Bishop, C.; Loturco, I. Percentage-Based Change of Direction Deficit: A New Approach to Standardize Time- and Velocity-Derived Calculations. J Strength Cond Res 2022, 36, pp. 3521–3526.
  3. Matlák, J.; Fridvalszki, M.; Kóródi, V.; Szamosszegi, G.; Pólyán, E.; Kovács, B.; Kolozs, B.; Langmár, G.; Rácz, L. Relationship between cognitive functions and agility performance in elite young male soccer players. J Strength Cond Res 2024, 38, pp. 116–122.

### 2.11. Force Steadiness and Functional Performance in Frail Older Adults: The Role of Muscle Mass in Cognitive Impairment

Maria-Alejandra Camacho-Villa ^1^, Sonia Rivera-Mejía ^1^, Mathías López Córdoba ^2^, Guillermo Madruga ^2^, Marta Sevilla ^1^ and Eduardo Carballeira ^1,^*

^1^  Performance and Health Group, Department of Physical Education and Sport, University of A Coruña, A Coruña, Spain; m.camacho@udc.es; sonia.riveram@udc.es; marta.sevilla@udc.es; eduardo.carbal-leira@udc.es
^2^  Department of Physical Education and Sport, University of A Coruña, A Coruña, Spain; mathias.lopez.cordoba@udc.es; g.madruga@udc.es

* Correspondence: eduardo.carballeira@udc.es

**Abstract:** Aging is accompanied by a decline in cognitive function, loss of muscle mass, and capacity to produce a steady force during a submaximal voluntary contraction, known as force steadiness (FS) (1,2). These characteristics are essential for physical function, and their impairment has been associated with a decline in functionality in older adults (3). This study aimed to investigate the association between the knee extensor FS, body composition, and performance in functionality tests in institutionalized older adults with cognitive impairment and frailty syndrome (2). Eighteen institutionalized older adults (82 ± 8 years; 1.6 ± 10.4 m; 69 ± 14 kg; BMI: 28 ± 6 kg/m^2^, muscle mass percentage 22 ± 5%) with primarily mild and moderate cognitive impairment (66.7%) as assessed by the Global Deterioration Scale (GDS), performed the Short Physical Performance Battery (SPPB). Additionally, maximum isometric voluntary contraction (MIVC) for knee extensor and steady submaximal isometric contractions at 30% of MIVC in both limbs were assessed using a force sensor. Body composition was measured by bioimpedance (InBody 270) between 8 and 9 am while fasting. Gait speed velocity, Sit-to-Stand (STS) muscle power, muscle mass, and fat mass percentage were recorded to assess the association with FS variables measured by coefficient of variation (CV%), Root Mean Square of the Successive Differences (RMSSD), and Approximate entropy (ApEn). The limb with the lowest CV% was considered for the analysis. Pearson and Spearman correlations were conducted to determine the magnitude of the relationship between variables. Eleven women and seven men participated in the study, with 88% classified as frail according to the SPPB score. The estimated 10-year survival rate, based on the Charlson’s index, was 32 ± 28%. No correlation was found between FS variables and the functionality test (r = −0.05 to 0.33, *p* > 0.05). Interestingly, a strong association was found between CV% and muscle mass (r = −0.7, *p* = 0.001), and a moderate association was found between RMSSD and muscle mass (r = −0.47, *p* = 0.04). These findings suggest that while FS may not be correlated with functionality tests in this population, muscle mass appears to play a crucial role in the steadiness of submaximal force. This highlights the importance of preserving muscle mass and exercise in mitigating declines in FS, a crucial component of neuromuscular control in frail older adults with cognitive impairment. Future research should explore the reliability of FS tests in this population and consider evaluating functionality with tests that reflect neuromuscular control, minimizing the influence of other capacities like strength or power.

**Keywords:** force control; functionality; movement variability

**Funding:** This research received no external funding.

**References**
  1. Pethick J, Taylor MJD, Harridge SDR. Aging and skeletal muscle force control: Current perspectives and future directions. Scand J Med Sci Sports. 2022;32(10):1430–43.
  2. Rosa AD la, Solana E, Corpas R, Bartrés-Faz D, Pallàs M, Vina J, et al. Long-term exercise training improves memory in middle aged men and modulates peripheral levels of BDNF and Cathepsin B. Sci Rep. 2019;9(1):3337.
  3. Pethick J, Piasecki M. Alterations in Muscle Force Control With Aging: Is There a Modulatory Effect of Lifelong Physical Activity? Front Sports Act Living. 2022;4:817770.

### 2.12. Inter-Session Reliability of Load–Velocity Profile and Dynamic Strength Index (DSI) in Chinese Elite Judokas

Eduardo Carballeira ^1,^*, Felipe Sánchez-Llanes ^2^, Shengtao Yang ^3,4^, Maria-Alejandra Camacho-Villa ^1^, Sonia Rivera-Mejía ^1^ and Marta Sevilla-Sanchez ^1^

^1^  Performance and Health Group, Department of Physical Education and Sport, University of A Coruña, A Coruña, Spain; eduardo.carballeira@udc.es; marta.sevilla@udc.es; m.camacho@udc.es; sonia.riveram@udc.es
^2^  Shanghai Elite Sport Training Center, Beiyan Rd 300, Chongming Island, Shanghai, China; felipellanes@gmail.com
^3^  School of Physical Education and Training, Shanghai University of Sport, Shanghai, China; totti_yang57@163.com
^4^  Professional Sports Research Center, Shanghai Research Institute of Sports Science, Shanghai, China

* Correspondence: eduardo.carballeira@udc.es; Tel.: +34-634-476-577

**Abstract:** Monitoring neuromuscular responses and recovery processes in elite athletes is a significant challenge, as tests must be non-disruptive to training schedules while remaining reliable. To address this, we propose studying the Dynamic Strength Index (DSI), which involves calculating the ratio between the peak force of a countermovement jump (CMJ) and the peak force of an isometric mid-thigh pull (IMTP) (1). This method is less time-consuming than the load–velocity curve and does not require weight adjustments between athletes, making it ideal for frequent evaluations of large groups. This study aimed to compare the inter-session reliability of the DSI with the load–velocity curve in elite Chinese judokas. Sixteen judokas (10 males and 6 females, ages: 18.5 ± 2.6 years, weight: 72.7 ± 12 kg, body fat: 13.8 ± 4 kg, muscle mass: 47.5 ± 2.4 kg) participated. Testing took place over five days: three for familiarization and two for actual testing. Each session was separated by 48 h and conducted at the same time of day to ensure inter-day reliability. Athletes abstained from training for 48 h prior to testing and maintained consistent fluid and dietary intake. The two tests were performed on the same day, separated by one hour. The order of the tests was randomized by blocks, with pairs sorted by maximum strength level in IMTP. For the load–velocity multiple-point method, high reliability was found for L_0_ (CV = 3.48%; ICC_3_._1_ = 0.97), V_0_ (CV = 3.53%; ICC_3_._1_ = 0.92), S_L-V_ (CV = 6.46%; ICC_3_._1_ = 0.90), and A_line_ (CV = 5.21%; ICC_3_._1_ = 0.96). For the load–velocity two-point method, high reliability was obtained for L_0_ (CV = 9.44%; ICC_3_._1_ = 0.78), V_0_ (CV = 4.95%; ICC_3_._1_ = 0.88), and A_line_ (CV = 6.09%; ICC_3_._1_ = 0.95), but unacceptable reliability was obtained for S_L-V_ (CV = 14.29%; ICC_3_._1_ = 0.62). The IMTP (CV = 7.91%; ICC_3_._1_ = 0.85) and CMJ (CV = 7.47%; ICC_3_._1_ = 0.90) showed high reliability, but the DSI variable had unacceptable reliability (CV = 11.11%; ICC_3_._1_ = 0.74), although it performed better than a similar test previously reported (2). The most replicable test for monitoring strength performance in elite judokas was the one associated with the load–velocity curve. The two-point method yielded results comparable to the multi-point curve but was more efficient, requiring less time and effort. The reproducibility of the DSI was near the CV and ICC cut-off thresholds, indicating the need for further studies with larger sample sizes to clarify its utility for neuromuscular monitoring.

**Keywords:** neuromuscular monitoring; isometric mid-thigh pull; squat

**Funding:** This research received no external funding.

**References**
  1. Sheppard, J.M.; Chapman, D.; Taylor, K.-L. An Evaluation of a Strength Qualities Assessment Method for the Lower Body. J. Aust. Strength Cond. 2011, 19, 4–10.
  2. Šarabon, N.; Kozinc, Ž.; Marković, G. Force–Velocity Profile during Vertical Jump Cannot Be Assessed Using Only Bodyweight Jump and Isometric Maximal Voluntary Contraction Tasks. Sci Rep-uk 2020, 10, 19127.

### 2.13. Comparison of Full Range and Variable Range of Motion Protocols in Bench Press: Impact on 1RM and Repetitions to Failure

Daniel Cerdán ^1^, Miguel López-Fernández ^1,^*, José Luis Hernández-Davó and Rafael Sabido ^1^

^1^  Sport Sciences Department, Miguel Hernández University, 03202 Elche, Spain; d.cerdan@umh.es (D.C); m.lopezf@umh.es (M.L.-F); rsabido@umh.es (R.S)

* Correspondence: m.lopezf@umh.es; Tel.: +34-690820865

**Abstract:** The application of range of motion (ROM) variability using partial ranges has the potential to facilitate muscle hypertrophy, particularly when the muscle is stretched [2]). Conversely, the efficacy of protocols with ROM variability in improving strength has been investigated, particularly in exercises such as the bench press, where the muscle is shortened. However, the improvements observed were specific to the range of motion trained [1]. To date, no research has been conducted on the impact of introducing variability in ROM with partial stretch on strength levels during basic movements. The aim of this study was to compare the effects of a strength protocol that applies ROM variability with a traditional full ROM protocol on 1RM. A total of nine trained participants completed an eight-week training program focusing on the bench press. Participants were divided into two groups: one that trained traditionally without ROM variability using full range (FULL) and one that varied their range of motion between three possibilities (1/3, 2/3, full range of motion), which was designated as VAR. An indirect repetition maximum (RM) test and repetitions to failure at 70% of the estimated RM were performed. Both groups trained twice a week, performing ten sets of bench presses per week. The initial set was performed as a test at 80% with RPE 8. From this test, O’Connor’s RMt formula was used to calculate the next four sets of six repetitions at 75–77% of the estimated RM. At the end of the eight-week training period, a further analysis was carried out using a linear encoder to determine 1RM and repetitions to failure. A two-factor ANOVA was performed to determine the differences in 1RM and repetitions to failure between the different groups. Statistically significant differences (*p* < 0.05) were observed in the bench press RM test and repetitions to failure before and after the intervention in both groups, although there were no significant differences between the groups. We conclude that the use of ROM variability during bench press exercises under stretching conditions is as effective as using the full range of motion without variability in improving strength levels.

**Keywords:** strength; variability; range of motion

**Funding:** This research received no external funding.

**References**
  1. Martínez-Cava, A., Hernandez-Belmonte, A., Courel-Ibanez, J., Moran-Navarro, R., Gonzalez-Badillo, J. J., & Pallarés, J. G. (2022). Bench press at full range of motion produces greater neuromuscular adaptations than partial executions after prolonged resistance training. The Journal of Strength & Conditioning Research, 36(1), 10–15.
  2. Kassiano, W., Costa, B., Nunes, J. P., Ribeiro, A. S., Schoenfeld, B. J., & Cyrino, E. S. (2023). Which ROMs lead to Rome? A systematic review of the effects of range of motion on muscle hypertrophy. The Journal of Strength & Conditioning Research, 37(5), 1135–1144.

### 2.14. Corrective Path Using Fluiwalk

Domenico Cherubini ^1,^*, Claudio Di Brigida ^1^ Marco De Angelis ^2^ and Massimo Angelozzi ^2^

^1^  Facultad de Deporte. UCAM Universidad Católica de Murcia
^2^  Università degli Studi de L’Aquila, Italy

* Correspondence: dcherubini@ucam.edu

**Abstract**: Walking represents a fundamental human activity that promotes health and prevents a wide range of disorders [1]. It is widely recognized that adopting a regular walking routine can significantly mitigate the risks of developing joint, cardiovascular, and lung diseases, as well as improve metabolism and reduce the risk of developing diabetes in an era where a sedentary lifestyle is becoming increasingly common. Walking goes far beyond the sphere of prevention; it constitutes a key element in protocols for maintaining health as well as for rehabilitation [2]. To help stimulate the body while walking, the market is offering tools that can contribute to a more efficient and effective walk. Among these products are the Fluiwalks (FWs). These are devices made of disc-shaped rubber weights with fluids inside them to be gripped while walking. The inertial push of the liquid content when swinging the arm stimulates a wide swing of the arms and, consequently, a better global postural structure. They aim to increase the intensity of walking and, consequently, calorie consumption. This study was designed to evaluate both the immediate and long-term effects of the use of FW by adults when flat walking. Ten healthy subjects (seven males and three females aged 29.4 ± 6.5 years) participated in the study. They were asked to walk on a Panatta treadmill for 10 min at their most comfortable speed, repeating the test three times: initially without using the FW, then with, and again without the FW to evaluate the lasting effects. During the last minute of the test, the parameters under study were measured. The Metabolimeter (Fitmate PRO Cosmed-Rome, Italy) was used to measure metabolic parameters, including heart and respiratory rate, oxygen consumption, and ventilatory threshold. The Lactate Scout (EKF Diagnostic) was used to measure blood lactate, while the Optogait (Microgait) was used to detect the space–time parameters of gait: step length, step frequency, support, and swing time. To analyze the movements of the upper limbs and trunk, three orthogonally positioned cameras were used. At a metabolic level, the results of this study showed that only respiratory parameters significantly increased between the first test without FW and the second with FW (RF and EVC, *p* = 0.05 and *p* = 0.01, respectively), while the increase in VO2 was not significant. A significant decrease in VO2 was found when subjects repeated the test without FW (*p* = 0.007). Very significant changes were observed in the space–time parameters of gait: an increase in the length (*p* < 0.01) corresponded to a reduction in the frequency of gait (*p* < 0.01), which was maintained even when the test was repeated without FW. These results show a clear modification of gait parameters when FWs are used while walking, even if the modifications at the metabolic level are not evident. A more prolonged exercise duration could provide more precise information.

**Keywords:** Fluiwalk, posture, metabolism, walking

**Funding:** This research received no external funding.

**References**
  1. Murtagh, E. M., Murphy, M. H. & Boone-Heinonen, J. (2010). Walking: the first steps in cardiovascular disease prevention. Current Opinion in Cardiology 25(5):p 490–496. | DOI: 10.1097/HCO.0b013e32833ce972
  2. Lee, I. M., & Buchner, D. M. (2008). The importance of walking to public health. Medicine and science in sports and exercise, 40(7 Suppl), S512–S518. https://doi.org/10.1249/MSS.0b013e31817c65d0.

### 2.15. Effects of Movement Velocity in Squat Training with and Without Blood Flow Restriction

Pedro Jesús Cornejo-Daza ^1,2,3,^*, Juan Sánchez-Valdepeñas ^1,2^, Luis Rodiles-Guerrero ^1,3^, Miguel Sánchez-Moreno ^1,4^, Juan Antonio León Prados ^2^ and Fernando Pareja-Blanco ^1,2^

^1^  Science Based Training Research Group, Department of Sports and Computer Sciences, Universidad Pablo de Olavide, Seville, Spain
^2^  Faculty of Sport Sciences, Department of Sports and Computer Sciences, Universidad Pablo de Olavide, Seville, Spain
^3^  Department of Human Movement and Sport Performance, University of Seville, Seville, Spain
^4^  Department of Physical Education and Sports, University of Seville, Seville, Spain

* Correspondence: pjcordaz@upo.es

**Abstract:** Movement velocity has been shown to be of greater importance during resistance training (RT) than the time under tension for inducing neuromuscular adaptations directed towards improving athletic performance [1]. Despite the potential advantages observed by lifting the load at the maximal voluntary velocity under free-flow (FF) conditions [1], previous RT studies with blood flow restriction (BFR) have been conducted by performing each repetition in a controlled manner [2,3]. Therefore, it is unknown whether movement velocity under BFR conditions could influence the long-term effects, or if this methodology would have similar effects to those observed under FF conditions. Consequently, the aim of this study was to compare the effects of four different RT protocols in the full-squat (SQ) exercise, which differed in the movement velocity (maximum [MaxV] vs. 50% of maximum voluntary velocity [HalfV]) and the blood flow condition (FF vs. BFR) on jump performance, muscle strength, and muscle hypertrophy. Forty-six resistance-trained males were randomly assigned to four training groups that differed in movement velocity (MaxV vs. HalfV) and blood flow condition [FF vs. BFR (50% of arterial occlusion pressure)]. Subjects followed an RT program for 8 weeks (two sessions per week) using the SQ exercise, with similar relative intensity (55–65% of one-repetition maximum, 1RM), number of sets (3), repetitions (6 to 10), and resting time (2 min). The following measurements were taken before and after the RT program: 1) muscle area and volume of vastus lateralis (VLA) assessment, 2) countermovement jump (CMJ) test, and 3) progressive loading test to estimate 1RM in SQ. After the training program, the HalfV-BFR and MaxV-FF protocols increased CMJ height (BFR × VEL × time interaction, *p* = 0.05). However, no “BFR × VEL × time”, “BFR × time”, and “VEL × time” interactions were found in 1RM (*p* > 0.05). MaxV-BFR and HalfV-FF protocols induced higher muscle hypertrophy in the anatomical cross-sectional area at 40% (ACSA40) of the femur length (BFR × VEL × time interaction, *p* = 0.04). Likewise, MaxV-BFR resulted in the largest effect sizes in muscle size after the RT program. Therefore, maximal voluntary velocity in FF and HalfV in BFR conditions increased CMJ performance. MaxV in BFR and HalfV under FF conditions evoked higher muscle hypertrophy than the other protocols. Likewise, MaxV under BFR conditions resulted in the greatest gains in muscle size hypertrophy.

**Keywords:** velocity-based training; intentionality; arterial occlusion pressure

**Funding:** This study was funded by the Ministerio Español de Innovación y Ciencia (AEI/10.13039/501100011033; grant reference PID2020-117915RA-I00). The funders had no role in study design, data collection, analysis, decision to publish, or preparation of the manuscript.

**References**
  1. Pareja-Blanco, F., Rodriguez-Rosell, D., Sanchez-Medina, L., Gorostiaga, E. M., & Gonzalez-Badillo, J. J. Effect of movement velocity during resistance training on neuromuscular performance. Int J Sports Med 2014, 35(11), 916–924
  2. Lixandrão, M. E., Ugrinowitsch, C., Laurentino, G., Libardi, C. A., Aihara, A. Y., Cardoso, F. N., Tricoli, V., & Roschel, H. Effects of exercise intensity and occlusion pressure after 12 weeks of resistance training with blood-flow restriction. Eur J Appl Physiol 2015, 115(12), 2471–2480
  3. Wernbom, M., Apro, W., Paulsen, G., Nilsen, T. S., Blomstrand, E., & Raastad, T. Acute low-load resistance exercise with and without blood flow restriction increased protein signalling and number of satellite cells in human skeletal muscle. Eur J Appl Physiol 2013, 113(12), 2953–2965

### 2.16. Validity of the Individualized Load–Velocity Profile for Predicting One-Repetition Maximum on a Pneumatic Leg Press Device in Males Aged 55–80 Years

Jolien Deboutte ^1^,*, Julian Alcazar ^2,3^, Simon Walker ^4^, Christophe Delecluse ^1^ and Evelien Van Roie ^1^

^1^  Department of Movement Sciences, KU Leuven, Leuven, Belgium; jolien.deboutte@kuleuven.be; chritophe.delecluse@kuleuven.be; evelien.vanroie@kuleuven.be
^2^  GENUD Toledo Research Group, Universidad de Castilla-La Mancha, Toledo, Spain; julian.alcazar@uclm.es
^3^  CIBER of Frailty and Healthy Aging (CIBERFES), Madrid, Spain
^4^  Faculty of Sport and Health Sciences, University of Jyväskylä, Jyväskylä, Finland; simon.walker@jyu.fi

* Correspondence: jolien.deboutte@kuleuven.be; Tel.: +3216328118

**Abstract:** Resistance exercise is the primary therapeutic strategy for counteracting age-related declines in muscle function. To individualize exercise intensity, training loads are often prescribed based on an individual’s one-repetition maximum (1-RM) [1]. Despite the excellent reliability of 1-RM measurements [2], the protocol is time-consuming and may increase the risk of injuries, especially in older adults [3]. Consequently, various approaches for estimating 1-RM, and thus accurate training intensity, have been developed. Considering that movement velocity can accurately predict relative load [4], the aim of this analysis was to determine whether the individual load–velocity (L-v) profile of older males can be used to accurately estimate their 1-RM. Sixty-four males (66.9 ± 5.4 years old) completed explosive concentric leg extensions on a pneumatic leg press device at five pre-fixed increasing loads. The loads and corresponding velocity values were used to develop each individual’s linear L-v regression equation. Following the submaximal protocol, participants performed single repetitions up to 1-RM to establish their measured 1-RM and velocity at 1-RM (V_1RM_). To estimate each individual’s 1-RM, the group’s average V_1RM_ was input into the individual L-v equations. Two estimation approaches were employed: one using the actual V_1RM_ (0.07 ± 0.03 m/s) and the other using a corrected V_1RM_ (0.10 ± 0.07 m/s). When the actual V_1RM_ was used, the estimated 1-RM was significantly higher than the measured 1-RM (−2.4 ± 6.6 kg, *p* = 0.006). However, when the corrected V_1RM_ was used, the estimated 1-RM was not significantly different from the measured 1-RM (−0.5 ± 6.4 kg, *p* = 0.525). Notably, the accuracy of both estimation approaches varied among individuals, 29 with estimation error correlating to the difference between the average and individual V_1RM_. The results indicate that the 1-RM estimation approach using the corrected V_1RM_ can be accurate for older males. However, it may not be suitable for individuals whose V_1RM_ significantly deviates from the group mean.

**Keywords:** muscle strength; resistance training; ageing

**Funding:** E. Van Roie was funded by the Research Foundation Flanders, Belgium (senior postdoctoral fellowship 12Z5720N). J. Alcazar was funded by CIBER—Consorcio Centro de Investigación Biomédica en Red—(CB16/10/00477), Instituto de Salud Carlos III, Ministerio de Ciencia e Innovación, and Unión Europea—European Regional Development Fund.

**References**
  1. Thompson, S.W.; Rogerson, D.; Ruddock, A.; Barnes, A. The Effectiveness of Two Methods of Prescribing Load on MaximalStrength Development: A Systematic Review. *Sports Med* **2020**, *50*, 919–938.
  2. McMaster, D.T.; Gill, N.; Cronin, J.; McGuigan, M. A brief review of strength and ballistic assessment methodologies in sport.
  3. *Sports Med***2014**, *44*, 603–623.
  4. Niewiadomski, W.; Laskowska, D.; Gąsiorowska, A.; Cybulski, G.; Strasz, A.; Langfort, J. Determination and prediction of one repetition maximum (1RM): safety considerations. J Hum Kinet **2008**, 19, 109–120.
  5. González-Badillo, J.J.; Sánchez-Medina, L. Movement velocity as a measure of loading intensity in resistance training. *Int J Sports Med* **2010**, *31*, 347–352.

### 2.17. Perception of Risk Factors for Groin Pain in Football Players: An International Survey Study

Omar de la Calle ^1^, Miguel Pérez-Fernández ^1^, Marcos Quintana-Cepedal ^1,2^, Toni Bailen-Garcia ^1,2^, Beatriz Sanchez-Martinez ^3^ and Hugo Olmedillas ^1,2,^*

^1^  Asturian Research Group in Performance, Readaptation, Training, and Health (AstuRES), Oviedo, Spain; omarfdzdelacalle@gmail.com (O.C.); miguelperezfdez4@gmail.com (M.P-F.); marcosquintana99@gmail.com (M.Q.-C.); tonibailenito@gmail.com (T.B.-G.)
^2^  Department of Functional Biology, University of Oviedo, Oviedo, Spain
^3^  Department of Educational Sciences, University of Oviedo, Oviedo, Spain; bsanchez@uniovi.es (B.S.-M.)

* Correspondence: olmedillashugo@uniovi.es

**Abstract:** Groin injuries are common in multidirectional sports and in football in particular, reaching a seasonal prevalence of up to 53%. An alternative methodology for identifying risk factors involves surveying the population at risk and gathering their viewpoints on the primary factors they believe predispose them to groin injuries. The aim of the present study was to assess the perception of players on potential risk factors for sustaining groin injuries. The hypothesis was that previous groin injury would be perceived as a risk factor for a new injury. Participants (n = 349; 93.5% Europe, 6% America, and 0.6% Africa) were recruited via word of mouth and advertisements placed on social networks by the authors. Two physiotherapists developed the questionnaire, which consisted of 29 items. The 29 items were reviewed by five experts to evaluate the degree of clarity in the formulation of the items for the general population, as well as the degree to which they considered the items to be relevant for evaluating the proposed dimension. Based on the responses of the experts, eight items were eliminated due to redundancy in their content or lack of fit in the dimension to be evaluated. A pilot study was conducted on 29 football players. The final version of the questionnaire consisted of 21 Likert-type items, with five response categories, ranging from one (strongly disagree) to five (strongly agree). The items with the highest rating were weak adductor strength (3.5 [SD = 0.6]), performing strength training (3.4 [SD = 0.7]), access to a physiotherapist (3.3 [SD = 0.7]), and access to strength and conditioning staff (3.2 [SD = 0.8]). Previous groin injury received a value of 2.9 [SD = 0.9]. Our results suggest that groin injuries can be prevented by implementing adductor strengthening programs tailored by conditioning and medical staff.

**Keywords:** injury prevention; adductor muscles; Likert scale

**Funding:** This research received no external funding.

**References**
  1. Quintana-Cepedal M, De La Calle O, Olmedillas H. Can the Copenhagen Adduction Exercise Prevent Groin Injuries in Soccer Players? A Critically Appraised Topic. *J Sport Rehabil*. 2024;33(1):45–48.
  2. Whittaker JL, Small C, Maffey L, Emery CA. Risk factors for groin injury in sport: an updated systematic review. *Br J Sports Med*. 2015;49(12):803–809.
  3. Harøy, J., Wiger, E. G., Bahr, R., & Andersen, T. E. Implementation of the Adductor Strengthening Programme: Players primed for adoption but reluctant to maintain—A cross-sectional study. *Scand J Med Sci Sports*. 2019;*29(8)*, 1092–1100.

### 2.18. Effectiveness of Extracorporeal Shock Wave Therapy in Chronic Achilles and Patellar Tendinopathy: A Randomized Controlled Trial

Pedro Diez ^1,2^, Beltrán González ^3^, Martin González ^3^, Miguel del Valle ^2,4^ and Hugo Olmedillas ^1,2,^*

^1^  Department of Functional Biology, University of Oviedo, Oviedo, Spain; diezsolorzano.p@gmail.com (P.D)
^2^  Asturian Research Group in Performance, Readaptation, Training, and Health (AstuRES), Oviedo, Spain; miva@uniovi.es (M.V.)
^3^  University of Oviedo, Oviedo, Spain
^4^  Department of Cellular Morphology and Biology, University of Oviedo, Oviedo, Spain

* Correspondence: olmedillashugo@uniovi.es

**Abstract:** Tendinopathies are a prevalent condition affecting both the general population and athletes [1]. Their location is related to the sport’s characteristics and demands, arising from a combination of intrinsic and extrinsic factors that reduce the tendon’s ability to adapt to stress [2]. Exercise therapy, along with patient education and load management, is an essential component of treatment [3]. However, the importance of adjunctive therapies is a matter of debate. The aim of this study is to assess the effectiveness of extracorporeal shock wave therapy (EST) versus a combined therapy using ultrasound and massage (US-M) for the treatment of Achilles and patellar tendinopathies. A single-blind randomized controlled trial was conducted for 5 weeks. People suffering from Achilles or patellar tendinopathy were assigned to an experimental group (n = 26) receiving EST or to a control group (n = 23) receiving US-M, both twice a week. Outcome measures were perceived pain (VAS), pain pressure threshold, and functional capacity (VISA). Significant differences were found in favor of the experimental group for VAS (*p* = 0.000) and pain pressure threshold (*p* = 0.032). Regarding VISA, significant differences were found in favor of the experimental group for patients suffering from patellar tendinopathy (VISA-P) (*p* = 0.048), but not for those suffering from Achilles tendinopathy (VISA-A) (*p* = 0.129). From these data, we can conclude that EST is effective for reducing VAS and pain pressure thresholds both in Achilles and patellar tendinopathy while improving function in patellar tendinopathy compared with a combined treatment consisting of US-M.

**Keywords:** Achilles tendinopathy; patellar tendinopathy; extracorporeal shock wave therapy

**Funding:** This research received no external funding.

**References**
  1. Florit D, Pedret C, Casals M, Malliaras P, Sugimoto D, Rodas G. Incidence of tendinopathy in team sports in a multidisciplinary sports club over 8 Seasons. J Sports Sci Med. 2019;18(4):780–788.
  2. Cardoso TB, Pizzari T, Kinsella R, Hope D, Cook JL. Current trends in tendinopathy management. Best Pract Res Clin Rheumatol. 2019;33(1):122–140.
  3. Cook JL, Rio E, Purdam CR, Docking SI. Revisiting the continuum model of tendon pathology: what is its merit in clinical practice and research? Br J Sports Med. 2016;50(19):1187–1191.

### 2.19. The Time Course of Post-Match Physical Capacity Impairments in Professional Soccer: A Systematic Review

Alec M. Drayton ^1,^*, Maziar J. Hamad ^1,2,3^ and Konstantinos Spyrou ^1,2,3^

^1^  UCAM Research Center for High Performance Sport, UCAM Universidad Católica de Murcia, Murcia Spain; amdrayton@alu.ucam.edu; kspyrou@ucam.edu; mjabar@ucam.edu
^2^  Faculty of Sport Sciences, Catholic University of Murcia (UCAM), Murcia, Spain
^3^  Strength and Conditioning Society, Murcia, Spain

* Correspondence: amdrayton@alu.ucam.edu

**Abstract:** Professional soccer often requires players to compete every 72 h, which can lead to notable fatigue and residual damage (1). Greater knowledge of post-match impairments of physical capacities is crucial in the pursuit of maintaining optimal performance and minimizing injury risk (2). Despite previous research investigating the acute and residual effects of match-related fatigue (3), there is a need for a review with tighter constraints (male, professional level, official match, etc.) in order to apply these findings to male professional soccer players. This review aimed to assess the effect of a single soccer match on the physical capacities of professional players, investigating their response at different timepoints: immediately, 24 h, 48 h, and 72 h post-match. A literature search was conducted on the 28th of March 2024 in the following databases: Medline (PubMed), Web of Science, and Scopus. A total of 13 studies were eligible and included in this review. Results show that even after 72 h, professional players still experienced significant physical impairments. While sprint, change of direction (COD), and technical ability had all recovered by 72 h, significant impairments were still observed in vertical jump ability and hamstring strength. This highlights the complexity of post-match fatigue, which relies on a combination of central and peripheral mechanisms. However, even in professional soccer, variations were observed across studies, suggesting that baseline physical fitness levels play a determining role in the duration of post-match impairments. Future research is needed in elite soccer populations to fully understand which specific physical fitness measures (strength, aerobic fitness, power, etc.) most strongly influence post-match physical impairments, as this could help inform future training practices. Nevertheless, practitioners should be aware of the varied individual recovery profiles observed in professional players to optimize player readiness and reduce injury risk.

**Keywords:** soccer; fatigue; recovery; match; neuromuscular

**Funding:** This research received no external funding.

**References**
  1. Nédélec, M., McCall, A., Carling, C., Legall, F., Berthoin, S., & Dupont, G. Recovery in soccer: part I—post-match fatigue and time course of recovery. *Sports Med* (2012), *42*(12), 997–1015.
  2. Carling, C., Lacome, M., McCall, A., Dupont, G., Le Gall, F., Simpson, B., & Buchheit, M. (2018). Monitoring of post-match fatigue in professional soccer: welcome to the real world. *Sports Med (2018)*, *48*(12), 2695–2702.
  3. Silva, J. R., Rumpf, M. C., Hertzog, M., Castagna, C., Farooq, A., Girard, O., & Hader, K. Acute and residual soccer match-related fatigue: A systematic review and meta-analysis. *Sports Med* (2018), *48*(3), 539–583.

### 2.20. Inter-Unit Reliability of Firstbeat Sports Sensors as an External Load Monitoring Device for Basketball Training Sessions

Davide Ferioli ^1,^*, Ermanno Rampinini ^2^, Fabio Trimarchi ^1^, Debora Di Mauro ^1^ and Daniele Conte ^3^

^1^  Department of Biomedical Sciences, Dental Sciences, and Morpho-Functional Imaging, University of Messina, Messina, Italy
^2^  Human Performance Laboratory, MAPEI Sport Research Center, Olgiate Olona, Varese, Italy
^3^  Department of Movement, Human and Health Sciences, University of Rome “Foro Italico”, Rome, Italy

* Correspondence: davide.ferioli@unime.it

**Abstract:** Monitoring and managing training load are key aspects of the training process in basketball [1]. As such, various brands have developed wearable micro-technologies, such as Inertial Movement Units, to effectively monitor players’ external load. While most of these devices are usually located between the scapulae by dressing customized vests with reported inter-unit reliability [2], the Firstbeat Technologies Oy (Jyväskylä, Finland) has designed a chest belt heart rate monitor incorporating an accelerometer that also provides measurements of external load named “Movement Load”, derived from the triaxial accelerations the player performs. While Firstbeat Sports sensors are widely used in professional basketball, no information is available about their inter-unit reliability, which is key to ensuring a valid monitoring process. Therefore, the purpose of the present study was to measure the inter-unit reliability of the Movement Load derived from the Firstbeat Sports sensors during basketball training sessions. Eight professional male basketball players (age: 26 ± 5 years, height: 199 ± 8 cm, body mass: 97 ± 8 kg) competing in the same professional basketball team (Italian first division, Serie A) wore—at the same time—two Firstbeat Sports sensors during team training sessions in the 2023/2024 season. Firstbeat Sports sensors were firmly affixed to players’ chests roughly at the base of the sternum via textile straps, with sensor pairs randomly assigned to each player before the beginning of practice. Movement Load was calculated using the Firstbeat Sports software (version 2.50.3; Firstbeat Technologies Oy; Jyväskylä, Finland) as the sum of accelerations across the three movement axes using the tri-axial accelerometer component sampling at 50 Hz, as previously reported [3]. A total of 50 individual training sessions were recorded, with players wearing the sensor pairs on one or multiple occasions (range: 1 to 21 individual sessions for each player) across the data collection period. The typical error expressed as a coefficient of variation (CV) and the intraclass correlation coefficient (ICC) with 95% confidence intervals were calculated using customized spreadsheets. The following results were obtained: CV = 2.51% (2.09; 3.14) and ICC = 0.99 (0.99; 1.00). The Movement Load derived from the Firstbeat Sports sensor displayed high inter-unit reliability during professional basketball training sessions. Thus, Firstbeat Sports sensors can be used to compare players’ external loads (i.e., Movement Load data) and, when it is not possible to use the same device for the same player, they can be used interchangeably between players to monitor the Movement Load in professional basketball.

**Keywords:** team sport; training load; accelerometer; tracking device.

**Funding:** This research received no external funding.

**References**
  1. Fox, J.L., Scanlan, A.T., & Stanton, R. A review of player monitoring approaches in basketball: current trends and future directions. *J Strength Cond Res.* **2017**, 31(7), 2021–2029.
  2. Crang, Z.L., Duthie, G., Cole, M.H., Weakley, J., Hewitt, A., & Johnston, R.D. The validity and reliability of wearable microtechnology for intermittent team sports: A systematic review. *Sports Med*. **2021**, 51(3), 549–565.
  3. Ferioli, D., Alcaraz, P.E., Freitas, T.T., Trimarchi, F., Conte, D., Formica, L., Chung, L.H. & Scanlan, A.T. The reliability and discriminant validity of physical, technical, and perceptual-physiological measures during a game-specific basketball activity simulation protocol. *Front. Psychol.* **2024**, *accepted*.

### 2.21. Acute Effects of High vs. Low Intensity Strength Training on Sleep Quality in Women with Fibromyalgia: A Randomized Crossover Study

Fernández Calero Marta ^1,2^, Abellán-Aynés Oriol ^3^, Manonelles Marqueta Pedro ^1^, López-Plaza, Daniel ^4^, Andreu Caravaca ^5,6^ and Quero-Calero Carmen Daniela ^1,5,^*

^1^  Faculty of Physiotherapy, Podiatry and Occupational Therapy, UCAM Universidad Católica de Murcia, 30107 Murcia, Spain; mifernandez2@ucam.edu
^2^  International Chair of Sport Medicine, UCAM, Universidad Católica de Murcia, 30107 Murcia, Spain; mifernandez2@ucam.edu; pmanonelles@ucam.edu; cdquero@ucam.edu
^3^  Department of Health Sciences, Public University of Navarre (UPNA), 31008 Pamplona, Spain; abellanoriol@gmail.com
^4^  Department of Education, Health Research Center, University of Almeria, 04120 Almeria, Spain; dlplazapal@ual.es
^5^  Facultad de Deporte, UCAM, Universidad Católica de Murcia, 30107 Murcia, Spain; landreu@ucam.edu, cdquero@ucam.edu
^6^  Sports Physiology Department, Faculty of Health Sciences. Catholic University of Murcia. Murcia. Spain; landreu@ucam.edu

* Correspondence: cdquero@ucam.edu

**Abstract:** Fibromyalgia (FM) is a prevalent chronic pain disorder often accompanied by a range of symptoms, with sleep disturbances being notably prominent. Patients frequently endure disrupted sleep, which exacerbates their overall fatigue, stiffness, and recurrent pain episodes. Additionally, individuals with FM often suffer from psychological distress and gastrointestinal disorders, all of which contribute to the complexity and severity of the condition. This study investigates the impact of acute strength training at different intensities on sleep quality, functional capacity, and strength in women with fibromyalgia. A randomized crossover design was employed, involving 12 female participants diagnosed with FM who underwent strength training at 50% and 85% of their one-repetition maximum (1RM). Sleep quality was assessed using the Karolinska Sleepiness Diary (KSD), which evaluated factors such as overall sleep quality, sleep comfort, ease of falling asleep, awakening, ease of waking up, feeling of rest, and sufficiency of sleep. The results indicated that high-intensity training (85% RM) significantly enhanced overall sleep quality and ease of falling asleep compared to moderate-intensity training (50% RM). Specifically, the mean sleep quality score improved from 2.72 ± 1.1 to 3.25 ± 1.21 (*p* = 0.003), and the ease of falling asleep improved from 2.63 ± 1.36 to 3.16 ± 1.02 (*p* = 0.035). No significant differences were observed in other sleep parameters, including sleep comfort, awakening, ease of waking up, feeling of rest, and the perception of having had enough sleep. The findings suggest that both high and low-intensity strength training can benefit women with FM, but the effects vary depending on the specific outcomes measured, with high-intensity training proving more effective in enhancing certain aspects of sleep quality. Therefore, the choice of training intensity should be tailored to individual capabilities and desired outcomes, with a gradual progression recommended to maximize benefits while minimizing discomfort. These results highlight the potential of customized strength training programs to improve sleep quality and functional capacity in FM patients, contributing to better management of the condition and overall quality of life.

**Keywords:** fibromyalgia; resistance training; sleep quality

**Funding:** This research received no external funding.

**References**
  1. Ericsson, A.; Palstam, A.; Larsson, A.; Löfgren, M.; Bileviciute-Ljungar, I.; Bjersing, J.; Mannerkorpi, K. Resistance exercise improves physical fatigue in women with fibromyalgia: a randomized controlled trial. *Arthritis Res. Ther*. **2016**, 18, 1–12.
  2. Andrade, A.; Vilarino, G.T.; Bevilacqua, G.G. What is the effect of strength training on pain and sleep in patients with fibromyalgia? *Am. J. Phys. Med. Rehabil.* **2017**, 96, 889–893.
  3. Andrade, A.; Sieczkowska, S.M.; Vilarino, G.T. Resistance training improves quality of life and associated factors in patients with fibromyalgia syndrome. *PM&R* **2019**, 11, 703–709.
  4. Munguía-Izquierdo, D.; Legaz-Arrese, A. Assessment of the effects of aquatic therapy on global symptomatology in patients with fibromyalgia syndrome: a randomized controlled trial. *Arch. Phys. Med. Rehabil*. **2008**, 89, 2250–2257.

### 2.22. Global Systematic Review of the Role of Genetics in Sports Performance

Samuel Fernández Lorenzo ^1,^*, Cristian Marín Pagán ^2^, Francisco Javier Martínez Noguera ^2^ and Javier Escobar Cubiella ^1^

^1^  SABARTECH S.L, Science Park of the Universidad de Valencia, 46980 Valencia, Spain
^2^  High Performance Sports Research Centre, Universidad Católica San Antonio de Murcia, 30107 Murcia, Spain

* Correspondence: sfernandez29@alu.ucam.edu; Tel.: +34-684623376

**Abstract:** The role of genetics in sports performance is associated with many unknowns, from its potential impact on athletic performance to which genes are involved [1]. From the identification of the first polymorphisms to the present day [2,3], the number of markers has grown exponentially [4]; therefore, in this study, we conducted a systematic review with the aim of identifying possible markers that affect athletic performance. The PRISMA guidelines were followed in the preparation of this systematic review. PubMed and Web of Science databases were searched using key terms, such as “sport”, “athlete”, “athletic performance”, “variant”, “gene”, “power”, “endurance”, “strength”, and “power”. A total of 632 articles were initially identified. Animal studies, publications in languages other than English, and studies with N < 20 were excluded. In addition, articles on nutrigenetics, epigenetics, gene doping, methylation, exercise biomarkers, and genetic diseases were also excluded, and only articles on the possible impact of markers or genes on performance were selected. Articles reporting the following study types were selected: (A) Case–control studies: athletes versus sedentary or similar subjects. (B) Observational studies on injuries. (C) Intervention studies in healthy subjects: improvement of performance or fitness based on genetics. (D) Meta-analyses or other reviews of genes or markers at the performance level. Finally, 164 articles were selected, identifying approximately 170 markers in more than 100 genes. *ACTN3* and *ACE* are the main genes studied in sports performance, with 43 articles publishing data on *ACTN3* and 33 of these showing some type of association between it and performance, and 35 articles publishing data on *ACE* and 22 of these showing an association between it and performance. *PPAR* is the most studied gene family (more than 20 articles), highlighting rs4253778 in the *PPARA* gene and rs8192678 in the *PPARGC1A* gene. In terms of injuries, genes in the collagen family are the most studied (eight studies), with disparate results in terms of their possible impacts on increasing or decreasing predisposition to injuries. Less common markers have also been identified, such as rs3213537 in *CPNE5*, rs516115 in *DCN*, and rs3834129 in *CASP8*, with only one article published on each marker. This review provides an overview of the state of genetics in sports performance. Some limitations of this review, such as underestimation of the number of markers associated with injuries due to the search filters used, should be mentioned.

**Keywords:** sports; genetics; athletes; genomics; physical performance

**Funding:** This research was funded by Universidad San Antonio Católica de Murcia (UCAM) in collaboration with the biotechnological company SABARTECH S.L. through the development of a Doctorate with Industrial Mention.

**References**
  1. Miyamoto-Mikami, E.; Zempo, H.; Fuku, N.; Kikuchi, N.; Miyachi, M.; Murakami, H. Heritability estimates of endurance-related phenotypes: A systematic review and meta-analysis. *Scand J Med Sci Sports* **2018**, *28(3)*, 834–45.
  2. Gayagay, G.; Yu, B.; Hambly, B.; Boston, T.; Hahn, A.; Celermajer, D. S.; Trent, R. J. Elite endurance athletes and the ACE I allele--the role of genes in athletic performance. *Hum Genet* **1998**, *103(1)*, 48–50.
  3. North, K. N.; Yang, N.; Wattanasirichaigoon, D.; Mills, M.; Easteal, S.; Beggs, A. H. A common nonsense mutation results in alpha-actinin-3 deficiency in the general population. *Nat Genet* **1999**, *21(4)*, 353–354.
  4. Semenova, E.A.; Hall, E.C.R.; Ahmetov, I.I. Genes and Athletic Performance: The 2023 Update. *Genes* **2023**, *14*, 1235.

### 2.23. Differences in the Genetic Profiles of Spanish Elite Athletes: Whole-Genome Analysis for the Identification of Markers with an Impact on Sport Performance

Samuel Fernández Lorenzo ^1,^*, Cristian Marín Pagán ^2^, Francisco Javier Martínez Noguera ^2^, Javier Escobar Cubiella ^1^ and Lorena Ponce Ruiz ^1^

^1^  SABARTECH S.L, Science Park of the Universidad de Valencia, 46980 Valencia, Spain
^2^  High Performance Sports Research Centre, Universidad Católica San Antonio de Murcia, 30107 Murcia, Spain

* Correspondence: sfernandez29@alu.ucam.edu; Tel.: +34-684623376

**Abstract:** Sports performance is a complex phenotypic trait that is influenced by multiple factors, including genetics [1]. Despite this, there are still many unknowns about the extent of the impact of genetics on performance, due, among others, to limitations such as access to information, the study population, or the heterogeneity of the sample [2]. In addition, although tools and technologies are now available for more in-depth analyses, there is still a reliance on the study of a few markers. Therefore, the present study aimed to determine possible genetic associations using whole-genome sequencing of a cohort of Spanish elite athletes. Thirty-seven athletes from different sports modalities were recruited for this study. All athletes included in the study were of Caucasian–Mediterranean descent (Spain) and participated in top-level national and international competitions. The athletes were grouped according to their discipline: power/sprint (*P*), consisting of sprinters (N = 17); and endurance (*E*), consisting of long-distance runners, race walkers, and trail runners (N = 25). Genetic sampling was performed using buccal swabs, and sequencing was performed using DNA Microarray and Whole Genome Array. Genotypic frequencies of the analyzed markers were obtained, and Pearson’s Χ2 test was performed to identify possible differences. Statistical analyses were performed with R v.4.4.0. Significant associations were found in the rs1870377 polymorphism in the *KDR/VEGFR2* gene, with the **A** allele over-represented in endurance athletes (*P*: T = 94.1%, A = 5.9%; *E*: T = 66%, A = 34% (*p* < 0.005)); rs1535628 (**G**) in *GRIN3A* over-represented in endurance athletes (*P*: G = 82. 4%, A = 17.6%; *E*: G = 98%, A = 2% (*p* < 0.03)); rs1805065 (**C**) in *SLC6A2* over-represented in power/sprint athletes (*P*: G = 100%; *E*: C = 72%, T = 28% (*p* < 0. 002)); and rs3128575 (**T**) and rs4504708 (**G**) in *COL5A1* over-represented in power/sprint athletes (*P*: C = 52.9%, T = 47.1%; *E*: C = 82%, T = 18% (*p* < 0.009). *P*: T = 58.8%, G = 41.2%; *E*: T = 84%, T = 16% (*p* < 0.02); respectively). In addition, significant differences at the genotype level were observed in rs1815739 (*ACTN3*), rs8111989 (*CKM*), rs16944 (*IL-1B*), rs2256292 (*NNMT*), rs7305115 (*TPH2*), and rs659366 (*UCP2*), with *p* < 0.03 in all cases. Our results seem to concur with results previously reported in the literature [3,4], although they should be interpreted with caution due to the sample size of this study. To further investigate the factors that make an athlete stand out from the rest, we plan to continue with this line of research by comparing elite and non-elite athletes from similar disciplines and identifying possible differences in a population with less intrinsic randomness in the sample.

**Keywords:** sport; genetics; athletes; genomics; physical performance

**Funding:** This study was funded through the Universidad San Antonio Católica de Murcia (UCAM) in collaboration with the biotechnological company SABARTECH S.L. through the development of a Doctorate with Industrial Mention.

**References**
  1. Miyamoto-Mikami, E.; Zempo, H.; Fuku, N.; Kikuchi, N.; Miyachi, M.; Murakami, H. Heritability estimates of endurance-related phenotypes: A systematic review and meta-analysis. *Scand J Med Sci Sports* **2018**, *28(3)*, 834–45.
  2. Semenova, E.A.; Hall, E.C.R.; Ahmetov, I.I. Genes and Athletic Performance: The 2023 Update. *Genes* **2023**, *14*, 1235.
  3. Guilherme, J. P. L. F.; Bigliassi, M.; Lancha Junior, A. H. Association study of SLC6A2 gene Thr99Ile variant (rs1805065) with athletic status in the Brazilian population. *Gene* **2019**, *707*, 53–57.
  4. Chen, C.; Sun, Y.; Liang, H.; Yu, D.; Hu, S. A meta-analysis of the association of CKM gene rs8111989 polymorphism with sport performance. *Biol Sport* **2017**, *34(4)*, 323–330.

### 2.24. Impact of Menopause on Rate of Force Development and Maximal Isometric Strength

David Garcia-Albín ^1,2,3,4^, Coral Sánchez-Martín ^1,2,3,4^, Héctor Gutiérrez-Reguero ^1,2,3,4^, Mikel García-Aguirre ^1,2,3,4^, Miguel Muñoz-Muñoz ^1,2,3,4^, Iván Baltasar-Fernández ^1,2,3,4,5^, Ignacio Ara ^1,2,3,4^, Julián Alcázar ^1,2,3,4^, Francisco J. García-García ^2,3,4,6^ and Luis M. Alegre ^1,2,3,4,^*

^1^  GENUD Toledo Research Group, Faculty of Sports Sciences, University of Castilla-La Mancha, Toledo, Spain
^2^  CIBER on Frailty and Healthy Aging (CIBERFES), Instituto de Salud Carlos III, Madrid, Spain
^3^  Grupo Mixto de Fragilidad y Envejecimiento Exitoso UCLM-SESCAM, Universidad de Castilla-La Mancha, Servicio de Salud de Castilla-La Mancha, Toledo, Spain
^4^  Instituto de Investigación Sanitaria de Castilla-La Mancha (IDISCAM), Junta de Comunidades de Castilla-Mancha (JCCM), Toledo, Spain
^5^  Faculty of Health Sciences, University of Castilla-La Mancha, Talavera de la Reina, Spain
^6^  Geriatric Department, Complejo Hospitalario Universitario de Toledo, Toledo, Spain

* Correspondence: luis.alegre@uclm.es

**Abstract:** Menopause is a critical event for women, marked by a significant decline in estrogen levels. This hormonal change negatively impacts cardiovascular, vasomotor, genitourinary, and bone health [1]. Additionally, alterations in muscle mass and function associated with menopause have been reported [2]. However, existing literature exploring the specific impact of menopause on the Rate of Force Development (RFD) is sparse. This study aimed to investigate the influence of menopause on Maximal Isometric Force (MIF) and RFD. One hundred thirty-seven women were divided into two groups: premenopausal women (n = 74) and postmenopausal women (n = 63) following the STRAW criteria [3]. Participants performed leg extension exercises to assess RFD and MIF using a horizontal leg press machine and a force plate, with the knee angle fixed at 120 degrees. Each participant performed four maximal 4-s contractions as fast and strong as possible. RFD was calculated as the linear slope of the time–force curve between each selected time point (100, 200, 300, 400, and 500 ms), and between 100 ms intervals (100–200, 200–300, 200–300, 300–400, 400–500 ms). MIF was the highest force value registered during the contractions. For statistics, an analysis of covariance (ANCOVA) was employed to assess the effects of menopause on RFD, adjusting for participant age. Significant differences in RFD were found between premenopausal and postmenopausal women at 400 ms (2411 ± 85 N/s vs. 2168 ± 76 N/s; *p* = 0.043), 500 ms (2067 ± 74 N/s vs. 1845 ± 66 N/s; *p* = 0.033), and during the 300–400 ms (1387 ± 100 N/s vs. 1002 ± 89 N/s; *p* = 0.007) and 400–500 ms (699 ± 63 N/s vs. 549 ± 56 N/s; *p* = 0.005) intervals. In contrast, no significant differences were observed at MIF and the earlier time points of RFD. This study shows that menopause affects the later phases of RFD, particularly 400 and 500 ms from contraction onset and during the 300–400 ms and 400–500 ms intervals, which are more correlated with the structural factors of the muscle. This reduction could increase the risk of falls or decrease joint stabilization capacity during dynamic actions. Therefore, based on the findings of this study, training to improve RFD in postmenopausal women is recommended, particularly training focused on muscle mass gain and impact exercises.

**Keywords:** estrogens; muscle function; hormone; maximum voluntary contraction

**Funding:** This work was supported by CIBER—Consorcio Centro de Investigación Biomédica en Red—(CB16/10/00477 and CB16/10/00456); Instituto de Salud Carlos III, Ministerio de Ciencia e In- 43 novación, and Unión Europea—European Regional Development Fund; Plan Propio de Investi- 44 gación of the University of Castilla-La Mancha and FEDER funds from the European Union (2022- 45 GRIN-34296); Ministerio de Universidades of the Government of Spain (grant number FPU21/04717); and Programa Investigo (ref: 2024-INVGO-12359 and ref:2021-COB-10257). It was also funded by grants from the Instituto de Salud Carlos III (PI18/00972) and the Government of Castilla-La Mancha (SBPLY/19/180501/000312).

**References**
  1. Duralde ER, Sobel TH, Manson JE. Management of perimenopausal and menopausal symptoms. BMJ. 2023 Aug 8;382:e072612
  2. Yoh K, Ikeda K, Horie K, Inoue S. Roles of Estrogen, Estrogen Receptors, and Estrogen-Related Receptors in Skeletal Muscle Regulation of Mitochondrial Function. Int J Mol Sci. 2023 Jan 17;24(3):1853.Harlow SD, Gass M, Hall JE, Lobo R, Maki P, Rebar
  3. RW, Sherman S, Sluss PM, de Villiers TJ; STRAW 10 Collaborative Group. Executive summary of the Stages of Reproductive
  4. Aging Workshop + 10: addressing the unfinished agenda of staging reproductive aging. Meno-pause. 2012 Apr;19(4):387–95.

### 2.25. Gluteal Activation and Sprint Performance in Team Sports: A Practical Reflection

**Noel Gil-Lopez ^1^ and Raul Martinez-Santos ^2^**

^1^  University of the Basque Country, UPV/EHU; ngil030@ikasle.ehu.eus
^2^  University of the Basque Country, UPV/EHU, GIKAFIT; raul.martinezdesantos@ehu.es

* Correspondence: ngil030@ikasle.ehu.eus

**Abstract:** Effective muscle recruitment strategies are fundamental for enhancing athletic performance, particularly during explosive movements like sprinting, which is crucial in team sports that require complex, multidirectional actions under time constraints. Optimizing motor patterns, including muscle symmetry and coactivation, is essential to improve acceleration and minimize injury risks. The gluteal muscles play a pivotal role in generating power and stability during sprints and changes of direction. Pre-activation exercises that mimic specific movement patterns can positively impact sprint performance, particularly when targeted at improving hip function. However, the impact of alternating knee-dominant and hip-dominant strength training sessions on hip muscle activation patterns remains uncertain. This raises critical questions for practitioners: does focusing on knee-dominant exercises during a training session negatively alter hip activation patterns in team sport athletes, and could integrating both hip and knee-dominant exercises during the same session mitigate potential adverse effects on sprint performance? Addressing these questions through targeted research could provide valuable insights for optimizing training strategies in athletic populations.

**Keywords:** training; strength

**Funding:** This research received no external funding.

**References**
  1. Pandy MG, Lai AKM, Schache AG. How muscles maximize performance in accelerated sprinting. Scand J Med Sci Sports. 2021;31(10):1882–96.
  2. Goller M, Quittmann OJ, Alt T. How to activate the glutes best? Peak muscle activity of acceleration-specific pre-activation and traditional strength training exercises. Eur J Appl Physiol. 2024;1–13. Available from: https://link.springer.com/article/10.1007/s00421-023-05400-3
  3. Mills M, Barnett F. Effect of restricted hip flexor muscle length on hip extensor muscle activity and lower extremity biomechanics in college-aged female soccer players. The International Journal of Sports Physical Therapy. 2015;10(7):946.
  4. Donald Neumann. Kinesiology of the Hip: A Focus on Muscular Actions. Journal of orthopedic & sports physical therapy. 2010;40(2). Available from: www.jospt.org

### 2.26. Short-Term Strength Training Primarily Induced Spinal Adaptations in Young and Older Adults

Gonzalo Gomez-Guerrero ^1,^*, Janne Avela ^1^, Esa Pihlajamäki ^1^, Ilkka Jussila ^1^, Fu-Yu Deng ^1^, Dawson Kidgell ^2^, Juha Ahtiainen ^1^, and Simon Walker ^1^

^1^  NeuroMuscular Research Center and Faculty of Sport and Health Sciences, University of Jyväskylä, Finland
^2^  School of Primary and Allied Health Care, Monash University, Melbourne, VIC, Australia

* Correspondence: gogomezg@jyu.fi

**Abstract:** During aging, the neuromuscular system undergoes reductions in brain volume, decreases in motor neurons, and loss of muscle size. Collectively, these factors lead to a decrease in strength, power, and muscle activation. Although strength training is a good countermeasure for this decline, there is still limited evidence regarding the specific loci of adaptation within motor pathways responsible for the increase in strength in older adults [1]. Eleven healthy young (22–34 years) and ten healthy older (66–80 years) adults participated in five testing sessions during a 7-week resistance training and detraining program. Lumbar-evoked potentials (LEPs) and motor-evoked potentials (MEPs) were collected during brief contractions at 20% and 60% of the maximum voluntary contraction (MVC). Stimulator output was adjusted to elicit LEPs of 25% of the maximum compound action potential (M-max) at rest. Transcranial magnetic stimulator (TMS) output was adjusted to elicit MEPs at 120%, 140%, and 160% of the active motor threshold (aMT). Ten stimuli were delivered in a randomized order for each condition. Whole-body resistance training consisted of five exercises performed twice a week, including five sets of knee extensions and three sets of leg presses. The young adults had greater MVC ~63 N∙m (*p* = 0.006) and 1RM ~50 kg (*p* = 0.001), as well as lower aMT ~9% (*p* = 0.030) than the older adults. Significant time*group interactions were observed for several variables. Pre- to post-intervention: MVC increased (young: +8 N∙m, *p* = 0.051; older: +13 N∙m, *p* = 0.014), 1RM increased (young: +16 kg, *p* < 0.001; older: +7 kg, *p* = 0.018), and LEP amplitude increased (young: + 0.174, *p*  <  0.001) during 20% MVC. Conversely, there was a decrease in LEP amplitude (−0.241, *p*  <  0.001) at 20% MVC and MEP amplitude at 120% (−0.157, *p*  =  0.034), 140% (−0.196, *p*  =  0.026), and 160% (−0.210, *p*  =  0.006) aMT during 60% MVC trials in older adults. The 7-week training program significantly increased maximum strength in both groups. At baseline, older individuals exhibited lower levels of strength and higher levels of cortico-spinal excitability compared to the younger group. Additionally, while the older group experienced a concomitant decline in cortico-spinal and spinal excitability, the younger group showed an increase in spinal excitability. The results suggest early neural adaptations to short-term strength training occur primarily at the spinal level by enhancing motor neuron output. In older adults, this may involve better synchronization of inhibitory pathways, while in younger adults, increased motor unit recruitment and/or firing rate may be key.

**Keywords:** resistance training; aging; lower limbs

**References**
  1. Siddique, U; Frazer, A.K.; Avela, J; Walker, S.; Ahtiainen, J.P., Howatson, G.; Tallent, J. Kidgell, D.J. Determining the cortical, spinal and muscular adaptations to strength-training in older adults: A systematic review and meta-analysis Ageing Res Rev. **2022**, 82, 101746.

### 2.27. An Exploration of Contact Improvisation from the Somatic Contributions of Aikido, Tuishou, and Kinomichi

Sebastián Gómez-Lozano ^1,^*, Ningyi Zhang ^1^ and Alfonso Vargas-Macías ^2^

^1^  Performing Arts Research Group, UCAM; sglozano@ucam.edu, zhangningyi0@gmail.com
^2^  Telethusa Centre for Flamenco Research, Cádiz, Spain; vargas@flamencoinvestigacion.es

* Correspondence: sglozanol@ucam.edu

**Abstract:** Contact improvisation is a dance form that was founded by Steve Paxton in 1972 and is influenced by Aikido and Taijiquan. It developed mainly in New York, where masters such as Yoshimitsu Yamada and Zheng Manqing established themselves. The Canadian choreographer Mark Young has also incorporated Tuishou and Taijiquan in pairs over the last two decades. In North America, this couple’s dance has grown academically and culturally, while in Europe, its practice has obscured the origins of these influences. This dissertation aims to identify the somatic contributions of Aikido, Tuishou, and Kinomichi that are transferable to the health field of contact improvisation. Systematic reviews were conducted using the PRISMA (Preferred Reporting Items for Systematic Reviews and Meta-Analyses) model to accomplish this goal. The review identified a number of common therapeutic and rehabilitative factors between modern martial arts and contact improvisation. Based on this analysis, the psychotherapeutic aspects of Aikido were identified, and the areas of application of Tuishou were described. Furthermore, Kinomichi was found to be an art of martial origin that exposes the therapeutic benefits of Aikido. In conclusion, this study shows that the somatic contributions to contact improvisation have a common aspect referred to as ‘neuromuscular repatterning’. This allows for a conscious rebalancing of the individual’s physical effort during athletic or artistic performance.

**Keywords:** proprioception; rehabilitation; neuromuscular repatterning

**Funding:** This research received no external funding.

**References**
  1. De Baets, G.A.; Van Praet, E. Aikido’s self-regulation and co-regulation: a promising embodied pedagogy for intercultural communication training. *Sport Soc* **2023**, *27*, 1–24.
  2. Delva-Tauiliili, J. Does brief aikido training reduce aggression of youth? Percept Mot Skills **1995**, 80, 297-298.
  3. Gómez-Lozano, S.; García-Sottile, M.E.; Zhang, N.; Moriggi, S.; León, K.; Vargas-Macías, A. Kinomichi, the therapeutic Aikido. A Systematic Review. Cultura, Ciencia y Deporte **2024**, *19*, 65–79.
  4. Krasnow, D. CI Training: The merger of conditioning and imagery as an alternative training methodology for dance. *Med Problem Perform Ar* **1997**, *12*, 3–8.

### 2.28. Criteria-Based Progression in the Late Stage Rehabilitation of a Professional Soccer Player After Peroneal Tendon Injury

Javier A. González Alcántara ^1,2,4,^* and Jordi Sorlí Guerola ^1,3^

^1^  UCAM Universidad Católica de Murcia 30830 Murcia, Spain; jagonzalez08@alu.ucam.edu (J.A.G.A.); jorsor18@hotmail.com (J.S.G.)
^2^  Servicios Médicos Sevilla FC 41005 Sevilla, Spain
^3^  ervicios Médicos Valencia CF 46010 Valencia, Spain
^4^  Departamento de Educación Física y Deporte, Universidad de Sevilla 41013 Sevilla, Spain

* Correspondence: javireadyfisio@gmail.com

**Abstract:** Peroneal tendon injury is one of the main causes of ankle pain and instability in the sports population (1), particularly in sports that require change of direction, such as soccer. It usually occurs when these muscles suddenly contract eccentrically (2). There is no clear consensus on the most appropriate treatment and rehabilitation for this very specific injury (3), and the range of time back to sport varies widely, from 1.2 to 12 months. The objective of this work was to analyze the effect of a 4-week training program based on a prior evaluation using technological devices during the late-stage rehabilitation of a professional soccer player following this injury. An under-23 male soccer player was analyzed during the final phase of rehabilitation following surgery on the peroneus brevis tendon and peroneus retinaculum of the left ankle. The injury occurred in an indirect contact mechanism during training. Three tests were conducted: single-leg drop jump together with surface electromyography, and a COD test (quantitative and qualitative). Various rehabilitation options were proposed based on specific objectives, including isometric work on the injured muscles, resistance training, technically learning the COD in the field, and plyometric, dynamic, and integrated core training. The main finding of this work is that the proposed rehabilitation program improved contact time, eccentric strength, peak landing force, and muscle activation in a single-leg drop jump task in the professional soccer player. Through the results obtained in this work, we can conclude that criteria-based work after prior evaluation during late-stage rehabilitation improved contact time, eccentric strength, peak landing force, and muscle activation in a single-leg drop jump task in a professional male soccer player after a peroneal tendon injury following the proposed 4-week program.

**Keywords:** peroneal tendons; soccer; return to play; criteria-based progression

**Funding:** This research received no external funding.

**References**
  1. Maroc M, Khatab Z, Moueqqit O, Abdeljaouad N, Yacoubi H. Peroneal tendon dislocation: A report of two cases. *Cureus*. **2023**;15(2):e34949.
  2. van Dijk PAD, Gianakos AL, Kerkhoffs GMMJ, Kennedy JG. Return to sports and clinical outcomes in patients treated for peroneal tendon dislocation: a systematic review. *Knee Surg Sports Traumatol Arthrosc*. **2016**;24(4):1155–64.
  3. van Dijk PAD, Lubberts B, Verheul C, DiGiovanni CW, Kerkhoffs GMMJ. Rehabilitation after surgical treatment of peroneal tendon tears and ruptures. Knee Surg Sports Traumatol Arthrosc. **2016**;24(4):1165–74.

### 2.29. Assessment of Finger Flexor Muscle Quality in Climbers vs. Non-Climbers

González-Martín, D. ^1,^*, Santos-Pérez, J. ^1^, Reyes-Merino, F. ^1^, Sanchez-Garcia, N. ^1^ and Gallego-Selles, A. ^2^

^1^  University of León: Campus Vegazana s/n 24071, Leon, Spain; dgonzm10@estudiantes.unileon.es
^2^  Department of Physical Education and Research Institute of Biomedical and Health Sciences (IUIBS), University of Las Palmas de Gran Canaria, Las Palmas de Gran Canaria, Spain

* Correspondence: dgonzm10@estudiantes.unileon.es; Tel.: +34-635312079

**Abstract:** The importance of finger strength in sport climbing is well documented, being crucial for performance [1,2]. Methods such as hang tests and climbing-specific dynamometers are reliable and valid for measuring this strength [3], although they may not capture all muscular adaptations to a training stimulus. Measuring muscle quality (MQ) could reflect the functional sum of physiological changes in response to training adaptations [4]. This study aimed to measure, for the first time, the MQ of finger flexors in climbers and non-climbers, describing muscle quality in men and women and evaluating its validity for assessing climbers. Maximum isometric finger strength was measured in three tests with different grips: fixed-arm extension, fixed-arm half-crimp, and standing half-crimp. To estimate the muscle mass of the finger flexors, dual-energy X-ray absorptiometry (DXA) was used to estimate the lean mass of the forearm. The sample included non-climbers and climbers of different levels. The results were analyzed by sex and skill level. The results showed that non-climbing women had higher muscle quality than non-climbing men. Among climbers, women also exhibited higher muscle quality than men, although with no significant differences. Advanced climbers exhibited the highest MQ (47.17 ± 9.8 in the right arm and 46.11 ± 11.8 in the left arm), even surpassing the elite group, probably due to the combined practice of sport climbing and bouldering. Differences in muscle quality between climbers and non-climbers were mainly attributed to maximum strength, as there were no significant differences in forearm lean mass. Our findings indicate that (1) non-climbing women have higher MQ in finger flexors than men; (2) there are no significant differences in MQ between men and women who climb regularly; and (3) advanced climbers show higher MQ compared to elite climbers. In summary, climbers exhibit greater muscular quality in finger flexors compared to non-climbers. This study underscores the importance of MQ as a distinguishing metric in climbing and suggests its utility for tailoring training programs.

**Keywords:** forearm strength; climbing; body composition; finger strength; performance evaluation

**Funding:** This research was funded by Ministerio de Ciencia e Innovación, grant number PID2021125354OB-C21. AGS is a beneficiary of the Catalina Ruiz training grant program for researchers from the Consejería de Economía, Conocimiento y Empleo, as well as the Fondo Social Europeo.

**References**
  1. Baláš, J., Pecha, O., Martin, A. J., & Cochrane, D. Hand–arm strength and endurance as predictors of climbing performance. *European Journal of Sport Science*. **2012**;12(1), 16–25. https://doi.org/10.1080/17461391.2010.546431
  2. Saul D, Steinmetz G, Lehmann W, Schilling AF. Determinants for success in climbing: A systematic review. *J Exerc Sci Fit.* **2019**;17(3):91–100. doi:10.1016/j.jesf.2019.04.00
  3. Langer K, Simon C, Wiemeyer J. Physical performance testing in climbing-A systematic review. *Front Sports Act Living*. **2023**;5:1130812. Published 2023 May 9. doi:10.3389/fspor.2023.1130812
  4. Naimo MA, Varanoske AN, Hughes JM, Pasiakos SM. Skeletal Muscle Quality: A Biomarker for Assessing Physical Performance Capabilities in Young Populations. *Front Physiol*. **2021**;12:706699. Published 2021 Aug 5. doi:10.3389/fphys.2021.706699
  5. Baláš, J., Pecha, O., Martin, A. J., & Cochrane, D. Hand–arm strength and endurance as predictors of climbing performance. *European Journal of Sport Science*. **2012**;12(1), 16–25. https://doi.org/10.1080/17461391.2010.546431

### 2.30. Changes in Hip Adductor Strength in Youth Football Players Following Matches

Maziar J Hamad ^1,2,^*, Konstantinos Spyrou ^1,2^ and Pedro E Alcaraz ^1,2^

^1^  UCAM Research Centre for High Performance Sport, Murcia, Spain
^2^  Faculty of Sport, Catholic University of Murcia (UCAM), Murcia, Spain

* Correspondence: mjabarha@ucam.edu

**Abstract:** In football players, groin injuries carry a high injury burden (incidence and severity). Adductor injuries are the most common hip/groin injuries, and weak adductor strength is considered a modifiable injury risk factor. Adductor strength has been shown to decrease the week before and the week during the onset of pain. The purpose of this study was to investigate the changes to adductor strength in youth football players following official football matches. Maximum unilateral isometric hip adductor strength and pain scores (scale: 0–10) in healthy U-19 UCAM football players were measured across two official match weeks at pre-match (Pre), post-match (Post), and 24 h post-match (+24) (match 1, N = 10; match 2, N = 9). Adductor strength was measured using a handheld dynamometer (Kinvent). Only players who played ≥ 60 min were included. Repeat measures ANOVA showed a significant effect of time on hip adductor strength Pre to Post (Cohen’s d_z_ match 1, −0.75, match 2, −1.75, *p* < 0.05) and Post to +24 (Cohen’s d_z_ match 1, 0.82, match 2, 2.30, *p* < 0.05). The +24 strength was not statistically different from the Pre strength. No effects of limb or time*limb interactions were observed. In this group of youth football players, max isometric hip adductor strength seemed to decrease by between 10 and 20% post-match but then recover within 24 h. Hip and groin pain did not show consistent significant trends, but moderate-to-high pain scores (3–10) were prevalent (90–100%) among match-day players. In conclusion, football matches have varying effects and seem to decrease groin strength post-match on average by between 10 and 20% in this sample. Hip and groin pain seem to be common in this sample of youth footballers.

**Keywords:** groin; external load; injury

**Funding:** This research received no external funding.

**References**
  1. Bahr R, Clarksen B, Ekstrand J. Why we should focus on burden of injuries and illnesses, not just their incidence. *Br J Sports Med.* **2018**; 52: 1018-21.
  2. Crow JF, Pearce AJ, Veale JP, VanderWesthuizen D, Coburn PT, Pizzari T. Hip adductor muscle strength is reduced preceding and during the onset of groin pain in elite junior Australian football players. *J Sci Med Sport*. **2010**;13:202
  3. Thorborg K, Branci S, Nielsen MP, Langelund MT, Hölmich P. Copenhagen five-second squeeze: a valid indicator of sports-related hip and groin function. *Br J Sports Med*

### 2.31. Determining Reliability of Jump Performance Metrics in University Rowers Using Force Plate Analysis

Edmond Hartery ^1,^*, Thomas Comyns ^1,2^ and Frank Nugent ^1,2^

^1^  Department of Physical Education and Sport Science, University of Limerick, Limerick V94 T9PX, Ireland
^2^  Sport and Human Performance Research Centre, University of Limerick, V94 T9PX Limerick, Ireland

* Correspondence: edmond.hartery@ul.ie

**Abstract:** Assessing lower-body force and power generation is critical for performance assessment in rowing. The lower body contributes over 45% of the propulsive force during the rowing stroke [3]. Jump training is commonly used to develop force and power characteristics [4]. This study aims to evaluate the reliability and usefulness of lower-body jump performance in rowers. Twenty-six university rowers (14 male, 12 female), including 12 who represented Ireland at a junior or senior level, participated. Participants performed three trials of the squat jump (SJ), countermovement jump (CMJ), and a 5-maximal rebound jump test (RJT) in a randomized order. Participants rested for one minute between trials and three minutes between each jump protocol. Trials were conducted using force plates (Force Decks, Vald Performance) sampling at 1000 Hz. Reliability statistics were calculated using the [2] spreadsheet, assessing reliability via intraclass correlation coefficient (ICC) and coefficient of variation (CV%). Acceptable reliability was determined as an ICC ≥ 0.70 and a CV ≤ 15% [1]. Usefulness was determined by comparing typical error (TE) to the smallest worthwhile change (SWC). The SJ showed high reliability for jump height (JH) (ICC = 0.96, CV = 6.2%), mean force (Fmean) (ICC = 0.95, CV = 3.4%), and mean power (Pmean) (ICC = 0.89, CV = 9.6%). The CMJ exhibited excellent reliability for all metrics, including JH (ICC = 0.98, CV = 3.2%), Fmean (ICC = 0.99, CV = 2.1%), Pmean (ICC = 0.99, CV = 3.0%), and reactive strength index (RSI) (ICC = 0.94, CV = 6.7%). The RJT demonstrated moderate to high reliability based on JH (ICC = 0.80, CV = 9.9%), Fmean (ICC = 0.93, CV = 5.2%), Pmean (ICC = 0.91, CV = 7.7%), RSI (ICC = 0.79, CV = 12.1%), and active stiffness (ICC = 0.79, CV = 13.1%). Only CMJ performance metrics consistently rated as “good” in detecting the SWC in performance: JH (SWC: 1.40 > TE: 0.95), Fmean (SWC: 48.54 > TE: 28.15), and Pmean (SWC: 96.64 > TE: 55.18). The CMJ test demonstrated the highest reliability across all measured metrics, making it the most reliable protocol for assessing jump performance in rowers. The SJ also demonstrated high reliability for most metrics, whereas the RJT displayed higher variability and measurement error, particularly in RSI and active stiffness. These findings support using CMJ and SJ for reliable jump performance assessment in rowers.

**Keywords:** reliability; usefulness; rowing; jump performance; lower-body assessment

**Funding:** This research received no external funding.

**References**
  1. Haff GG, Ruben RP, Lider J, Twine C, Cormie P. A comparison of methods for determining the rate of force development during isometric midthigh clean pulls. *J. Strength Cond. Res*. **2015**;29, 386–95.
  2. Hopkins WG. Spreadsheets for analysis of validity and reliability. *Sports Sci.* **2015**;19: 36–42.
  3. Kleshnev V. Power in Rowing. UK: *Routledge*, **2002.**
  4. Turner, A.N, Jeffreys, I. The stretch-shortening cycle: Proposed mechanisms and methods for enhancement. *Strength & Cond. J.* **2010**;32, 87–99.

### 2.32. Effectiveness of High-Intensity Interval Training on Mental Health Outcomes in Adolescents and Young Adults: A Systematic Review

Miguel Heres ^1,2^, Susana Pulgar ^1,2^, María Fernández del Valle ^3,4^ and Hugo Olmedillas ^4,^*

^1^  Faculty of Health Sciences, Universidad Europea del Atlántico, Santander, Spain; miguel.heres@uneatlantico.es (M.H.); susana.pulgar@uneatlantico.es
^2^  Asturian Research Group in Performance, Readaptation, Training, and Health (AstuRES), Oviedo, Spain
^3^  Health Research Institute of the Principality of Asturias (ISPA), Oviedo, Spain; dr.maria@fdelvalle.net (M.F.-V.)
^4^  Department of Functional Biology, University of Oviedo, Oviedo, Spain

* Correspondence: olmedillashugo@uniovi.es

**Abstract:** Sedentary behavior has been associated with an increased risk of non-communicable diseases. The prevalence of physical inactivity is increasing, along with the levels of depression and anxiety. While any form of physical activity can enhance various health indicators, High-Intensity Interval Training (HIIT) has been found to be particularly effective in improving clinical outcomes. The aim of this review was to examine the potential benefits of HIIT on the mental health of adolescents and young adults. For this systematic review, five databases (Embase, PubMed, Scopus, SPORTDiscus, and Web of Science) were scoured from their inception until July 2024. The studies considered were Randomized Controlled Trials (RCTs) involving a non-clinical population aged between 11 and 29 years. These studies were compared to those with moderate continuous training or without intervention. HIIT was considered when the protocol was performed at or above 85% of the maximum heart rate. Of the initial 2408 studies retrieved, nine met the inclusion criteria. Most HIIT interventions spanned 8–16 weeks, with one notable exception being a 12-month school intervention program conducted 2–3 times per week, each session lasting 8–20 min. HIIT reported minor improvements in psychological well-being and perceived appearance. Some studies reported moderating effects on mental health status, with stronger effects observed among those with poor mental health at baseline. While HIIT was found to increase the inflammatory response, which is associated with improved mental health, moderate-to-vigorous continuous training was deemed more feasible for enhancing positive effects and well-being. HIIT demonstrated significant improvements in health-related quality of life, but no significant changes were observed in other mental health indicators.

**Keywords:** cardiorespiratory fitness; high intensity interval training; mental health; systematic review

**Funding:** This research received no external funding.

**References**
  1. Martland R, Mondelli V, Gaughran F, Stubbs B. Can high-intensity interval training improve physical and mental health outcomes? A meta-review of 33 systematic reviews across the lifespan. *Journal of Sports Sciences*. **2020**;38(4):430–469.
  2. Amatori S, Ferri-Marini C, Gobbi E, Sisti D, Giombini G, Rombaldoni R, Rocchi MBL, Lucertini F, Federici A, Perroni F, Calcagnini G. Short High-Intensity Interval Exercise for Workplace-Based Physical Activity Interventions: A Systematic Review on Feasibility and Effectiveness. *Sports Medicine*. **2023**;53(4):887–901.

### 2.33. Eccentric Phase Velocity Determines the Load–Velocity Relationship in Squat Jump and Bench Press Throw Exercises: A Preliminary Study

**Jose Luis Hernández-Davó ^1^, Miguel López-Fernández ^2^, Pablo Asencio ^2^, Alejandro Oliver ^2^, Daniel Cerdán ^2^ and Daniel Boullosa ^3^**

^1^  Faculty of Health Sciences, Isabel I University, Burgos, Spain; jlhdez43@gmail.com
^2^  Sport Sciences Department, Miguel Hernandez University, Elche, Spain
^3^  Faculty of Physical Activity and Sports Sciences, Leon University, León, Spain

* Correspondence: jlhdez43@gmail.com

**Abstract:** Evaluation of the load–velocity relationship is a well-established method for assessing neuromuscular capacities in different populations. This evaluation can be used for athlete profiling, to prescribe velocity-based resistance training, and to predict maximum dynamic strength. However, recent studies (1,2) have highlighted several variables that influence the outcomes of this evaluation (e.g., load selection, velocity variable used, measurement device). Besides these well-known variables, instructions about eccentric phase velocity are not standardized in the literature, despite its significant influence on concentric-phase performance (3,4). Therefore, the aim of this study was to analyze the influence of two different commonly used eccentric tempos (3 s and maximal intended velocity) on the load–velocity relationship in the squat jump (SJ) and the bench press throw (BPT) exercises. After the evaluation of 1 repetition maximum (1 RM) in SJ and BP exercises and a familiarization session, six male participants (22.3 ± 1.6 years) with previous experience (>2 years) in resistance training performed a single testing session consisting of four sets of two repetitions of each exercise under two different conditions: 3 s eccentric tempo and maximal eccentric intended velocity. The loads used were 20, 40, 60, and 80% 1RM. Mean propulsive velocity (MPV) and peak velocity (PV) during the concentric phase were recorded at 1.000 Hz using a linear position transducer (T-Force System). The results showed that under the maximal eccentric velocity condition, MPV in the SJ (8.9–14.1%) and the BPT (8.6–32.8%) exercises were significantly higher than under the 3 s eccentric condition. For PV data, significantly higher values were found favoring the maximal eccentric velocity condition at 60 and 80% 1RM in the BPT (7.9–23.5%) and at 40, 60, and 80% 1RM in the SJ (5.8–6.4%). These results highlight that the load–velocity profile differs depending on the eccentric phase velocity used during testing. Furthermore, the difference in concentric performance seems to be load-dependent, with higher loads benefiting more from fast eccentric actions. Consequently, the load–velocity profiling and the neuromuscular capabilities derived from this test (e.g., V0, L0, Pmax) are often incorrectly evaluated.

**Keywords:** load–velocity profile; resistance training; testing

**Funding:** This research received no external funding.

**References**
  1. Greig L, Aspe RR, Hall A, Comfort P, Cooper K, Swinton PA. The Predictive Validity of Individualised Load-Velocity Relationships for Predicting 1RM: A Systematic Review and Individual Participant Data Meta-analysis. *Sports Med*. **2023**;53(9):1693–1708.
  2. Marston KJ, Forrest MRL, Teo SYM, Mansfield SK, Peiffer JJ, Scott BR. Load-velocity relationships and predicted maximal strength: A systematic review of the validity and reliability of current methods. *PLoS One*. **2022**;17(10):e0267937.
  3. Hernández-Davó JL, Sabido R, Omar-García M, Boullosa D. Why Should Athletes Brake Fast? Influence of Eccentric Velocity on Concentric Performance During Countermovement Jumps at Different Loads. *Int J Sports Physiol Perform*. **2024**;19(4):375–382.
  4. Mike JN, Cole N, Herrera C, VanDusseldorp T, Kravitz L, Kerksick CM. The Effects of Eccentric Contraction Duration on Muscle Strength, Power Production, Vertical Jump, and Soreness. *J Strength Cond Res*. **2017**;31(3):773–786.

### 2.34. Variations in Fitness Performance and Training Load Between Higher and Lower-Level Young Soccer Players

**Hidalgo-de Mora, J ^1^; Maia, F ^2^; Nakamura, FY ^2^, de Hoyo Lora M ^3^, Campos-Vázquez, MA ^4^ and Pareja-Blanco, F ^1,4^**

^1^  Physical Performance & Sports Research Center, Department of Sports and Computers Sciences. Universidad Pablo de Olavide, Sevilla, Spain
^2^  Research Center in Sports Sciences, Health Sciences and Human Development (CIDESD), University Institute of Maia (ISMAI), Maia, Portugal
^3^  Department of Human Motricity and Sports Performance. University of Seville, Sevilla, Spain
^4^  Faculty of Sport Sciences. Department of Sports and Computers Sciences. Universidad Pablo de Olavide, Sevilla, Spain

* Correspondence: javierhidalgodemora@gmail.com

**Abstract:** Training load (TLoad) is monitored with the aim of making evidence-based decisions on appropriate loading schemes to reduce injuries and enhance team performance (1). Several factors are associated with training effectiveness, including the season, the players’ competitive level, and the baseline level or the training load imposed (2, 3). In soccer, the relationship between TLoad and fitness performance (4) is of considerable interest. Therefore, the aim of this study was to analyze variations in fitness status throughout the season and to determine its relationships with accumulated TLoad in young soccer players at different levels of competition (elite vs. amateur). Fourteen young male soccer players belonging to two teams (elite [U19]: n = 7; amateur [U23]: n = 7) in the same Spanish First Division academy were studied using global positioning system devices (GPSports EVO; 10 Hz) over 23 consecutive weeks (20 matches). Soccer training was conducted for both teams. Players were assessed three times across the total competitive period (i.e., early-season [TEST0], mid-season [TEST1], and end-season [TEST2]) with countermovement jump test (CMJ), 20 m sprint test (T10, T10-20, and T20), V-Cut test (V-Cut), and 30–15 intermittent fitness test (VIFT). TEST0 values were selected as baseline fitness performance values, and the TLoad was calculated between tests (TLoad-1: 12 weeks; TLoad-2: 11 weeks; TLoad-total: 23 weeks). A significant team × time interaction was observed in T10, T10-20, and T20 (*p* < 0.05). Improvements in T10, T10-20, and T20, with better VIFT performance, were observed in U19 compared to U23 during TEST2. TLoad-1 was only significantly lower for the distance recorded at sprint (D sprint) in the U23 compared to the U19 groups. TLoad-2 and TLoad-total showed significantly higher values for total distance (TD), high speed running (HSR), D sprint, and number of accelerations (Acc) and decelerations (Dec) in U19 compared to U23. In the mid-season, an inverse correlation was found between changes in T10 and T20 with TLoad-1 (D Sprint; [r = −0.65; −0.68, *p* < 0.05]). A positive association was also observed between TLoad-total (D Sprint) and changes in VIFT [r = 0.54, *p* < 0.05] at the end of the season. Our results highlight the differences in TLoad between higher and lower-level soccer players, affecting fitness performance. In conclusion, the significant changes observed in youth players’ fitness performance across the season can be explained by the load imposed, emphasizing the need for the development of physical fitness plans for young players in elite soccer clubs.

**Keywords:** time–motion characteristics; physical performance; physical development; young soccer player

**Funding:** This research received no external funding.

**References**
  1. Akenhead R, Nassis GP. Training Load and Player Monitoring in High-Level Football: Current Practice and Perceptions. *International journal of sports physiology and performance*. **2016**;11(5):587–93.
  2. Caldwell BP, Peters DM. Seasonal variation in physiological fitness of a semiprofessional soccer team. *Journal of strength and conditioning research.* **2009**;23(5):1370–7.
  3. Clemente FM, Nikolaidis PT, Rosemann T, Knechtle B. Dose-Response Relationship Between External Load Variables, Body Composition, and Fitness Variables in Professional Soccer Players. *Frontiers in physiology*. **2019**;10:443.
  4. Jaspers A, Brink MS, Probst SG, Frencken WG, Helsen WF. Relationships Between Training Load Indicators and Training Outcomes in Professional Soccer. *Sports medicine*. **2017**;47(3):533–44.

### 2.35. The Athlete’s Brain in Default Mode: A Systematic Review of Methods, Measurements, and Insights (2004–2024)

Thomas G. Huyghe ^1,^*, Pedro E. Alcaraz ^2^ and Julio Calleja-González ^3^

^1^  UCAM Research Center for High Performance Sports; gthomas@ucam.edu
^2^  UCAM Research Center for High Performance Sports; palcaraz@ucam.edu
^3^  Universidad del País Vasco; julio.calleja.gonzalez@gmail.com

* Correspondence: thomashuyghe@hotmail.com

**Abstract:** Humans spend about half of their waking life thinking about something other than their immediate surroundings—often referred to as “the brain in default mode” (BDM) (1). This state can be described as a wakeful resting state, during which outwardly focused attention is reduced, while cognitive resources are devoted to spontaneous, self-generated thoughts and sensations (2). The interest in this construct has grown rapidly across a wide range of scientific disciplines and industries for several key reasons. First, BDM presents a plethora of transdiagnostic biomarkers for mental health, cognitive function, and performance (2). Second, BDM emerges spontaneously when the mind is not directly engaged in any particularly imposed task or externally imposed stimulus; hence, researchers can empirically investigate BDM through controllable settings (1, 2). Third, recent advancements in Natural Language Processing (NLP) and Machine Learning (ML) techniques allow for faster, more efficient, and more effective approaches for investigating BDM (3). However, significant methodological challenges remain due to variations in data collection and analysis techniques. Therefore, this systematic review comprehensively examined the methods, measurements, and findings of studies (published between 2004 and 2024) on athletes’ BDM measured outside their competitive environments to determine best practices. Utilizing a rigorous PRISMA-compliant search strategy across multiple databases, 21 relevant studies met our inclusion criteria. Our review highlights three dominant approaches to assessing BDM in athletes: neuroimaging (fMRI, EEG), psychometric evaluations (surveys, self-report inventories), and physiological measurements (heart rate variability, salivary cortisol). Results show that neuroimaging techniques dominated the literature on BDM in athletes; the BDM profiles in athletes differ from those of non-athletes, and attributes such as intensity of training, type of sport, and competitive level shape BDM, highlighting the subtlety of baseline athletic brain function. However, diverse study designs, heterogeneous nomenclature, small sample sizes, and inconsistent measurement protocols (i.e., bias towards retrospective reports) across studies complicated data synthesis and cross-study comparisons. This review highlights the importance of standardized methods, time and context-sensitive measurements, and longer studies to explore how BDM changes over time in athletics and what it means for long-term psychological health and athletic performance. In conclusion, BDM presents a valuable window for sports scientists and practitioners. Recent developments in NLP, ML, and integrated multimodal approaches offer hope for more precise, pragmatic, and holistic evaluations. Sports mental health researchers and prevention program designers are advised to consider BDM assessments with athlete monitoring systems to improve their baseline understanding for optimal function, well-being, and performance.

**Keywords:** default mode network; resting-state brain activity; mind wandering; day dreaming; self-generated thoughts; stream of consciousness; functional connectivity

**Funding:** This research received no external funding.

**References**
  1. Wamsley, E. J. Dreaming, waking conscious experience, and the resting brain: report of subjective experience as a tool in the cognitive neurosciences. *Frontiers in Psychology* **2013**, 4, 637.
  2. Broyd, S J.; Demanuele, C.; Debener, S.; Helps, S K.; James, C. & Sonuga-Barke, E. Default-mode brain dysfunction in mental disorders: A systematic review. *Elsevier BV* **2009**, 33, 3, 279–296.
  3. Li, H. X.; Lu, B.; Chen, X.; Li, X. Y.; Castellanos, F. X. & Yan, C. G. Exploring self-generated thoughts in a resting state with natural language processing. *Behavior research methods* **2021**, 1–19.

### 2.36. Leveraging the Power of “AI Swarms” in High Performance Sports: A Practical, Low-Code, and Open-Source Framework for Designing Multi-Agent Collaboration Systems

Thomas G. Huyghe ^1,^*, Muaz Maqbool ^2^, Pedro E. Alcaraz ^3^ and Julio Calleja-González ^4^

^1^  UCAM Research Center for High Performance Sports; gthomas@ucam.edu
^2^  Georgia Institute of Technology; muazmaqbool65@gmail.com
^3^  UCAM Research Center for High Performance Sports; palacaraz@ucam.edu
^4^  Universidad del País Vasco; julio.calleja.gonzalez@gmail.com

* Correspondence: thomashuyghe@hotmail.com

**Abstract:** The rapid evolution of artificial intelligence (AI) presents unprecedented opportunities to enhance decision-making processes across various domains, including high-performance sports. Recognizing the current gap between the growing complexity of AI and the limited scope of AI literacy in sports leaders, we introduce a practical, low-code, and open-source framework for designing and orchestrating multi-agent systems—often referred to as “AI swarms”—aimed at improving workflows and operations in sports organizations. The framework is tailored to sports leaders and managers, providing a step-by-step guideline for developing and implementing AI-driven solutions to support critical decisions. A comprehensive review of the literature and industry standards for the use of AI in sports informed the development of our framework; particularly, the framework adheres to international standards and regulations for AI bioethics and safe system design (1), human-in-the-loop (HITL) approaches inspired by human group dynamics (2), established evidence-based frameworks for knowledge transfer, applied research, and decision-making in sports (3, 4), and international consensus on the scope of practices for sports practitioners (e.g., International Olympic Committee). Our guidelines focus on the utilization of an open-source Graphical User Interface (GUI) (i.e., Tribe AI), translation of Standardized Operating Procedures (SOPs) into prompt sequences, and explanation of the process in technical and layman terms to facilitate the comprehension, construction, and coordination of multi-agent systems in a low-code environment. The practical application of our framework was demonstrated through a sample use case scenario (i.e., determining the daily training load prescription of an athlete), which involves both “slow” and “fast” decisions (2). This use case exemplifies the orchestration of AI swarms to process diverse data inputs and provide actionable insights. Consequently, our framework simplified the development and deployment of multi-agent systems, allowing sports managers to adopt AI without extensive programming knowledge. Therefore, this framework provides a foundation for integrating AI swarms into sports management, promoting practical, low-code solutions that follow scientific methods and adhere to ethical standards and safety regulations. Future studies will be focused on refining our framework through the integration of more recent ethical and regulatory standards for responsible and sustainable deployment of AI in sports; the demonstration of its actual use in different sporting entities; and the introduction of more advanced AI methods, specifically, machine learning, predictive analytics, and adaptive self-improvement and self-regulation systems, which will further enhance its critical decision-making capabilities.

**Keywords:** collaborative AI; collaborative intelligence; sports intelligence; business intelligence; multi-agent systems; agent swarms

**Funding:** This research received no external funding.

**References**
  1. Ashok, M.; Madan, R.; Joha, A.; & Sivarajah, U. Ethical framework for Artificial Intelligence and Digital technologies. *International Journal of Information Management* **2022**, 62, 102433.
  2. Mosqueira-Rey, E.; Hernández-Pereira, E.; Alonso-Ríos, D.; Bobes-Bascarán, J.; & Fernández-Leal, Á. Human-in-the-loop machine learning: a state of the art. *Artificial Intelligence Review* **2023,** 56, 3005–3054.
  3. Ward, P.; Windt, J.; & Kempton, T. Business intelligence: how sport scientists can support organization decision making in professional sport. *International journal of sports physiology and performance* **2019**, 14, 544–546.
  4. Bartlett, J. D.; & Drust, B. A framework for effective knowledge translation and performance delivery of sport scientists in professional sport. *European journal of sport science* **2021**, 21, 1579–1587.

### 2.37. Acute Effects of Isometric Conditioning Activity with Different Contraction Durations on Countermovement Jump Performance

Jakub Jarosz ^1,^*, Milosz Drozd ^1^, Dawid Gawel ^1^, Michal Wilk ^1,2^, Jonatan Helbin ^1^, Adam Zajac ^1^, and Michal Krzysztofik ^1,2^

^1^  Institute of Sport Sciences, The Jerzy Kukuczka Academy of Physical Education, Katowice, Poland; j.jarosz@awf.katowice.pl, m.drozd@awf.katowice.pl, d.gawel@awf.katowice.pl, m.wilk@awf.katowice.pl, a.zajac@awf.katowice.pl, m.krzysztofik@awf.katowice.pl
^2^  Department of Sports Games, Faculty of Physical Education and Sport, Charles University, Prague, Czech Republic

* Correspondence: author: Jakub Jarosz, Department of Sports Training, The Jerzy Kukuczka Academy of Physical Education, ul Mikołowska 72a, 40-065 Katowice Poland; j.jarosz@awf.katowice.pl

**Abstract:** Isometric muscle contractions are widely used in research and training due to their effectiveness in eliciting acute post-activation performance enhancement (PAPE). Common modifications in isometric conditioning activities involve manipulating variables such as method, intensity, and contraction duration. However, the distribution of single repetition duration in isometric contractions to elicit the PAPE effect is less explored. This study aimed to compare the effects of isometric conditioning activities (CAs) with equal total duration but different repetition distributions on force production during the CAs and subsequent countermovement jump (CMJ) performance. Fifteen males participated in this study (age: 22.1 ± 2.4 years; body mass: 85.1 ± 9.7 kg; height: 181.3 ± 6.5 cm; relative one-repetition maximum [1RM] in back squat: 1.59 kg/body mass). Participants completed four conditions, each consisting of maximum voluntary isometric contraction in the half back squat position against an immovable barbell, which differed in repetition distribution, i.e., three sets of nine repetitions lasting 1 s each (BALL), three sets of three repetitions lasting 3 s each (PIMA3), three sets of single repetitions lasting 9 s, and a control condition (CONT) without CA. A 1 min rest between sets was allowed. Approximately 3 min before CA and approximately 30 s, 3, 6, 9, and 12 min after CA, the CMJ height was assessed. Moreover, peak force production and force generated at 100 and 200 ms during each CA were evaluated using force plates. A two-way repeated measures ANOVA revealed a main effect of time on CMJ height (*p* = 0.029; ηp^2^ = 0.171) but did not show significant differences between conditions (*p* = 0.434; ηp^2^ = 0.067) or interactions (*p* = 0.648; ηp^2^ = 0.060). Post-hoc comparisons indicated a significant decrease in CMJ height at the ninth minute (Mean = −35.7 ± 5.6 cm) compared to the third minute (Mean = −36.8 ± 5.5 cm post-CA (Mean difference [MD] = −0.9 ± 0.2 cm; Cohen’s d= 0.161; pbonf = 0.048). In addition, there were no significant interactions or main effects for contraction time (CT) and concentric peak power (CPP) of the CMJ, and PF and Force100 in CA. However, a main effect of condition was demonstrated on Force200 (*p* < 0.001; ηp^2^ = 0.686). Post-hoc comparisons revealed higher Force200 values in BALL (MD = 549 ± 137 N; Cohen’s d = 1.049; pconf < 0.001) and PIMA3 (MD = 348 ± 112 N; Cohen’s d = 0.665; pconf = 0.002) compared to the PIMA9 condition. None of the isometric CAs used in the present study had any effect on the CMJ. However, significant differences in Force200 values in BALL and PIMA3 conditions indicate that the predetermined duration of isometric contractions affects the level of force generated during their execution.

**Keywords:** PAPE; post-activation performance enhancement; isometric strength; rate of force development

**Funding:** This research received no external funding.

**References**
  1. Balshaw, T.; Massey, GJ.; Maden-Wilkinson, TM.; Tillin, NA.; Folland, JP. Training-specific functional, neural, and hypertrophic adaptations to explosive- vs. sustained-contraction strength training. *J Appl Physiol* **2016**, *120(11)*,1364–1373.
  2. Bogdanis, GC,; Tsoukos, A,; Methenitis, S,; Selima, E,; Veligekas, P,; Terzis, G. Effects of low volume isometric leg press comples training at two knee angles on force-angle relationship and rate of force development. *Eur J Sport Sci* **2019**,*19(3)*,345–53.
  3. Lum, D.; Barbosa, T. M.; Joseph, R.; & Balasekaran, G. Effects of two isometric strength training methods on jump and sprint performances: A randomized controlled trial. *Journal of Science in Sport and Exercise* **2021**, *3*, 115–124.
  4. Tillin, NA.; Folland, JP. Maximal and explosive strength training elicit distinct neuromuscular adaptations, specific to the training stimulus. *Eur J Appl Physiol* **2014**,*114(2)*,365–374.

### 2.38. Acute Effects of Different Set Configurations with Different Speed Loss Thresholds on Countermovement Jump Performance in the Full Squat Exercise

Manuel Jesús Jiménez Roldán ^1,^*, Carlos de la Rocha Galiano ^2^, Fernando Parejo Blanco ^3^ and José Antonio Páez Maldonado ^1^

^1^  Escuela Universitaria de Osuna (Universidad de Sevilla); manueljr@euosuna.org
^2^  Universidad de Sevilla; galianodelarocha@gmail.com
^3^  Universidad Pablo de Olavides; fparbla@upo.es

* Correspondence: manueljr@euosuna.org

**Abstract:** (1) Background: Jump performance through the Countermovement Jump (CMJ) is one of the most efficient tools for quantifying fatigue via the mechanical performance of athletes (1). Therefore, CMJ height is a reliable tool for measuring both neuromuscular fatigue over the course of a season and acute fatigue after a training session. Therefore, the scientific literature has focused on studying mechanical and metabolic fatigue through strength training designs, comparing the effects of different recovery periods between repetitions or sets of repetitions (2). The objective of the present study was to analyze the effects of two traditional structures and two cluster structures on performance through jump height in a CMJ. (2) Methods: Sixteen physically active individuals completed four different strength training protocols for squats. Each protocol maintained the same relative intensity (70% 1-RM), number of sets (three), and rest time between sets (4 min). The protocols were as follows: 1) TR20: with a 20% speed loss (SL) per set; 2) TR40: with a 40% SL per set; 3) CLU20: using a cluster configuration (10 s rest between repetitions while securing the bar on the Smith machine) until a 20% SL was reached; and 4) CLU40: using a cluster configuration until a 40% SL was reached. In all protocols, the CMJ was assessed at various points: before exercise (Pre) and at 3 min, 9 min, 24 h, 48 h, and 72 h post-exercise. (3) Results: There were no significant differences for the “protocol effect,” as no significant differences were observed between the protocols, nor for the “protocol × time effect.” However, there were significant differences for the “time effect.” These significant differences were highlighted in the “Post” and “9-min-Post” measurements, where the subjects were fatigued. However, it was observed that from “24 h-Post” onwards, the subjects were not fatigued, and they had recovered fully by “48 h-Post. (4) Conclusions: The study found no significant differences in performance outcomes between different strength training protocols, indicating that both traditional and cluster configurations yield similar results. However, significant “time effects” were observed, with subjects showing marked fatigue immediately post-exercise and 9 min post-exercise. Recovery was significant by 24 h post-exercise, with full recovery achieved by 48 h post-exercise. These results emphasize the importance of incorporating sufficient recovery periods in training programs to optimize performance and prevent overtraining.

**Keywords:** CMJ; strength training; cluster training; squat

**Funding:** This research received no external funding.

**References**
  1. Moreno SM. Counter-movement Jump height as a means to monitor neuromuscular fatigue. *Systematic review. Retos.* **2020**;37:820–6.
  2. García-Ramos A, Padial P, Gregory Haff G, Arguelles-Cienfuegos J, Miguez GR, Javier CP, et al. Effect of different interrepetition rest periods on barbell velocity loss during the ballistic bench press exercise. *J Strength Cond Res*. **2015**;29(9):2388–96.

### 2.39. Acute Responses to Traditional and Cluster-Set Squat Training with Different Loss Velocities on Executive Functions

Manuel Jesús Jiménez Roldán ^1,^*, Carlos de la Rocha Galiano ^2^, Fernando Parejo Blanco ^3^ and José Antonio Páez Maldonado ^1^

^1^  Escuela Universitaria de Osuna (Universidad de Sevilla); manueljr@euosuna.org
^2^  Universidad de Sevilla; galianodelarocha@gmail.com
^3^  Universidad Pablo de Olavides; fparbla@upo.es

* Correspondence: manueljr@euosuna.org

**Abstract:** Acute strength training has been shown to improve cognitive functions such as executive function (EF) (2); however, few studies have directly compared the acute effects of EF performance on velocity-based strength training (VBST), comparing different types of training methods (Traditional [TR] vs. Cluster [CL]) and different percentages of loss velocities (20% vs. 40%). The aim of this study was to compare the acute responses of EF to different set configurations (TR vs. CL) in full squat (SQ) under different loss velocities (20% vs. 40%). Sixteen resistance-trained males performed four protocols that differed in the set configuration (TR20%, TR40%, CL20%, and CL40%). The relative intensity (70% 1RM), volume (three sets), and resting time (2 min) were equated. The Stroop test, Digit Span test, and Wisconsin card test were assessed before the test and 10 min after the end of each training program. Analysis of the results showed that there were no statistically significant differences in any of the cognitive tests (Stroop test, Digit Span test, and Wisconsin card test) between the different training conditions. Despite the variation in training style and loss of speed, participants’ executive function performance did not show significant changes post-training. These findings suggest that, in an acute context, the type of speed-based training (traditional vs. cluster) and the level of speed loss (20% vs. 40%) do not have a significant impact on executive functions as assessed by the Stroop test, Digit Span test, and Wisconsin card test. It is possible that other factors, such as the duration of training or the training status of the subjects, play a more important role in the modulation of cognitive functions after physical exercise. These results call for further research to explore the conditions under which strength training may influence cognition.

**Keywords:** strength; resistance training; executive function

**Funding:** This research received no external funding

**References**
  1. Brown DMY, Bray SR. Acute effects of continuous and high-intensity interval exercise on executive function. J Appl Biobehav Res. 2018;23(3).

### 2.40. Effects of Neuromuscular Training on Stable Versus Unstable Surfaces on Unipodal Force Production in Non-Dominant Limb in Professional Soccer Players

**Sergio Jiménez-Rubio ^1^, David García-Albín ^2^ and Christel García Ortiz ^1^**

^1^  Universidad Rey Juan Carlos; sergio.jimenez.rubio@urjc.es
^2^  Universidad de Castilla-La Mancha; davidgarcia129@alu.uclm.es

* Correspondence: sergio.jimenez.rubio@urjc.es

**Abstract:** The use of unstable surfaces has become increasingly important in training programs aimed at improving athletic performance and facilitating injury rehabilitation. However, the literature associating the favorable effects of training on stable and unstable surfaces with athletic performance parameters is considerably limited. The aim of this study was to evaluate the effects of a 10-week training program on unstable surfaces compared to a training program on stable surfaces on force production. Twenty-seven professional football players (age: 26 ± 2.06 years) were randomly assigned to training on either the stable surface group (SSG; n = 14) or the unstable surface group (USG; n = 13). Ankle dorsiflexion (Ndom_DFT), one-legged distance jump (Ndom_SLH), one-legged countermovement jump (Ndom_SLCMJ), lateral jump (Ndom_SH), and Y-balance (YBT) tests were performed before and after the 10-week training period on the non-dominant limb, which was used to generate force in the unipodal vertical jump. Two-way ANOVA revealed significant improvements in the SSG group for variables NDomDFT, NDomYBT, LSIYBT, SLCMJNDom, and SLHopNdom (*p* = 0.001 to *p* = 0.017). No significant improvements were observed in the tests performed in the USG group (*p* = 0.175 to *p* = 0.939). A significant interaction was observed in the USG group (*p* = 0.175 to *p* = 0.939). A significant interaction between groups and measures was observed for all tests (*p* = 0.001 to *p* = 0.015). These results suggest that a 10-week training program conducted with tasks requiring force production and monopodal stability on stable surfaces produces significant improvements in several performance parameters in professional football players. However, training on unstable surfaces did not produce significant changes in the tests evaluated.

**Keywords:** return to play; rate of force development; performance; stability; neuromuscular control

**Funding:** This research received no external funding.

**References**
  1. Behm DG, Muehlbauer T, Kibele A, Granacher U. Effects of Strength Training Using Unstable Surfaces on Strength, Power and Balance Performance Across the Lifespan: A Systematic Review and Meta-analysis. *Sports Med*. **2015**;45(12):1645–69.
  2. López-de-Celis, C., Sánchez-Alfonso, N., Rodríguez-Sanz, J., Romaní-Sánchez, S., Labata-Lezaun, N., Canet-Vintró, M., … & P rez-Bellmunt, A. Quadriceps and gluteus medius activity during stable and unstable loading exercises in athletes. A cross-sectional study. *Journal of Orthopaedic Research*, **2024**;*42*(2), 317–325.
  3. Mirshams Shahshahani P, Ashton-Miller JA. On the importance of the hip abductors during a clinical one legged balance test: A theoretical study. *PLOS ONE*. **2020**;15(11): e0242454.

### 2.41. Artificial Intelligence Predictive Methods for Load–Injury Relationships in Team Sports: A Systematic Review

Lucas Gabriel Liofla ^1,^*, Francisco Javier Nuñez ^2^ and Luis Manuel Martínez-Aranda ^3,4^

^1^  Universidad Pablo de Olavide, Seville, Spain; lglio@alu.upo.es
^2^  Universidad Pablo de Olavide, Physical Performance & Sports Research, Seville, Spain; nunsan@upo.es
^3^  Physical and Sports Performance Research Centre, Faculty of Sports Sciences, Pablo de Olavide University, 41013 Seville, Spain; lmmarara@upo.es
^4^  SEJ-680: Science-Based Training (SBT) Research Group, Pablo de Olavide University, 41013 Seville, Spain

* Correspondence: liottalg@gmail.com; Tel.: +5493417183959

**Abstract:** Team sports are demanding, and load fluctuation can be divided into external load and internal load. Load monitoring is essential, and with the help of technology and artificial intelligence predictive methods, it is possible to establish load–injury relationships. The objective of this systematic review was to identify which artificial intelligence predictive methods are used in team sports to establish the load–injury relationship and identify the load variables that are monitored and the quantification methods used. Using the PRISMA guidelines, a systematic search was carried out in PUBMED, WoS, and Scopus, covering articles published from 2010 to April 30, 2024. Methodological evaluation was performed using a scale with specific criteria (1). A total of 1836 articles were obtained, of which 38 were included in the final review. Multiple methods and sub-methods were identified; however, the two most widely used to establish a predictive load–injury relationship were logistic regression and generalized estimating equations. Supervised methods were always used and rarely combined with unsupervised methods, while external load measures were always considered; however, the same was not true for internal load measures. Multiple studies that included external load measurements obtained data using global navigation satellite systems combined with triaxial sensors, and the most commonly used measures for external load were total time, total distance, and high-speed running. For internal load, the most commonly used measure was the session Rating of Perceived Exertion (sRPE). The same variables were used to predict injuries. Additionally, using different quantification methods, such as Acute/Chronic Workload Ratio (ACWR), weekly load, arbitrary units, accumulated week load, and changes in load from the previous week to the current week in different time periods, was appropriate in order not to leave out relationships between loading patterns and injury. In conclusion, quantification values and metrics are adjusted for each sample, each sport, and each subject, and artificial intelligence predictive methods, especially logistic regression and generalized estimating equations, are effective in establishing load–injury relationships in team sports, emphasizing the importance of monitoring both external and internal loads.

**Keywords:** training load; machine learning; injury prevention; athlete monitoring

**Funding:** This research received no external funding.

**References**
  1. Claudino JG, Capanema D de O, de Souza TV, Serrão JC, Machado Pereira AC, Nassis GP. Current Approaches to the Use of Artificial Intelligence for Injury Risk Assessment and Performance Prediction in Team Sports: a Systematic Review. *Sport Med*. **2019**;5(1):28.
  2. Vanrenterghem J. Training Load Monitoring in Team Sports : A Novel Framework Separating Physiological and Biomechanical Load-Adaptation Pathways. *Sport Med*. ***2017***;
  3. Soligard T, Schwellnus M, Alonso JM, Bahr R, Clarsen B, Dijkstra HP, et al. How much is too much? (Part 1) International Olympic Committee consensus statement on load in sport and risk of injury. *Br J Sports Med*. **2016**;50(17):1030–41.

### 2.42. Low Strength Reflects Poorer Functional Capacity and Elevated Inflammation in Maintenance Hemodialysis Patients

**Lorena Lopes ^1^ and João Felipe Mota ^2^**

^1^  Unifimes, Centro Universitário de Mineiros, Brazil; lopes.lorenacc@gmail.com
^2^  School of Nutrition, Federal University of Goias, Brazil; Jfemota@gmail.com

**Abstract:** Handgrip strength (HGS) is a simple, non-invasive measure of muscle function that is widely used as an indicator of overall muscle strength and physical performance. Low HGS has been associated with adverse outcomes in various populations, including increased mortality, morbidity, and reduced quality of life. In patients undergoing maintenance hemodialysis (MHD), muscle wasting and functional impairment are prevalent, often exacerbated by chronic inflammation and metabolic disturbances. This study aimed to evaluate the association between low HGS and body composition, functional capacity, muscle quality, and inflammatory markers in MHD patients. This cross-sectional study included patients undergoing MHD. Handgrip strength was measured using a hydraulic dynamometer on the upper limb without a fistula. Patients were stratified into low or adequate HGS groups based on population-specific cutoff points. Body composition was assessed using dual-energy X-ray absorptiometry (DXA). Functional capacity was evaluated using the Short Physical Performance Battery (SPPB) and timed up and go (TUG) tests. Serum levels of creatinine, interleukin-6 (IL-6), interleukin-10 (IL-10), tumor necrosis factor-alpha (TNF-α), and ultra-sensitive C-reactive protein (us-CRP) were measured before dialysis sessions. The study included 67 patients, 58.2% of whom were male, with a mean age of 54.1 ± 11.7 years. Patients with low HGS exhibited significantly worse functional capacity compared to those with adequate HGS, as evidenced by the TUG test results (10.7 ± 1.0 vs. 8.5 ± 0.8 s, *p* < 0.001). Inflammatory markers were also elevated in the low HGS group; IL-6 levels were 2.7 ± 0.3 pg/mL compared to 1.9 pg/mL in the adequate HGS group (*p* = 0.03), and us-CRP levels were 14.8 ± 3.0 mg/L versus 4.7 ± 1.9 mg/L (*p* = 0.03). Multiple linear regression analysis revealed that appendicular lean mass, TUG, us-CRP, age, and sex were significant predictors of HGS. Low handgrip strength in patients on maintenance hemodialysis is associated with poorer functional capacity and higher levels of inflammation. The findings suggest that HGS is a useful clinical marker for identifying patients at higher risk of functional decline and increased inflammatory burden. Regular assessment of HGS could be beneficial in the clinical management of MHD patients, aiding in the early identification of those who may require targeted interventions to improve functional capacity and reduce inflammation

**Keywords:** handgrip strength; maintenance hemodialysis; functional capacity; inflammatory markers; muscle wasting

**Funding:** This research received no external funding.

**References**
  1. Klausen HH, Petersen J, Lindhardt T, Andersen O. How inflammation underlies physical and organ function in acutely admitted older medical patients. *Mech Ageing Dev*. **2017**;164:41–48.
  2. Bielemann RM, Gigante DP, Horta BL, Barros FC. Birth weight, intrauterine growth restriction and nutritional status in childhood in relation to grip strength in adults: from the 1982 Pelotas (Brazil) birth cohort. *Nutrition*. **2016**;32(2):228–35.
  3. Vogt BP, Borges MCC, Goés CR, Caramori JCT, Pecoits-Filho R. Handgrip strength is an independent predictor of all-cause mortality in maintenance dialysis patients. *Clin Nutr*. **2016**;35(6):1429–33.

### 2.43. Comparison of Strength Loss Between the Special Judo Fitness Test and Simulated Judo Combat: A Pilot Study

Adrián Mañas-París ^1,^^†^, Luis Manuel Martínez-Aranda ^1,2,^*^,^^†^, Sonia Montero-González ^1,3^ and Jorge Ramírez-Lechuga ^1^

^1^  Faculty of Sports Sciences, Pablo de Olavide University, Seville, Spain; adrianmanasparis@gmail.com; lmmarara@upo.es; jrlechuga@upo.es
^2^  Physical and Sports Performance Research Centre, SEJ-680: Science-Based Training (SBT) research group, Pablo de Olavide University, Seville, Spain
^3^  UAX Rafa Nadal School of Sport, Physical Activity and Sports Science Degree, Alfonso X El Sabio University, Madrid, Spain; smontero@uax.es

* Correspondence: lmmarara@upo.es
^†^  Both authors contribute equally to this work.

**Abstract:** The Special Judo Fitness Test (SJFT) is a widely validated tool for assessing the specific physical fitness of judokas, focusing on their ability to sustain strength throughout a bout. This capability is crucial for performance in both training and competitive contexts (1, 2). Prior studies suggest that the physiological demands of SJFT closely mimic those of actual judo bouts, making it a reliable indicator of competitive readiness (3, 4). The study aims to compare the strength loss experienced by judokas during the SJFT and an actual judo match, measured through various physical tests. The study involved eight male participants with significant judo experience (average age 23.2 ± 1.7 years; height 171 ± 3.2 cm; weight 72 ± 5.2 kg). A cross-sectional design was used, where each participant completed three sessions, spaced at least 72 h apart. The first session established baseline measurements for vertical jump (CMJ), manual dynamometry, and bench press velocity (VMP). In subsequent sessions, participants performed the SJFT and a simulated judo bout, with measurements taken pre- and post-test to assess strength loss. Significant reductions in performance were observed post-SJFT and post-bout. Specifically, the Abalakov jump (*p* ≤ 0.004) and left-hand dynamometry (*p* ≤ 0.002) showed notable declines. The CMJ, right-hand dynamometry, and VMP also showed declines, albeit with less statistical significance (*p*-values of 0.014, 0.019, and 0.010, respectively). Notably, the strength loss measured through the various tests demonstrated similar patterns between the SJFT and judo bout, validating the SJFT as a proxy for in-match performance (ICC values for reliability were high, ranging from 0.91 to 0.98). The findings support the hypothesis that the SJFT is a reliable measure of physical performance degradation in judo. The comparable results between the SJFT and judo bouts suggest that the SJFT can effectively simulate the physical demands of competition, making it a valuable tool for coaches and athletes to assess readiness and tailor training programs. Further research with larger sample sizes and diverse demographics is recommended to enhance the generalizability of these findings.
**Keywords**: judo; SJFT; strength loss; physical performance; dynamometry; vertical jump

**Funding:** This research received no external funding.

**References**
  1. Franchini, E.; Del Vecchio, F.B.; Sterkowicz, S. Energy System Contributions to the Special Judo Fitness Test. *Int. J. Sports Physiol. Perform* **2011**, *6*, 334–343.
  2. Escobar-Molina, R.; Huertas, J.; Gutierrez Garcia, C.; Franchini, E. Special Judo Fitness Test Performance of Junior and Senior Judo Athletes from the Spanish Team. In Proceedings of the Game Drama Ritual in Martial Arts and Combat Sports, Genoa, Italy, 8–10 June 2012.
  3. Sterkowicz-Przybycień, K.; Fukuda, D.H.; Franchini, E. Meta-Analysis to Determine Normative Values for the Special Judo Fitness Test in Male Athletes: 20+ Years of Sport-Specific Data and the Lasting Legacy of Stanisław Sterkowicz. *Sports* **2019**, *7*, 194.
  4. González-Badillo, J.J.; Sánchez-Medina, L. Movement Velocity as a Measure of Loading Intensity in Resistance Training. *Int. J. Sports Med* **2010**, *31*, 347–352.

### 2.44. Determining Sex-Related Differences in the Passive Stiffness of Hamstrings in Highly Trained Football Players Using Ultrasound-Based Shear Wave Elastography

Antonio Martínez-Serrano ^1,3,4,^*, Régis Radaelli ^2^, Tomás T. Freitas ^1,3,4,5^, Pedro E. Alcaraz ^1,3,4^ and Sandro R. Freitas ^6^

^1^  UCAM Research Center for High Performance Sport, Catholic University of Murcia, Murcia, Spain; amartinez30@ucam.edu, tfreitas@ucam.edu, palcaraz@ucam.edu
^2^  Centro de Investigação Interdisciplinar Egas Moniz; (CiiEM), Egas Moniz—Cooperativa de Ensino Superior, Monte da Caparica, Portugal; rradaelli@egasmoniz.edu.pt
^3^  Strength and Conditioning Society, Murcia, Spain
^4^  Faculty of Sport Sciences, Catholic University of Murcia, Murcia, Spain
^5^  NAR—Nucleus of High Performance in Sport, São Paulo, Brazil
^6^  Laboratório de Função Neuromuscular, Faculdade de Motricidade Humana, Universidade de Lisboa, Estrada da Costa, Cruz Quebrada, Dafundo, Lisboa, Portugal; pt.sandrofreitas@gmail.com

* Correspondence: amartinez30@ucam.edu; Tel.: +34-968278566

**Abstract:** Passive muscle stiffness has been quantified using ultrasound-based shear wave elastography (SWE), providing insights into the muscles’ mechanical properties and their impact on athletic performance [1]. Recent findings indicate a negative correlation between the passive stiffness of the vastus lateralis muscle and sprint times [2]. This relationship is particularly relevant in football, where sprinting is a fundamental action and plays a critical role in hamstring injuries [3]. Additionally, male football players experience a higher rate of muscle–tendon injuries, while female players are more prone to ligament or joint injuries [4]. Given these observations, it would be interesting to investigate whether male footballers exhibit different characteristics of passive stiffness of hamstrings compared to female players, and if present, could potentially explain the sex disparity in injury patterns. Therefore, this study aimed to compare the passive stiffness of the biceps femoris long head (BFlh) and semitendinosus (ST) muscles between male and female football players. The study included 60 highly trained football players (38 males and 23 females). The passive stiffness of the BFlh and ST muscles of the players’ dominant limb was measured. Players lay prone with a neutral hip position, with the knee flexed at 30°. The probes of two matching ultrasound systems were fixed in a plastic cast in the direction of the BFlh and ST fascicles. The largest region of interest of each muscle was located, and 30 s clips were taken in SWE mode while players remained completely relaxed. The mean shear modulus values of the elastogram window were used for analysis. A linear mixed model containing muscle (BFlh, ST) and sex (female, male) as factors was used to determine the differences in the passive stiffness of the hamstrings. No significant muscle effect (*p* = 0.659) or muscle x sex interaction effect (*p* = 0.913) were found. A significant effect for sex (*p* = 0.001) showed that females presented moderately higher overall passive stiffness levels than males (*p* = 0.001; ES = 0.65; mean diff. 0.86 kPa). These findings suggest that the passive stiffness of the BFlh and ST of highly trained football players is similar between the sexes. Future research should further analyze whether the mechanical properties of the hamstring muscles under active conditions differ between male and female players.

**Keywords:** biceps femoris; semitendinosus; mechanical; performance

**Funding:** This research received no external funding.

**References**
  1. Schoeppe, S.; Alley, S.; Rebar, A.; Hayman, M.; Bray, N.; Van Lippevelde, W.; Gnam, J.; Bachert, P.; Direito, A.; Vandelanotte, C. Apps to improve diet, physical activity and sedentary behaviour in children and adolescents: a review of quality, features and behaviour change techniques. Int J Behav Nutr Phys Act **2017**, *14*, 1–10.
  2. Mateo-Orcajada, A.; Abenza-Cano L.; Albaladejo-Saura, M.; Vaquero-Cristóbal, R. Mandatory after-school use of step tracker apps improves physical activity, body composition and fitness of adolescents. Educ Inf Technol **2023**, *28*, 10235–10266.
  3. Bondaronek, P.; Alkhaldi, G.; Slee, A.; Hamilton, F.; Murray, E. Quality of Publicly Available Physical Activity Apps: Review and Content Analysis. JMIR Mhealth Uhealth **2018**, *6*, e53.

### 2.45. Relationship Between Stress Levels and the Hormonal Responses of Cortisol and Testosterone That Affect the Sporting Performance of a Professional Women’s Basketball Team

Álvaro Miguel-Ortega ^1,2,^*, Julio Calleja-González ^3,4^ and Juan Mielgo-Ayuso

^1^  Faculty of Education, Alfonso X the Wise University (UAX), Madrid 28691, Spain. amiguort@uax.es
^2^  International Doctoral School. University of Murcia (UM), Murcia 30003, Spain. a.miguelortega@um.es
^3^  Physical Education and Sport Department, Faculty of Education and Sport, University of the Basque Coutry 8 (UPV/EHU), Vitoria 01007, Spain. julio.calleja.gonzalez@gmail.com
^4^  Faculty of Kinesiology, University of Zagreb, Zagreb 10110, Croatia. julio.calleja.gonzalez@gmail.com
^5^  Faculty of Health Sciences, University of Burgos (UBU), Burgos 09001, Spain. jfmielgo@ubu.es

* Correspondence: miguel.ortega.alvaro@gmail.com

**Abstract:** The testosterone/cortisol ratio refers to the balance between cortisol and testosterone. Their levels usually increase or decrease together. This study analyzes how hormone balance affects stress in elite women’s basketball. The objective was to analyze the fluctuations in testosterone/cortisol levels and their ratio over a 16-week period and explore their connection to athletic performance. The participants had an average height of 177.6 ± 6.4 cm, were 26.0 ± 5.9 years old, had played for 14.7 ± 2.9 years, and had spent an average of 5.0 ± 1.2 years at the elite level. The testosterone/cortisol index was measured at time 1: 4.0 ± 2.4, and time 2: 5.1 ± 4.3 (n = 12). Evaluations were carried out at two time points during the competitive schedule. The first assessment (T1) was conducted in September 2018, at the start of the initial training phase, before the season began. The second assessment (T2) was conducted in January 2019, during the thirteenth week of the inaugural training phase of the second competitive cycle. Over 16 weeks of competition, participants gave blood samples for analysis (09.00 a.m. fasting) to assess various biochemical parameters, including hormone levels. In addition, their athletic performance was assessed using the following tests: jumping (SJ, CMJ, ABK, DJ); throwing test with a medicine ball (3 kg); Illinois COD agility test; sprint repeat ability with change of direction; 20 m speed test without change of direction; and Yo-yo intermittent endurance test IET (II). Eating prior to blood draws and performance testing was not monitored, and the women’s menstrual cycle was considered in sample collection. The Shapiro–Wilk normality (<30) test was employed to determine whether the variables were normally distributed before repeated or paired analyses were conducted (3) Results: The main alterations observed were an increase in T levels (1.687%) and a decrease in C levels (-7.634%) between time points, with an improvement (26.366%) in the T:C ratio. Improvements were also observed in some of the tests conducted, such as jumping (SJ: 11.5%; CMJ: 10.5%; DJ:13.0%), upper body strength (MBT: 5.4%), translation ability (20 m: −1.7%), and repeated sprint ability (RSA: −2.2%), as well as in the intermittent endurance test (Yy (IET): 63.5%), with significant changes in some of the performance tests. (4) Conclusions: The T:C ratio may differ in a manner unrelated to training volume, showing some variation. These results may be attributed to the accumulation of psychophysiological stress during the season, leading to fatigue.

**Keywords:** basketball; hormones; performance; female

**Funding:** This research received no external funding

**References**
  1. Espasa-Labrador, J.; Calleja-González, J.; Montalvo, A. M.; Fort-Vanmeerhaeghe, A. External Load Monitoring in Female 40 Basketball: A Systematic Review. *J. Hum. Kinet.* **2023**, *87*, 173. https://doi.org/10.5114/JHK/166881.
  2. Grzędzicka, J.; Dąbrowska, I.; Malin, K.; Witkowska-Piłaszewicz, O. Exercise-Related Changes in the Anabolic Index (Tes- 47 tosterone Cortisol Ratio) and Serum Amyloid A Concentration in Endurance and Racehorses at Different Fitness Levels. *Front. Vet. Sci.* **2023**, *10*, 1148990. https://doi.org/10.3389/FVETS.2023.1148990. Author 1, A.;
  3. Miguel-Ortega, Á.; Fernández-Landa, J.; Calleja-González, J.; Mielgo-Ayuso, J. Stress Levels and Hormonal Coupling and Their Relationship with Sports Performance in an Elite Women’s Volleyball Team. *Appl. Sci. 2023*, *Vol. 13*, *Page 11126* **2023**, 46 *13* (20), 11126. https://doi.org/10.3390/APP132011126.
  4. Kamarauskas, P.; Lukonaitienė, I.; Kvedaras, M.; Venckūnas, T.; Conte, D. Relationships between Weekly Changes in Salivary Hormonal Responses and Load Measures during the Pre-Season Phase in Professional Male Basketball Players. *Biol. Sport* **2023**, *40* (2), 353–358. https://doi.org/10.5114/BIOLSPORT.2023.114290.

### 2.46. The Maximum Velocity of Sprint as a Reference for Monitoring Soccer Load

Rodrigo Mohedano-Rodríguez ^1,^*, Francisco Sánchez-González ^1^, José Jiménez-Iglesias ^1^, Javier Hidalgo-deMora ^2^ and Javier Riscart-López ^3^

^1^  Sport Science Department Cádiz C.F, Cádiz C.F, Spain
^2^  Department of Sports and Computing, Sport Faculty, Pablo de Olavide University, Seville, Spain
^3^  Department of Physical Education, Faculty of Education Sciences, University of Cádiz, Puerto Real, Spain

* Correspondence: rodrigomohedanor@gmail.com

**Abstract:** The aims of the study were (1) to determine the relationship between theoretical maximal velocity (Vmax) and distances covered at absolute running thresholds (above 18 and 25.2 km·h^−1^) and (2) to examine the differences in locomotion activity between absolute and individual velocity thresholds based on the Vmax (each match) of each soccer player. Ten elite young soccer players from the same Spanish First Division academy were monitored using GPS technology during ten official matches. A descriptive design was used to evaluate the Vmax achieved in each match (3) and to examine the match running demands of the players based on the %Vmax of each absolute threshold (2). The results show a significant correlation (r = 0.44–0.91) between Vmax and the distance covered at high intensity (>18 km/h). The distance covered at sprint (>25 km/h) was explained to a greater extent by Vmax (r = 0.91) (1). Lastly, it was observed that significant differences in the quantification of the external load of each player occurred when their time–motion characteristics were monitored by absolute or relative velocity thresholds. In this regard, relativizing the locomotion activity to Vmax could allow for greater accuracy of the individual physical demands of each player during the competition and/or specific training in soccer players. In conclusion, it can be suggested that Vmax determines the distances covered at high velocity by soccer players. Therefore, designing training protocols to improve the Vmax of young soccer players could be an effective strategy for achieving higher physical performance during official competitions. Furthermore, relativizing, according to Vmax, allows individualized comparisons between different players; thus, planning training and controlling the load based on relative thresholds can be a useful strategy for the control, evaluation, and programming of soccer training.

**Keywords:** vmax; threshold; high intensity; sprint; relative; absolute

**Funding:** This research received no external funding.

**References**
  1. Castillo, D.; Raya-González, J.; Manuel Clemente, F.; Yanci, J. The influence of youth soccer players’ sprint performance on the external load of different team’ games using GPS devices. *Res Sports Med*, **2020**, *28 (2)*, 194–205.
  2. Kavanagh, R.; McDaid, K.; Rhodes, D.; McDonnell, J.; Oliveira, R.; Morgans, R. An Analysis of Positional Generic and Individualized Speed Thresholds Within the Most Demanding Phases of Match Play in the English Premier League. *Int J Sports Physiol Perform*, **2023**, *19(2)*, 116–126.
  3. Silva, H.; Nakamura, F.; Loturco, I.; Ribeiro, J.; Marcelino, R. Analyzing soccer match sprint distances: A comparison of GPS-based absolute and relative thresholds. *Biol Sport*, **2024**, *41(3)*, 223–230.

### 2.47. Impact of Relative Age Effects on the Results of the Youth Categories in the Catalonia Athletics Championships

Laia Monforte-Melich ^1^, Luis Fernández-Galván ^1^, Abraham Batalla-Gavaldà and Pau Cecília-Gallego ^1,^*

^1^  University School of Health and Sport (EUSES), Rovira i Virgili University, Amposta, Spain

* Correspondence: pau.cecilia@euseste.es

**Abstract:** In most competitions, a (bi)annual age-grouping procedure is implemented for logical organization and to reduce the physical development differences between competitors (1). However, an athlete’s maturation does not necessarily correspond to their chronological age at the individual level (2). This phenomenon, known as the Relative Age Effect (RAE), refers to the advantages or disadvantages athletes may experience due to differences in physical and biological maturation within the same age category. Therefore, the RAE has been widely studied in sports performance in initiation categories (3). The aims of the present study were twofold: first, to analyze the presence and magnitude of the RAE in the Catalan regional athletics championships and, second, to determine the influence of the birth quarter on the likelihood of achieving better results. The analysis included a total of 2241 athletes of both sexes. The 2023 season championships were evaluated across the U12 to U20 categories, covering sprints and hurdles, middle-distance, long-distance, and race walking, jumps, and throws. Data were obtained from the Catalan Athletics Federation webpage. Chi-square tests (x^2^) were used to analyze associations, and the odds ratio (OR) was calculated to determine probabilities. Our results showed a significant statistical association between the position obtained and the birth quarter for both women (*p* = 0.028) and men (*p* = 0.007). In their first year in the category, those born in Q_5_ were 12.38 times more likely to win a medal in jumping events than those born in Q_8_ (*p* = 0.022). Similarly, those born in Q_5_ were 3.11 times more likely to win a medal in throwing events than those born in Q_8_ (*p* = 0.045). In the second year of the category, it was observed that those born in Q_1_ were 2.72 times more likely to win a medal in jumping events than those born in Q_4_ (*p* = 0.017). These results suggest that the RAE can have a significant impact on athletic performance, possibly due to the physical and cognitive advantages associated with relative maturity. It is important to consider these differences when designing training programs and talent selection processes, as well as the possibility of organizing categories according to other criteria, such as bio-banding (2).

**Keywords:** growth and maturation; athlete development; youth performance

**Funding:** This research received no external funding. The APC was funded by EUSES.

**References**
  1. Musch, J., & Grondin, S. Unequal competition as an impediment to personal development: A review of the relative age effect in sport. *Developmental Review*, **2001**; 21(2), 147–167. https://doi.org/https://doi.org/10.1006/drev.2000.0516
  2. Malina, R. M., Cumming, S. P., Rogol, A. D., Coelho-e-Silva, M. J., Figueiredo, A. J., Konarski, J. M., & Kozieł, S. M. (2019). Bio-banding in youth sports: Background, concept, and application. *Sports Medicine*, **2019**; 49(11), 1671–1685. https://doi.org/10.1007/s40279-019-01166-x
  3. Cobley, S., Baker, J., Wattie, N., & McKenna, J. Annual age-grouping and athlete development. *Sports Medicine*, **2009**; 39(3), 235–256. https://doi.org/10.2165/00007256-200939030-00005

### 2.48. Age-Specific Considerations in Strength and Conditioning Programs: A Narrative Review

**Bibhu Moni Singha ^1^**

^1^  Universal Fitness Training Academy, Department of Sports and Exercise Sciences, Hengerabari, Guwahati, Assam, India, bibhuuniversal@gmail.com
^2^  Universal Injury Rehabilitation Reconditioning and High Performance Clinic, Hengerabari, Guwahati, Assam, India, infobihu12@gmail.com

* Correspondence: bibhuuniversal@gmail.com; Tel.: +91-9864047046

**Abstract:** Strength and conditioning (S&C) has always played a pivotal role in enhancing performance and minimizing the risks of injuries in young and older athletes in the general and special populations. The major challenge faced by a majority of S&C practitioners is the implementation of the right principles in the design of training programs for different age groups using evidence amid the plethora of available literature. It is an undeniable fact that aging plays a crucial role in the overall fitness of an individual; this cannot be underestimated when planning a program for different age groups. This review aims to unearth the best available evidence that can be used to design training programs with optimal benefits, as numerous studies show that a lack of early exposure to sports may raise a child’s risk of overuse injuries—particularly when developing motor skills later in life—psychological problems, overtraining syndrome, and the possibility of dropping out of the sport. With the increasing number of early sports introductions and specializations in schools, the role of S&C has increased due to its proven benefit for long-term athletic development (LTAD) [1]. To maintain an ideal dose–response relationship between important training parameters to enhance performance in young athletes, S&C programs should predominantly incorporate resistance training regimens with fewer repetitions and higher intensities. Studies show that the most effective resistance training (RT) programs for maximizing strength in young athletes should be at least 23 weeks long, five sets, with 6–8 repetitions per set, and have an intensity of 80–89% of 1 RM with a rest period of 3–4 min between sets [2]. S&C training plays a vital role in slowing down aging and reducing the risk of chronic diseases that are associated with a variety of physiological changes that lead to a decrease in skeletal muscle mass, strength, and functional fitness necessary for the health and well-being of older adults. A properly designed RT program for older adults should consist of power exercises performed at higher velocities and moderate intensities (i.e., 40–60% of 1RM) during the positive phase of the lifts. More focus should be placed on multipoint movements consisting of 2–3 sets of 1–2 exercises per large muscle group at an intensity of 70–85% of 1 RM for 2–3 times per week [3].

**Keywords:** strength and conditioning; long-term athletic development; resistance training

**Funding:** This research received no external funding.

**References**
  1. Moeskops, Sylvia PhD, CSCS1; Oliver, Jon L. PhD1,2; Read, Paul J. PhD3,4,5; Cronin, John B. PhD2; Myer, Gregory D. PhD6,7,8,9; Lloyd, Rhodri S. PhD, CSCS*D, FNSCA1,2,10. Practical Strategies for Integrating Strength and Conditioning Into Early Specialization Sports. *Strength and Conditioning Journal*, **2022**; 44(1):p 34–45. DOI: 10.1519/SSC.0000000000000665.
  2. Lesinski, M., Prieske, O., & Granacher, U. Effects and dose-response relationships of resistance training on physical performance in youth athletes: a systematic review and meta-analysis. *British journal of sports medicine*, **2016**; 50(13), 781–795. https://doi.org/10.1136/bjsports-2015-095497
  3. Fragala, Maren S.; Cadore, Eduardo L.; Dorgo, Sandor; Izquierdo, Mikel; Kraemer, William J.; Peterson, Mark D.; Ryan, Eric D. Resistance Training for Older Adults: Position Statement From the National Strength and Conditioning Association. *Journal of Strength and Conditioning Research*, **2019**; 33(8):p 2019–2052. DOI: 10.1519/JSC.0000000000003230

### 2.49. Sports Profile of Children with Specific Learning Disorders

María T. Morales-Belando ^1,^*, Aarón Manzanares ^1^, Noelia González-Gálvez ^1^, Domenico Cherubini ^1^, Gabriele Cordovani ^2^, Giuseppe Zanzurino ^2^ and Fernanda Borges ^1^

^1^  Universidad Católica de Murcia; mdtmorales@ucam.edu; amanzanares@ucam.edu; ngonzalez@ucam.edu; dcherubini@ucam.edu; bsfernanda@ucam.edu
^2^  AID—Italian Dyslexia Association, gabri.cordovani@gmail.com, zanzurinogiuseppe@gmail.com

* Correspondence: mdtmorales@ucam.edu

**Abstract:** This study sought to understand the profile of adolescents with specific learning disorders (SLDs) who participate in sports activities. A total of 237 participants were surveyed, with the majority being of Italian nationality and with varying levels of schooling. The scales used were the Coach Support Scale (COS-CY), Sport Impact Scale (SIS C-Y), and Rosenberg Self-Esteem Scale (RSES). The analyses conducted included descriptive statistics, analysis of reliability, Gaussian distribution, and analysis of variance (ANOVA). The study found that approximately 60% of participants were diagnosed with an SLD before the age of 10, and only 66% engaged in sports, with soccer, swimming, volleyball, basketball, and gymnastics being the most popular disciplines. As sports participation increased, participants reported higher levels of personal and socio-relational well-being, reduced school agitation, and decreased use of smartphones and video games. However, nearly half of the participants experienced difficulties related to their SLDs during sports practice, such as motor coordination and memory issues. The study also revealed a lack of support for children with SLDs from coaches, with many participants choosing not to disclose their condition due to fear of judgment or believing it would not make a difference. Overall, the findings underscore the importance of creating a supportive and inclusive environment within sports teams to encourage children with SLDs to feel comfortable expressing themselves. This study sheds light on the challenges and benefits associated with sports participation for individuals with SLDs, highlighting the need for increased awareness and support within the sports community.

**Keywords:** dyslexia; sports practice; schoolchildren; context

**Funding:** This research was funded by the European Union, via the project Sports activities for people with specific learning disorders (SASLED), grant number 101089447.

**References**
  1. Lorusso, M. L.; Parini, B., & Bakker, D. *Hemispheric Specialization and Dislexya*. World Dyslexia Forum. Unesco. 2010.
  2. Molisso, V.; Tafuri, D. Sport as a compensatory and educational element in specific learning disorders. *Formazione & Insegnamento* **2022**, *20*(1 Suppl.), 230–239. https://doi.org/10.7346/-feis-XX-01-22_20
  3. Tressoldi, P.; Vio, C. Confronto di efficacia tra trattamenti per la lettura in soggetti dislessici. *Psicologia Clinica dello sviluppo* **2003**, *7*(3), 483.
  4. Venâncio, P. E. M.; El Jaliss, B. E.; Teixeira Júnior, J.; Teixeira, C.G.O. *Psicomotricidad aplicada a niños con dislexia. Cuadernos de Educación y Desarrollo* **2023**, *15*(10), 10409–10423.

### 2.50. Impact of Relative Age and Maturation on Linear Acceleration of Soccer Academy Players in a Professional Club

Navarro Ballester Francisco Javier ^1,^*, González-Ródenas Joaquín ^2^ and Ballester Lengua Rafael ^3^

^1^  Escuela de Doctorado, Universidad Católica de Valencia “San Vicente Mártir”, Javier.nb@mail.ucv.es
^2^  Facultad de CC de la Actividad Física y el Deporte, Universidad Católica de Valencia “San Vicente Mártir”, rafael.ballester@ucv.es

* Facultad de CC de la Actividad Física y el Deporte, Universidad Rey Juan Carlos, Joaquín.gonzález@urjc.es

**Abstract:** Several studies suggest that talent selection in soccer is based on relative age and biological maturation, which affect attributes such as strength and speed that appear to be relevant to a player’s trajectory (1, 2). However, there is controversy regarding the specific advantages provided by the Relative Age Effect (RAE) and biological maturation (3). This study aims to analyze the influence of biological maturation, chronological age, and relative age on linear acceleration of young soccer players aged 13 to 16 years (M = 13.34 ± 2.1). A sample of 100 young soccer players was selected, categorized by developmental status, and divided into two groups: early and late maturers. Classification relied on anthropometric parameters and calculation of their temporal distance to Peak Height Velocity (PHV) period using the Mirwald estimation method. Acceleration ability was assessed through a 40 m sprint divided into 10 m, 20 m, and another 10 m segments, with speed recorded using photocells for accuracy. The potential influence of maturation, chronological age, and relative age on acceleration was considered. The main results showed no significant difference in average speed between early and late maturation groups in the U-13 to U-16 categories (*p* > 0.05). This suggests that while chronological age influenced acceleration (*p* < 0.05), neither biological maturation nor relative age impacted acceleration ability. This study provides insights into how biological maturation, chronological age, and relative age may influence the linear sprinting ability of young soccer players. It underscores the need to consider potential maturation and physical biases in acceleration performance during youth development.

**Keywords:** soccer; relative age effect; biological maturation; linear acceleration; youth development

**Funding:** This research was funded by the CONSELLERIA DE INNOVACIÓN, UNIVERSIDADES, CIENCIA Y SOCIEDAD DIGITAL from Comunidad Valenciana (Spain) to R.B.L. (grant number CIGE/2021/043).

**References**
  1. Höner, O., Murr, D., Larkin, P., Schreiner, R., & Leyhr, D. Nationwide Subjective and Objective Assessments of Potential Talent Predictors in Elite Youth Soccer: An Investigation of Prognostic Validity in a Prospective Study. Frontiers in Sports and Active Living, **2021**; 3. https://www.frontiersin.org/article/10.3389/fspor.2021.638227
  2. Rusdiana, A. Revisiting sports talent identification: A meta analysis. Journal of Engineering Science and Technology, **2021;** 16, 1258–1272.
  3. Cumming, S. P., Lloyd, R. S., Oliver, J., Eisenmann, J. C., & Malina, R. M. Bio-banding in Sport: Applications to Competition, Talent Identification, and Strength and Conditioning of Youth Athletes, **2017**; 39(2), 34–47. http://dx.doi.org/10.1519/SSC.0000000000000281

### 2.51. Maturity Status as a Modulator in the Assessment of Strength in Young Soccer Players

Navarro Ballester Francisco Javier ^1,^*, González-Ródenas Joaquín ^2^, Ballester Lengua Rafael ^3^

^1^  Escuela de Doctorado, Universidad Católica de Valencia “San Vicente Mártir”, Javier.nb@mail.ucv.es
^2^  Facultad de CC de la Actividad Física y del Deporte, Universidad Católica de Valencia “San Vicente Mártir”, rafael.ballester@ucv.es
^3^  Facultad de CC de la Actividad Física y del Deporte, Universidad Rey Juan Carlos, Joaquín.gonzález@urjc.es

* Correspondence: pjavier.nb@mail.ucv.es

**Abstract:** The conditional advantages conferred by the Relative Age Effect (RAE) and developmental maturation remain a subject of debate [1]. This study aims to analyze the influence of biological maturation on the physical performance of young soccer players. The research focuses on determining which strength tests are most effective in identifying differences in the level of maturational development among players in the U-13 to U-16 categories (M = 13.34 ± 2.1 y.o.). A sample of 100 soccer players was selected and classified into two groups: early and late maturers. Classification was based on evaluation of anthropometric characteristics and their temporal distance to peak height velocity (PHV) period using the Mirwald estimation method. The strength tests evaluated included manual dynamometry, which measures grip strength, and the Isometric Mid-Thigh Pull (IMTP), performed on a force platform to measure maximum isometric strength. Additionally, the countermovement jump (CMJ) and the rebound jump (CMRJ) were conducted on a force platform to assess the ability to develop high rates of force in short periods of time. Student’s *t*-test was used to analyze the obtained data after cleaning and preparing them with Python. The results revealed significant differences in the strength and jump tests associated with the players’ maturational status (*p* < 0.05). Players with more advanced maturational status exhibited superior physical performance in the evaluated tests. As players approach the peak height velocity (PHV) period, the magnitude of these differences decreases, indicating that biological maturation significantly impacts physical development in the early stages, but its influence diminishes over time. The conclusions underscore the importance of considering biological maturation when interpreting the results of physical tests in the identification and development of talent in youth soccer. Including the evaluation of biological maturation is essential to ensuring equitable development and maximizing the potential of players in the long term. The need for additional studies with larger samples is highlighted to validate these findings and gain a deeper understanding of the influence of biological maturation on the physical performance of young soccer players, thereby contributing to more accurate and integral development of the players.

**Keywords:** biological maturation; physical performance; young soccer players; strength tests; PHV; manual dynamometry; force platform.

**Funding:** This research was funded by the CONSELLERIA DE INNOVACIÓN, UNIVERSIDADES, CIENCIA Y SOCIEDAD DIGITAL from Comunidad Valenciana (Spain) to R.B.L. (grant number CIGE/2021/043).

**References**
  1. Cumming, S. P., Lloyd, R. S., Oliver, J., Eisenmann, J. C., & Malina, R. M. (2017). Bio-banding in Sport: Applications to Competition, Talent Identification, and Strength and Conditioning of Youth Athletes. 39(2), 34–47. http://dx.doi.org/10.1519/SSC.0000000000000281
  2. Höner, O., Murr, D., Larkin, P., Schreiner, R., & Leyhr, D. (2021). Nationwide Subjective and Objective Assessments of Potential Talent Predictors in Elite Youth Soccer: An Investigation of Prognostic Validity in a Prospective Study. Frontiers in Sports and Active Living, 3. https://www.frontiersin.org/article/10.3389/fspor.2021.638227
  3. Rusdiana, A. (2021). Revisiting sports talent identification: A meta analysis. Journal of Engineering Science and Technology, 16, 1258–1272.

### 2.52. Effects of High-Intensity Interval Training (HIIT) on Symptoms of Depression and Anxiety in Middle-Aged Women

Shadnaz Nouri ^1^ and Domenico Cherubini ^2,^*

^1^  Universidad Catolica de Murcia, Faculty of sport, Murcia, Spain; snouri@alu.ucam.edu
^2^  Universidad Catolica de Murcia, Faculty of sport, Murcia, Spain; dcherubini@ucam.edu

* Correspondence: dcherubini@ucam.edu

**Abstract:** Depression and anxiety are the two most common mental health concerns for people in the current society, affecting the quality of life. Depression has become a focus of public health due to its increase in society and the increase in the percentage of deaths due to this condition. Increased levels of anxiety and depressive symptoms put people not only at risk of suicidal ideation but also at risk of social and physical health problems. The present study aimed to investigate the effect of 10 months of high-intensity interval training (HIIT) on symptoms of depression and anxiety in middle-aged women. The research method employed was semi-experimental, and its design was pre–post-test. The purposeful sampling method employed included 120 women suffering from depression and anxiety, who were aged 50 to 55 years. The participants, who provided informed consent, were randomly divided into two groups (exercise and control), with 60 people in each group. The inclusion criteria included the absence of respiratory, inflammatory, cardiovascular, renal, and other chronic diseases, as well as the absence of sports activity in the last month. The Beck Depression Questionnaire (BDI-II) and the Beck Anxiety Questionnaire (BAI) were used as data collection tools. The training group was subjected to an intense interval training program for 10 months. The training included three sessions per week (three training sessions/week * 10 months, 120 sessions), with each session lasting approximately one hour. Each training session included 10 min of warming up and 10 min of performing 3–5 sets of exercises (10 min * 3–5 = 30–50 min), followed by 10 min of cooling down. Results showed that the level of depression and anxiety in the exercise group significantly decreased compared to the control group (*p* < 0.05) after both 6 and 10 months of training. Thus, high-intensity interval training can significantly improve symptoms of depression and anxiety

**Keywords:** HIIT exercise; depression; anxiety; middle-aged women

**Funding:** This research received no external funding.

**References**
  1. Cleopatra M.R, Gregory C.B, Nektarios A. M and Maria P. The Effect of Aerobic Fitness on Psychological, Attentional and Physiological Responses during a Tabata High-Intensity Interval Training Session in Healthy Young Women **2023**, Public Health 2023, 20, 1005. https://doi.org/10.3390/ijerph20021005.
  2. Fabiano, Nicholas. Exercise as First-Line Therapy in Depression **2023**, University of Ottawa Journal of Medicine. 12. 10.18192/uojm.v12iS1.6569.
  3. Zschucke E, Gaudlitz K, Ströhle A. Exercise and Physical Activity in Mental Disorders: Clinical and Experimental Evidence **2013,** Journal of preventive medicine and public health 46 Suppl 1(Suppl 1), S12–S21. https://doi.org/10.3961/jpmph.2013.46.S.S12

### 2.53. Performance of Male and Female CrossFitters in Three-Load Clean and Jerk Workouts and Acute Effects on Shoulder Strength

Alejandro Oliver-López ^1,^*, Tom Brandt ^2^, Annette Schmidt ^2^, Pablo Asencio ^1^, Fernando García-Aguilar ^1^ and Rafael Sabido ^1^

^1^  Sport Sciences Department, Miguel Hernandez University, 03202 Elche, Spain; aoliver@umh.es (A.O.-L.); rsabido@umh.es (R.S); pasencio@umh.es (P.A.); fernando.garciaa@umh.es (F.G.-A.)
^2^  Institut für Sportwissenschaft, Fakultät für Humanwissenschaften, Universität der Bundeswehr München, Werner-Heisenberg-Weg 39, 85577 Neubiberg, Germany; tom.brandt@unibw.de (T.B.); annette.schmidt@unibw.de (A.S.)

* Correspondence: aoliver@umh.es; Tel.: +34-648169593

**Abstract:** CrossFit (CF) is a high-intensity fitness training system emphasizing multi-joint functional movements and high muscle recruitment and combines exercises from various sports (1,2). Weightlifting exercises such as Clean and Jerk (CJ) are frequently used in CF. Unlike traditional weightlifting, CF workouts usually aim to perform a high number of repetitions or to complete movements as fast as possible, performing a lot of work in a short time. While weightlifting typically uses loads above 80% of 1RM to maximize power and performance (3), CF training combines strength and endurance and may require different loads according to the workout aim. However, multiple repetitions of overhead movements without rest can lead to neuromuscular fatigue, reducing performance and increasing injury risk, particularly in the shoulders of CF athletes (4). This study aimed to determine the optimal load to maximize the total weight lifted in a 5 min CJ workout by male and female CF participants and to identify which load reduces shoulder isometric strength the most. Ten participants (five male, five female) performed three 5 min workouts at 40%, 60%, and 80% of their CJ 1RM, with 72 h of rest between sessions. A prior session established their CJ 1RM. Repetitions and total load (kg * repetitions) were recorded, and maximal isometric shoulder strength was measured before, immediately after, and 5 min post-workout (BackCheck, Dr. Wolff, Germany). Differences between training loads (40%, 60%, and 80% of 1RM), repetitions, and total loads were analyzed using repeated measures ANOVA and a two-way analysis of variance (ANOVA with two factors: training and gender, with three and two levels, respectively). In addition, repeated measures ANOVA (pre-, post-, and ret-) with gender as a factor was also carried out to compare the data reflecting isometric shoulder strength. Statistical significance was set at *p* < 0.05. Results showed significant differences in total repetitions across the three loads (ES = 0.8, *p* < 0.001); however, no gender differences were found for the same load. Both men and women lifted significantly more total weight at 40% and 60% compared to 80% (ES: 0.6, *p* < 0.001; ES = 0.4, *p* < 0.001). The 40% load did not significantly reduce isometric shoulder strength, whereas the 60% load caused a significant decrease in shoulder strength in both genders (ES = 0.9, *p* = 0.002). The 80% load significantly decreased strength only in men (ES = 0.5, *p* < 0.04). In conclusion, for CF workouts with many repetitions, loads between 40% and 60% result in more repetitions and total load than 80% loads; additionally, the 40% load produces less neuromuscular fatigue in CF athletes’ shoulders.

**Keywords:** high-intensity functional training; functional fitness training; weightlifting; resistance

**Funding:** This research received no external funding.

**References**
  1. Schlie J, Brandt T, Schmidt A. StartXFit—Nine Months of CrossFit^®^ Intervention Enhance Cardiorespiratory Fitness and Well-Being in CrossFit Beginners. Physiologia [Internet]. 2023 Sep 26;3(4):494–509. Available from: https://www.mdpi.com/2673-9488/3/4/36
  2. Oliver-Lopez A, Garcia-Valverde A, Sabido R. Summary of the evidence on responses and adaptations derived from CrossFit training. A systematic review. Retos. 2022;46.
  3. Javier Flores F., Sedano S., Redondo J. Optimal Load And Power Spectrum During Jerk and Back Jerk in Competitive Weightlifters. J Strength Cond Res. 2017;31(3):809–16.
  4. Summitt RJ, Cotton RA, Kays AC, Slaven EJ. Shoulder Injuries in Individuals Who Participate in CrossFit Training. Sports Health. 2016;8(6):541–6.

### 2.54. Muscle Strength and Aerobic Power in Chronically Trained Master Marathon Runners

Ľudmila Oreská ^1,^*, Milan Sedliak ^1^, Genc Berisha ^1^, Michal Střelecký ^1^ and Gabriel Buzgó ^1^

^1^  Faculty of Physical Education and Sports, Bratislava, Slovakia; ludmila.oreska@uniba.sk

* Correspondence: ludmila.oreska@uniba.sk

**Abstract:** Advanced ageing typically involves a decrease in physical fitness, although master athletes who remain active can alleviate this decline. However, there are some distinctions in physical performance between master and younger athletes (1,2). This study compared muscle strength and aerobic power between master endurance runners and their age-matched counterparts, as well as young trained runners. The following groups were compared in a cross-sectional design: (1) young endurance runners (YER: n = 8, age: 27.38 ± 3.07 yrs, BW: 72.21 ± 7.06 kg); (2) master endurance runners (MER: n = 10, age 70.22 ± 3.87 yrs, BW: 71.44 ± 9.99 kg), (3) young age-matched group (YAG: n = 10, age 26.70 ± 2.31 yrs, BW: 92.87 ± 20.47 kg), and (4) elderly age-matched group (EAG: n = 10, age 71.10 ± 3.33 yrs, BW: 82.22 ± 8.28 kg). The inclusion criteria were as follows: (1) above 300 min per week of running, (2) regular participation of YER (at least 3 years) and MER (at least 25 years) in running competitions (10 km, half, and full marathons), and (3) a personal best time on the 10 km run of under 35 min for YER and under 55 min for MER in the last season. Body composition was measured using DXA. Lower limb muscle strength was tested as maximal voluntary torque of the knee isometric extension on a knee dynamometer. Aerobic power and HR peak were measured on a cycle ergometer using a maximal graded testing protocol with an incremental load between 20 and 30 W/min. The load was set individually for each subject depending on the predicted work capacity according to the reference values and the subject’s estimated fitness level (3). In terms of body composition, significant differences were observed in body fat and lean mass in all groups. According to the maximal and relative voluntary contraction of the lower limbs, the YER group was significantly stronger. The MER group was not significantly stronger than the EAG group. Testing aerobic power showed that the VO2max and HR peak were significantly higher in the YER group than in the MER, YAG, and EAG groups. Also, MER’s VO2max was significantly higher than EAG’s. In conclusion, our research shows that master endurance runners have better aerobic power and body composition than their age-matched counterparts. However, some decline in these aspects is observed due to natural ageing when compared to young endurance runners and adults.

**Keywords:** seniors; marathon runners; muscle strength; physical fitness

**Funding:** The study was funded by the INTERREG V-A Slovakia—Austria (acronym CAA, ITMS2014 + 305041x157), by the Slovak Research and Development Agency (Grant No. APVV-21-0164 and VEGA 0554/24).

**References**
  1. McKendry, J.; Breen, L.; Shad, B.J.; Greig, C.A. Muscle morphology and performance in master athletes: A systematic review and meta-analyses. *Ageing Res. Rev*. 2018, 45, 62–82
  2. McKendry, J. et al. Superior aerobic capacity and indices of skeletal muscle morphology in chronically trained master endurance athletes compared with untrained older adults.” *The Journals of Gerontology: Series A* 75.6 (2020): 1079–1088.
  3. Vajda, M.; Oreská, Ľ.; Černáčková, A.; Čupka, M.; Tirpáková, V.; Cvečka, J.; Hamar, D.; Protasi, F.; Šarabon, N.; Zampieri, S.; et al. Aging and Possible Benefits or Negatives of Lifelong Endurance Running: How Master Male Athletes Differ from Young Athletes and Elderly Sedentary? *Int. J. Environ. Res. Public Health* 2022, *19*, 13184. https://doi.org/10.3390/ijerph192013184

### 2.55. Acute Mechanical and Metabolic Responses and Time Course of Recovery from Bench-Press Training with Different Volumes: Isolating the Effect of Fatigue

Páez-Maldonado José Antonio ^1,2,^*, Ortega-Becerra Manuel ^2,3^ and Pareja-Blanco Fernando ^2,3^

^1^  University of Osuna (Centre attached to the University of Seville), Osuna, Spain
^2^  Science Based Training Research Group, Department of Sports and Computer Sciences, Universidad Pablo de Olavide, Seville, Spain
^3^  Physical Performance and Sports Research Center, Department of Sports and Computer Sciences, Universidad Pablo de Olavide, Seville, Spain

* Correspondence: joseapm@euosuna.org; Tel.: +34-673588800

**Abstract:** Resistance training (RT) is a frequently employed approach aimed at enhancing the muscle strength of athletes. The manipulation of a series of variables (e.g., exercise type, relative intensity, volume, rest interval) characterizes the type and magnitude of the stimulus and, consequently, the physiological adaptations to RT (1). In this regard, integrating short rest periods between repetitions in training sessions may mitigate fatigue accumulation and enhance performance (2). Additionally, knowledge of the time required to recover to baseline values after strength training is of great importance, as it can hinder the development of other physical, technical, or tactical components (3). Physical performance can be substantially impaired for up to 48 h post high volume, moderate intensity exercise (4). The purpose of this study was to investigate the acute impact of three different volumes of the bench press (BP) exercise on mechanical and metabolic responses, as well as the time course of recovery. Fourteen resistance-trained men performed three BP sessions consisting of three repetitions (LOW), 15 repetitions (MOD), and 24 repetitions (HIG) per session. The relative load was 70% 1RM for all sessions. To reduce fatigue during the set, a short rest interval of 10 s was introduced between repetitions. Additionally, a 20 s rest was introduced if the velocity difference between the fastest and subsequent repetitions exceeded 0.03 m·s^−1^. An additional 10 s of rest between repetitions was included for differences surpassing 0.06 m·s^−1^, and so on. Blood lactate (LAC) was measured before and after each protocol. Moreover, the BP strength performance (assessed as the lifting velocity against an absolute load) was recorded Post-exercise, 24 h-Post, and 48 h-Post. A 3 (protocols) × 2 (Pre vs. Post-exercise) repeated measures ANOVA was performed to analyze the acute metabolic responses, and an ANOVA with repeated measures (3 (protocols) × 4 (Pre vs. Post-exercise vs. 24 h-Post vs. 48 h-Post)) with Bonferroni adjustments was used to analyze the time course of recovery following each protocol. Significant “time” effects were displayed for both variables (*p* < 0.001), but no significant “protocol × time” interactions were observed for LAC (*p* = 0.36) and BP performance (*p* = 0.11). Therefore, HIG exercise achieved a similar rate of recovery as LOW and MOD exercises, despite HIG accumulating a higher training volume. Introducing short rest periods proves effective in reducing post-RT mechanical and metabolic responses while maintaining a high volume of training.

**Keywords:** rate of recovery; lactate; training volume; velocity-based training

**Funding:** This research received no external funding.

**References**
  1. Spiering, B.A.; Kraemer, W.J.; Anderson, J.M.; Armstrong, L.E.; Nindl, B.C.; Volek, J.S.; & Maresh, C.M. Resistance exercise biology: Manipulation of resistance exercise programme variables determines the responses of cellular and molecular signalling pathways. Sports Med. 2008, Vol.38, Issue 7).
  2. Jukic, I.; García, Ramos A.; Helms, E.; McGuigan M.; & Tufano, J. Acute Effects of Cluster and Rest Redistribution Set Structures on Mechanical, Metabolic, and Perceptual Fatigue During and After Resistance Training: A Systematic Review and Meta-analysis. Sports Med. 2020, 50(12), 2209–2236.
  3. Draganidis, D.; Chatzinikolaou, A.; Jamurtas, A. Z.; Carlos Barbero, J.; Tsoukas, D.; Theodorou, A.S.; Margonis, K.; Michailidis, Y.; Avloniti, A.; Theodorou, A.; Kambas, A.; & Fatouros, I. The time-frame of acute resistance exercise effects on football skill performance: the impact of exercise intensity. J. Sports Sci. 2013, 31(7), 714–722.
  4. Pareja-Blanco, F.; Rodríguez-Rosell, D.; Sánchez-Medina, L.; Ribas-Serna, J.; López-López, C.; Mora-Custodio, R., Yáñez-García, J. M.; & González-Badillo, J. J. Acute and delayed response to resistance exercise leading or not leading to muscle failure. Clin. Physiol.Funct. Imaging. 2017, 37(6), 630–639.

### 2.56. Evaluation of Maximum Strength and Power of Upper and Lower Body Muscles and Physical Activity Levels in Young Adults from Different Ethnic Groups

**D** avid Parra Romero ^1^, Paola Rubiano ^2^ and Jairo Fernández ^3,^*

^1^  Research teacher at the Faculty of Education, Bachelor of Physical Education, Minuto de Dios University Corporation UNIMINUTO, Bogotá, Colombia; davidparraromero90@gmail.com
^2^  Research teacher at the Faculty of Education, Bachelor of Physical Education, Minuto de Dios University Corporation UNIMINUTO, Bogotá, Colombia; prubiano@uniminuto.edu
^3^  Research Professor, Faculty of Education, Bachelor’s degree in Sports UPN Universidad Pedagogic Nacional de Bogotá, Colombia; jairofdz@gmail.com

* Correspondence: davidparraromero90@gmail.com

**Abstract:** This study aimed to investigate the relationship between maximum strength and power of different muscle groups, as well as physical activity levels in young adults from various ethnic groups. A total of 355 individuals aged 18 to 30 were assessed using specific tests to determine maximum isometric strength (FMII) and mean propulsive velocity (VMPS) of the lower body, as well as the maximum strength (FMPP) and peak power (PPP) of the upper body. Very strong correlations were found between PPP and FMPP (r = 0.904), strong correlations were found between FMII and PPP (r = 0.760) and between FMII and FMPP (r = 0.776), and weak correlations were found between VMPS and FMPP (r = 0.295), FMII (r = 0.256), and PPP (r = 0.336). The results indicate a significant relationship between strength and power levels within the same muscle groups, and a strong relationship between the strengths of different muscle groups. These findings suggest that both strength and power are important for overall muscle performance, and their relationship may vary depending on the muscle group and the type of physical activity. This study contributes to the understanding of how muscle strength and power are related and highlights the importance of considering both factors in physical training and health assessments. The methodology included identifying the problem, conducting tests, and interpreting the results to determine the correlation between different manifestations of strength in various muscle groups. The findings provide valuable insights into the role of muscle strength and power in physical performance and health, emphasizing the need for further research to explore these relationships in more detail. This research is significant for sports science and public health as it underscores the importance of maintaining muscle strength and power to prevent chronic diseases and improve the quality of life.

**Keywords:** isometric force; dynamic force; power peak; mean propulsive velocity

**Funding:** This research received no external funding.

**References**
  1. Brown LE, Weir JP. ASEP Procedures recommendation I: accurate assessment of muscular strength and power. J Exerc Physiol. 2001;4(3):1–21.
  2. Pareja-Blanco F, et al. Effects of velocity loss during resistance training on athletic performance, strength gains and muscle adaptations. Scand J Med Sci Sports. 2014;27(7):724–735.
  3. Cronin J, McNair PJ, Marshall RN. Force-velocity analysis of strength-training techniques and load: implications for training strategy and research. J Strength Cond Res. 2000;14(1):148–159.
  4. Fernandez-Gonzalo R, et al. Muscle damage responses and adaptations to eccentric-overload resistance exercise in men and women. Eur J Appl Physiol. 2017;117(4):825–833.

### 2.57. Artificial Intelligence and Bioinformatics-Driven Exploration of Bioactive Compounds for Enhanced Athletic Performance

Horacio Pérez-Sánchez ^1,^*

^1^  Structural Bioinformatics and High Performance Computing (BIO-HPC) Research Group, HiTech Innovation Hub, Universidad Católica de Murcia (UCAM), Spain

* Correspondence: hperez@ucam.edu

**Abstract:** The discovery of natural and legal substances for enhancing sports performance is essential for avoiding the adverse effects of synthetic alternatives. However, effective natural substances are limited, highlighting an unexplored area ripe for discovery. Here, we introduce a high-performance computing platform leveraging AI, bioinformatics, and chemoinformatics to identify bioactive compounds targeting key proteins related to exercise capacity, including AMPK^1^, PGC-1α^2^, SIRT1^3^, myostatin^4^, FGF21^5^, β2-adrenergic receptors^6^, and ERRα^7,^ among many others. We summarize the potential identification of compounds that enhance sports performance through mitochondrial function, muscle adaptation, improved muscle contractility, and better oxygen delivery. Our platform, validated in drug discovery (more than 100 publications and 10 patents), seamlessly translates to applications in sports performance enhancement, representing a versatile tool for discovering novel, safe, performance-enhancing substances.

**Keywords:** bioinformatics; exercise capacity; artificial intelligence; protein targets; sports performance enhancement

**Funding:** This research received no external funding.

**References**
  1. Spaulding, H. R. & Yan, Z. AMPK and the Adaptation to Exercise. *Annu Rev Physiol* **84**, 209–227 (2022).
  2. Liang, H. & Ward, W. F. PGC-1alpha: a key regulator of energy metabolism. *Adv Physiol Educ* **30**, 145–151 (2006).
  3. Vargas-Ortiz, K., Pérez-Vázquez, V. & Macías-Cervantes, M. H. Exercise and Sirtuins: A Way to Mitochondrial Health in Skeletal Muscle. *Int J Mol Sci* **20**, 2717 (2019).
  4. Fedoruk, M. N. & Rupert, J. L. Myostatin inhibition: a potential performance enhancement strategy? *Scand J Med Sci Sports* **18**, 123–131 (2008).
  5. Luo, X. et al. Endurance Exercise-Induced Fgf21 Promotes Skeletal Muscle Fiber Conversion through TGF-β1 and p38 MAPK Signaling Pathway. *International Journal of Molecular Sciences* **24**, 11401 (2023).
  6. Collomp, K., Le Panse, B., Candau, R., Lecoq, A.-M. & De Ceaurriz, J. Beta-2 agonists and exercise performance in humans. *Science & Sports* **25**, 281–290 (2010).
  7. Billon, C. et al. Synthetic ERRα/β/γ Agonist Induces an ERRα-Dependent Acute Aerobic Exercise Response and Enhances Exercise Capacity. *ACS Chem. Biol.* **18**, 756–771 (2023).

### 2.58. The Dose–Response Relationship of the Copenhagen Adduction Exercise for Prevention of Groin Problems in Hockey Players

Marcos Quintana-Cepedal ^1,2^, Eduardo López-Pérez ^1^, Miguel del Valle ^2,3^, Irene Crespo ^1^ and Hugo Olmedillas ^1,2,^*

^1^  Department of Functional Biology, University of Oviedo, Oviedo, Spain; marcosquintana99@gmail.com (M.Q.-C.); eduardolop1601@gmail.com (E.L.-P.); crespoirene@uniovi.es (I.C.)
^2^  Asturian Research Group in Performance, Readaptation, Training, and Health (AstuRES), Oviedo, Spain; miva@uniovi.es (M.V.)
^3^  Department of Cellular Morphology and Biology, University of Oviedo, Oviedo, Spain

* Correspondence: olmedillashugo@uniovi.es

**Abstract:** Groin problems are the most prevalent injuries among rink hockey players, yet no specific prevention program has been tested in this sport. The aim of this study is to assess the effectiveness of a single exercise intervention utilizing the Copenhagen Adduction Exercise (CAE) for preventing groin problems. Eighteen rink hockey clubs comprising 162 players implemented a 28-week intervention involving the CAE. Teams could conduct none, one, or two weekly sessions, categorized into high (≥34 sessions; 1.5 per week) or low (<34 sessions) compliance groups based on weekly session frequency. Groin problem prevalence was assessed monthly using the Oslo Sports Trauma Research Center Overuse questionnaire. Generalized estimating equations were used to compare the prevalence between groups over time. Monthly questionnaire responsiveness ranged from 86 to 93%, with 16 to 27% of participants reporting groin problems monthly. The odds of reporting groin issues decreased over the season (OR = 0.83; 95% CI: 0.66–1.06), with a non-significant reduction in injuries in the low compliance group (OR = 0.86; 95% CI: 0.58–1.27) compared to the high compliance group. Incorporating the CAE reduced the odds of sustaining groin problems in rink hockey players. However, no significant difference was observed between high and low compliance groups. A weekly volume of CAE is recommended to prevent these injuries in rink hockey players.

**Keywords:** adductor muscles; injury prevention; female athletes

**Funding:** This research received no external funding.

**References**
  1. Quintana-Cepedal M, De La Calle O, Olmedillas H. Can the Copenhagen Adduction Exercise Prevent Groin Injuries in Soccer Players? A Critically Appraised Topic. *J Sport Rehabil*. 2024;33(1):45–48.
  2. Whittaker JL, Small C, Maffey L, Emery CA. Risk factors for groin injury in sport: an updated systematic review. *Br J Sports Med*. 2015;49(12):803–809.

### 2.59. Relationship Between Maximal Handgrip Strength and Peak Power in University Athletes

Paola Rubiano ^1^, David Parra ^2^ and Jairo Fernandez ^3,^*

^1^  Research teacher at the Faculty of Education, Bachelor of Physical Education, Minuto de Dios University Corporation UNIMINUTO, Bogotá, Colombia; prubiano@uniminuto.edu
^2^  Research teacher at the Faculty of Education, Bachelor of Physical Education, Minuto de Dios University Corporation UNIMINUTO, Bogotá, Colombia; davidparraster@gmail.com
^3^  Research Professor, Faculty of Education, Bachelor’s degree in Sports UPN Universidad Pedagogic Nacional de Bogotá, Colombia; jairofdz@gmail.com

* Correspondence: andreitavoleibol@hotmail.com

**Abstract:** The purpose of this study was to investigate the levels of physical activity and the relationship between maximal handgrip strength and peak power of the lower limbs in university athletes from different ethnic groups [1,2]. This descriptive, observational, and cross-sectional research was conducted between June 2018 and June 2019, utilizing a variety of specific tests to evaluate the variables involved. Tests included maximal strength in squat, handgrip strength, peak power in squat, countermovement jump, and the Wingate test. Participants were selected from universities in Bogotá and comprised a diverse sample in terms of gender, ethnicity, and level of physical activity. The results indicated significant differences in handgrip strength between the right and left hands, regardless of gender, ethnicity, and level of physical activity. A strong correlation was observed between the handgrip strength of the upper limbs and the power of the lower limbs during the countermovement, as well as with peak power in squat [3]. The correlations showed that handgrip strength in the right hand (r = 0.242; *p* < 0.001) and left hand (r = 0.252; *p* < 0.001) had low correlations with maximum and minimum RPM, although they were significant. Subjects with higher dynamometry values in both hands also showed higher maximum RPM and lower minimum RPM (right r = 0.198; *p* < 0.001; left r = 0.165; *p* = 0.003). The countermovement showed moderate correlations with maximum RPM (r = 0.415; *p* < 0.001) and minimum RPM (r = 0.353; *p* = 0.001). The results of the factorial ANOVA model showed significant findings for the variable peak power, measured in kilograms. A significant interaction between gender and ethnic typology was evidenced (*p* = 0.040). However, this effect was not replicated when measured in terms of watts (*p* = 0.098). Additionally, no significant interactions were found between gender and physical activity, ethnic typology and physical activity, nor between gender, ethnic typology, and physical activity. Analyzing the total sample showed that handgrip strength presented moderate correlations with the countermovement jump, peak power in Wingate, and peak power in squat. On the other hand, the countermovement jump showed a high correlation with peak power in squat and a moderate correlation with peak power in Wingate. Finally, a moderate correlation was found between peak power in Wingate and peak power in squat. This study underscores the importance of handgrip strength as a relevant indicator of lower limb power in young athletes, regardless of their ethnicity and level of physical activity [1,4]. These findings can contribute to the design of more effective and personalized training programs, optimizing athletic performance in various disciplines.

**Keywords:** muscular strength; muscular power; young adults; sports training

**Funding:** We thank the university students from Bogotá who voluntarily participated in this study and the universities involved for their support and collaboration in carrying out the tests.

**References**
  1. González-Badillo J, Rodríguez-Rosell D, Sánchez-Medina L, Gorostiaga M, Pareja-Blanco F. Maximal intended velocity training induces greater gains in bench press performance than deliberately slower half-velocity training. European Journal of Sport Science. 2014;14(8):772–781. https://onlinelibrary.wiley.com/doi/10.1080/17461391.2014.905987
  2. Knuttgen HG, Kraemer WJ. Terminology and measurement in exercise performance. J Appl Sport Sci Res. 1987;1(1):1–10. http://dx.doi.org/10.1519/00124278-198702000-00001
  3. Platonov VN. La preparación física en el deporte de alto rendimiento. Barcelona: Paidotribo; 2001.
  4. Wilmore JH, Costill DL. Physiology of sport and exercise. Champaign: Human Kinetics; 2007.

### 2.60. Are Exogenous Ketones Truly Useful as an Ergogenic Aid in Cycling? A Systematic Review

Juan Carlos Salinas Camargo ^1^, Francisco Javier Martínez Noguera ^2^ and Cristian Marín Pagán ^2,^*

^1^  Research Center for High-Performance Sport, Campus de los Jerónimos, Catholic University of Murcia, Guadalupe, 30107 Murcia, Spain; jcsalinas2@ucam.edu, fjmartinez3@ucam.edu, cmarin@ucam.edu

* Correspondence: jcsalinas2@ucam.edu; Tel.: 968 278 566

**Abstract:** Ketone bodies (KBs) are small molecules derived from lipids, synthesized in the liver from fatty acids to replace blood glucose as a substrate for peripheral tissues during periods when carbohydrate (CH) availability is limited (1). The ketogenic diet increases KBs and circulating fat concentrations while reducing glucose utilization during endurance exercise. However, there does not appear to be an increase in physical performance (2), possibly due to the severe CH restriction, which significantly reduces muscle glycogen stores at the beginning of the activity and worsens glycolytic flow during high-intensity exercise (3). To avoid the limitations of CH restriction, the consumption of exogenous ketones has been proposed as an alternative strategy to increase circulating KB concentrations. Acute supplementation with exogenous ketones could be a strategy to consider for preserving muscle glycogen during exercise, as it could have significant implications in events where glycogen reserves are a limiting factor (4). A literature search was conducted in the electronic databases PubMed and Cochrane Library; studies were included without date or language limitations. The search equation was (ketone bodies OR ketone ester OR β-hydroxybutyrate OR 1,3-butanediol) AND (cycling OR cycling performance OR time-trial OR VO2max OR endurance exercise OR training). The included studies met the inclusion criteria (randomized controlled trials, cyclists, administration of ketone esters or ketone salts, and performed a time trial). A total of 280 studies were identified, of which eight were included in the review. Acute intake of ketone esters (0.35 g/kg of body weight) appears to be sufficient to achieve a β-hydroxybutyrate (β-HB) concentration in the range of 1–4 mM, in which ergogenic effects are manifested. Ketone esters seem to have an advantage over acetoacetate diester and ketone salts, as they cause fewer gastrointestinal discomforts. There is a need to divide the doses to be ingested, as this results in better gastrointestinal sensation and maintains high β-HB concentrations for a longer period.

**Keywords:** ketone bodies; ketone ester; β-hydroxybutyrate; 1,3-butanediol; cycling; cycling performance; time trial; VO_2_max; endurance exercise; training

**Funding:** This research did not receive external funding.

**References**
  1. Robinson EJ, Taddeo MC, Chu X, Shi W, Wood C, Still C, et al. Aqueous Metabolite Trends for the Progression of Nonalcoholic Fatty Liver Disease in Female Bariatric Surgery Patients by Targeted H-1-NMR Metabolomics. Metabolites. 2021;11(11).
  2. Leckey JJ, Ross ML, Quod M, Hawley JA, Burke LM. Ketone Diester Ingestion Impairs Time-Trial Performance in Professional Cyclists. Front Physiol. 2017;8:806.
  3. McSwiney FT, Doyle L, Plews DJ, Zinn C. Impact Of Ketogenic Diet On Athletes: Current Insights. Open Access J Sports Med. 2019;10:171–83.
  4. Cox PJ, Kirk T, Ashmore T, Willerton K, Evans R, Smith A, et al. Nutritional Ketosis Alters Fuel Preference and Thereby Endurance Performance in Athletes. Cell metabolism. 2016;24(2):256–68.

### 2.61. Is the Sit-to-Stand Test an Estimator of Lower Limb Strength in Older Adults?

**Nidia Sánchez-García ^1^, Francisca Reyes-Merino ^1^, Diego González-Martín ^1^, Javier Santos-Pérez ^1^, Ángel Gallego-Sellés ^2^ and José Antonio de Paz Fernández ^1^**

^1^  Universidad de León; Institute of Biomedicine; Campus Vegazana s/n 24071, León, Spain
^2^  Department of Physical Education and Research Institute of Biomedical and Health Sciences (IUIBS) Universidad de Las Palmas de Gran Canaria, Las Palmas de Gran Canaria, Spain

* Correspondence: nisag@unileon.es

**Abstract:** Muscle strength is one of the fundamental determinants of the ability and functional independence of older adults (1). Low levels of muscle strength are associated with functional disability, risk of falls and fractures, and higher mortality from all causes (2,3). One of the best strategies to prevent or delay loss of function is strength training (4). According to the WHO, older adults should work on their muscle strength two or more times a week. To estimate physical fitness and schedule strength training, it is necessary to evaluate this ability. In older adults, it is often performed with indirect tests such as Sit to Stand (StS) of five repetitions (5 rept) or 30 s (30 sg), Timed Up and Go (TuG), or elbow bending with a dumbbell. However, there are some doubts about whether these tests actually determine strength or other aspects such as agility, mobility, etc. The gold standard for their evaluation is the measurement of 1RM, or the maximum isometric contraction. The aim of this study was to compare the relationship between 1RM and isometric maximum (MVIC) with indirect tests involving knee extenders. To this end, 60 subjects (30 men and 30 women) with an average age of 69.92 ± 0.84 years, not institutionalized, and resident in the city of León were evaluated. StS 5 rept and 30 sg, 1RM, and isometric (ChronoJump gauge) tests were performed on the leg extension machine (BH Nevada) and 1RM on a 45° inclined leg press (Gerva Sport). We found only a moderate correlation between the maximum force values of 1RM in press and StS 30 sg in the sample (r = 0.336). For women, there was also a moderate correlation between the leg press test and the StS 5 rept (r = 0.519). In conclusion, we cannot say that the indirect StS test is a good method for measuring the strength of lower limbs in older adults, as it may be influenced by factors such as agility or joint mobility.

**Keywords:** muscle strength; aging; indirect test; functional independence

**Funding:** This research was funded by Ministerio de Ciencia e Innovación, grant number PID2021-125354OB-C21. AGS is a beneficiary of the Catalina Ruiz training grant program for researchers from the Consejería de Economía, Conocimiento y Empleo, as well as the Fondo Social Europeo.

**References**
  1. Mayer, F., Scharhag-Rosenberger, F., Carlsohn, A., Cassel, M., Müller, S., & Scharhag, J. The intensity and effects of strength training in the elderly. *Deutsches Ärzteblatt International* **2011**,*108*, 359–364.https://doi.org/10.3238%2Farztebl.2011.0359
  2. Li, R., Xia, J., Zhang, X. I., Gathirua-Mwangi, W. G., Guo, J., Li, Y., Mckenzie, S. & Song, Y. Associations of muscle mass and strength with all-cause mortality among US older adults. *Medicine and science in sports and exercise* **2018**, 50, 458–467. https://doi.org/10.1249%2FMSS.0000000000001448
  3. Studenski, S., Peters, K., Alley, D., Cawthon, P., McLean, R., Harris, T., Ferucci, L., Guralnik, J., Fragala, M., Kenny, A., Kiel, D., Kritchevsky, S., Shardell, M., Dam, T. T. & Vassileva, M. The FNIH sarcopenia project: rationale, study description, conference recommendations, and final estimates. *Journals of Gerontology Series A: Biomedical Sciences and Medical Sciences* **2014,** *69*, 547–558. https://doi.org/10.1093/gerona/glu010
  4. Mitchell, W. K., Williams, J., Atherton, P., Larvin, M., Lund, J., & Narici, M. Sarcopenia, dynapenia, and the impact of advancing age on human skeletal muscle size and strength; a quantitative review. *Frontiers in physiology* **2012**,3, 1–18. https://doi.org/10.3389/fphys.2012.00260.

### 2.62. Effects of Different Velocity Loss Thresholds with and Without Blood Flow Restriction During the Squat Exercise on Strength Gains and Jump Performance

Juan Sánchez-Valdepeñas ^1,2,^*, Iván Asín-Izquierdo ^1,3^, Pedro J. Cornejo-Daza ^1,2,4^, Gonzalo Mariscal-Campón ^2^, Ruggero Romagnoli ^5^, Eduardo Saez de Villarreal ^2^ and Fernando Pareja-Blanco ^1,2^

^1^  Science Based Training Research Group, Department of Sports and Computer Sciences, Universidad Pablo de Olavide, Seville, Spain
^2^  Faculty of Sport Sciences, Department of Sports and Computer Sciences, Universidad Pablo de Olavide
^3^  Department of Musical, Plastic and Corporal Expression, Faculty of Social and Human Sciences, University of Zaragoza, Teruel, Spain
^4^  Department of Human Movement and Sport Performance, University of Seville, 41013 Seville, Spain
^5^  eCampus University, Rome, Italy

* Correspondence: jsanmat@upo.es

**Abstract:** A low-to-moderate velocity loss (VL) threshold is enough to enhance neuromuscular performance and strength-derived adaptations [1]. Regarding blood flow restriction (BFR) during resistance training (RT), when comparing different degrees of effort on long-term adaptations, it has been demonstrated that achieving muscle failure is not necessary for maximizing strength gains [2,3]. However, it is worth noting that no previous research has analyzed the long-term effects against the same VL thresholds in the BFR-RT contexts in comparison to free flow (FF) conditions. Therefore, this study aimed to analyze the effects on strength-derived adaptations and jump performance following four RT programs with two blood flow conditions (FF vs. BFR) and two different VL thresholds (20% and 40% VL) using the full squat (SQ) exercise. Fifty-two strength-trained men participated in a randomized controlled trial. They followed an 8-week (16 sessions) RT program from 55% to 70% of their 1-repetition maximum (1RM) (FF20: n = 14; BFR20: n = 13; FF40: n = 12; BFR40: n = 13). All groups performed the same number of sets (three) and had the same inter-set recovery periods (2 min) per session. For the BFR groups, 50% of arterial occlusion pressure was applied and maintained during inter-set recovery. The following tests were carried out: 1) countermovement jump (CMJ); 2) progressive loading test in SQ; and 3) a fatigue test with a load corresponding to 70% 1RM at pre-training measurements until the velocity was less than 0.5 m·s^−1^. The following variables were analyzed: CMJ height, 1RM load, average velocity attained against low, high, and all absolute loads common at pre- and post-training (AV ≥ 1, AV < 1, and AV), and maximal number of repetitions (MNRs) performed during the fatigue test. Regarding jump performance, a significant “VL × time” interaction (*p* = 0.02) in favor of the 20% VL groups and a significant “time” effect (*p* < 0.001) were reported. For 1RM and strength-derived outcomes from the “progressive loading” test, significant “VL × time” interactions were reported (from *p* = 0.01 to *p* = 0.05), also in favor of 20% VL groups. There was a significant “time effect” for all variables, with all groups showing significant intra-group improvements. Additionally, all groups showed significant enhancements in MNRs. Prescribing a certain degree of effort via VL induces similar gains in jump performance and leg strength adaptations regardless of the blood flow condition, with the gains being maximized at a moderate VL threshold (i.e., 20%). Therefore, a high level of fatigue is not required under FF and BFR conditions.

**Keywords:** velocity-based training; full squat; strength gains; jump performance; blood flow restriction

**Funding:** This study was funded by the Ministerio Espanol de Innovación y Ciencia (AEI/10.13039/501100011033; grant reference PID2020-117915RA-I00) and by the Ministerio de Universidades [MCIN/AEI/10.12039/501100011033] through grant [FPU19/00891] for University Professor Training. The funders had no role in study design, data collection, analysis, decision to publish, or preparation of the manuscript.

**References**
  1. Pareja-Blanco, F.; Alcazar, J.; Sánchez-Valdepeñas, J.; Cornejo-Daza, P.J.; Piqueras-Sanchiz, F.; Mora-Vela, R.; Sanchez-Moreno, M.; Bachero-Mena, B.; Ortega-Becerra, M.; Alegre, L.M. Velocity Loss as a Critical Variable Determining the Adaptations to Strength Training. *Med Sci Sports Exerc* **2020**, *52*, 1752–1762, doi:10.1249/MSS.0000000000002295.
  2. Bjornsen, T.; Wernbom, M.; Paulsen, G.; Berntsen, S.; Brankovic, R.; Stalesen, H.; Sundnes, J.; Raastad, T. Frequent blood flow restricted training not to failure and to failure induces similar gains in myonuclei and muscle mass. *Scand J Med Sci Sports* **2021**, *31*, 1420–1439, doi:10.1111/sms.13952.
  3. Sieljacks, P.; Degn, R.; Hollaender, K.; Wernbom, M.; Vissing, K. Non-failure blood flow restricted exercise induces similar muscle adaptations and less discomfort than failure protocols. *Scand J Med Sci Sports* **2019**, *29*, 336–347, doi:10.1111/sms.13346.

### 2.63. Artificial vs. Natural Grass: Differences in Technical and Tactical Demands of Professional Football

**Thor Sigurdsson ^1^, Konstantinos Spyrou ^1,2^ and Tomás T. Freitas ^1,2,3^**

^1^  UCAM Research Center for High Performance Sport, UCAM Universidad Católica de Murcia, Murcia, Spain
^2^  Facultad de Deporte, UCAM Universidad Católica de Murcia, Murcia, Spain
^3^  NAR Nucleus of High Performance in Sport, São Paulo, Brazil

* tsigurdsson@alu.ucma.edu, kspyrou@ucam.edu, tfreitas@ucam.edu

**Abstract:** Football has become faster and more technical, with teams running further at higher speeds and passing more often and with greater accuracy (particularly short and medium passes) (1). Previous studies comparing passing patterns on artificial grass (AG) vs. natural grass (NG) have shown that the number of total passes is significantly higher on the former, mainly due to short passes and passing in the midfield (2). Research has also shown that total accurate passing is higher on AG than on NG; conversely, direct shots are more frequent on NG than AG (3). Thus, this study aimed to compare the technical and tactical demands of professional football matches played on AG vs. NG over one season. A set of 573 Wyscout^®^ reports (AG: 308; NG: 265) from home and away matches of 24 teams was analyzed, and a total of 42 technical and tactical variables were collected. A linear mixed model analysis was conducted in Jamovi statistical package (2020; Version 1.2). Expected goals, match tempo, and average passes per possession were significantly higher on AG than on NG. Interceptions, clearances, long passes, and long pass percentage were significantly higher on NG versus AG. A significantly greater number of total duels and total duels won occurred on NG than on AG, mainly due to aerial duels and aerial duels won, which were significantly higher on the former. No difference was observed in offensive duels or defensive duels when comparing both surfaces. There was a significant difference in accurate crosses and positional attack with a shot, where both were higher on AG; however, successful sliding tackles were significantly higher on NG. Total passes, backward passes, and lateral passes were all significantly higher on AG than on NG. Moreover, accurate passes total-, backward-, lateral-, forward-, to final third-, and progressive were all significantly greater on AG than on NG. No difference was found for any other variables. The results of this study indicate that the playing surface may affect both technical and tactical demands, with players passing the ball more often and with superior accuracy, and being more likely to score a goal on AG than on NG. In contrast, when playing on the latter, players seemed to execute more duels and longer passes compared to the former.

**Keywords:** soccer; technical demands; tactical demands; artificial grass; natural grass; passing; defensive; attacking

**Funding:** This research received no external funding.

**References**
  1. Andersson, H., Ekblom, B., & Krustrup, P. (2008). Elite football on artificial turf versus natural grass: Movement patterns, technical standards, and player impressions. Journal of Sports Sciences, 26(2), 113–122. https://doi.org/10.1080/02640410701422076
  2. Ávalos Guillén, J. C., Gutiérrez Vargas, R., Araya Vargas, G. A., Sánchez Ureña, B., Gutiérrez Vargas, J. C., & Rojas Valverde, D. (2017). Effects of artificial turf and natural grass on physical and technical performance of professional soccer players. MHSalud: Movimiento Humano y Salud, 14(1 (Septiembre-Enero)), 1.
  3. Barnes, C., Archer, D. T., Hogg, B., Bush, M., & Bradley, P. S. (2014). The Evolution of Physical and Technical Performance Parameters in the English Premier League. International Journal of Sports Medicine, 1095–1100. https://doi.org/10.1055/s-0034-1375695

### 2.64. Effects of Core Centering Training on Balance, Trunk Control, and Athletic Performance in Adolescent Female Volleyball Players

Fioretta Silvestri ^1,#^, Arianna Fogliata ^2,#,^*, Lorenzo Marcelli ^1^ and Davide Curzi ^1^

^1^  Department of Humanities, Movement and Education Sciences, University “Niccolò Cusano”, Rome, Italy; fioretta.silvestri@unicusano.it; lorenzo.marcelli@unicusano.it; davide.curzi@unicusano.it
^2^  University of Vanvitelli & Pegaso telematic University. fogliataarianna@gmail.com

^#^  These authors contributed equally to this work

* Correspondence: fogliataarianna@gmail.com

**Abstract:** The ability to recover a stable body position in space is a fundamental skill in sports involving jumping actions, such as volleyball. During growth, the ability to manage body weight can be altered, and specific core stability training is recognized as essential for both motor control and sports performance. However, due to the limited duration of the training session, it is crucial to find strategies to integrate core exercises into the training program sustainably in the long term for young athletes. Centration technique, which is a conscious modulation of intra-abdominal pressure (IAP) during a motor gesture, could enhance muscle activation and core stability in complex movements. This study aimed to analyze the effects of incorporating the centering technique into a simple core training protocol on balance, trunk control, and athletic performance in a sample of adolescent competitive volleyball players. Forty-four female volleyball athletes, randomly divided into three different groups (G1 = 14 subjects; G2 = 16 subjects; G3 = 14 subjects), participated in the study. For 8 weeks, athletes undertook 30 min of differentiated intervention training (G1 = centering-core training; G2 = core training; G3 = standard aerobic conditioning) two times per week in addition to the volleyball training. Balance ability (Berg Balance Scale (BBS) and Stork balance stand test (SBST)), trunk control (TCT), lower limb explosive strength (Broad Jump Test (BJT), Squat Jump (SJ), and Drop Jump (DJ) test), and aerobic capacity (Cooper Test (CT)) were evaluated at the beginning (T1), end (T2), and 12 weeks after the end of the intervention period (T3). Statistical analysis showed significant increases in G1 and G2 in BBS, SBST, TCT, CT, and DJ at T2 compared to T1, which lasted until T3 in both groups. In addition, G1 showed increases in the SJ test at T2 and a higher improvement rate in SBST and DJ compared to G2. G3 increased only in CT at T2 compared to T1. These results suggest that centration and core training can enhance balance, trunk control, and athletic performance in young volleyball athletes. Moreover, integrating the centering technique into core exercises leads to a greater improvement in balance and lower limb explosive strength compared to simple core stability training. These data could be useful for optimizing strength and conditioning programs for young athletes, who often have limited time and availability.

**Keywords:** centration; core training; volleyball; stability, balance

**Funding:** This research received no external funding.

**References**
  1. De Bernardi, F.; Fogliata, A., & Garassino, A. The multifunctionality of the diaphragm: Beyond respiratory mechanics. *MOJ sports med*, **2024**, *7(I)*, 9–13.
  2. Mueller, S.; Mueller, J.; Stoll, J.; & Mayer, F. Effect of Six-Week Resistance and Sensorimotor Training on Trunk Strength and Stability in Elite Adolescent Athletes: A Randomized Controlled Pilot Trial. *Frontiers in Physiology*, **2022**, *13*: 802315.
  3. Read, P.; Oliver, J. & Lloyd, R. Seven Pillars of Prevention: Effective Strategies for Strength and Conditioning Coaches to Reduce Injury Risk and Improve Performance in Young Athletes. *Strength & Conditioning Journal*, **2020**, *42*(6), 123–130.

### 2.65. Analyzing the External Match Load in Football: A Case Study of Three Coaches in One Season

Konstantinos Spyrou ^1,2,3,^*, José María Escudero Ferrer ^2^, Pedro E. Alcaraz ^1,2,3^, Tomás T. Freitas ^1,2,3,^, Javier Raya-González ^4^ and Luis Manuel Martínez-Aranda ^5,6^

^1^  UCAM Research Center for High Performance Sport, UCAM Universidad Católica de Murcia, Murcia Spain; kspyrou@ucam.edu, palcaraz@ucam.edu, tfreitas@ucam.edu
^2^  Facultad de Deporte UCAM Universidad Católica de Murcia, Murcia Spain; jmescudero@ucam.edu
^3^  Strength and Conditioning Society, Murcia, Spain;
^4^  Department of Specifics Didactics, Faculty of Education Sciences, University of Córdoba, Spain; rayagonzalezjavier@gmail.com
^5^  Physical and Sports Performance Research Centre, Faculty of Sports Sciences, Pablo de Olavide University, Seville, Spain; lmmarara@upo.es
^6^  SEJ-680: Science-Based Training (SBT) research group, Pablo de Olavide University, Seville, Spain

* Correspondence: kspyrou@ucam.edu

**Abstract:** Football is a high-intensity intermittent team sport and is considered a complex, self-organized, unstable, unpredictable, and highly dynamic system in which coaches and players try to maintain the stability of their own attacking and defending systems while disrupting the opponents’ structure (1, 2). Coaches play a crucial role in the team’s success (3). The aim of this study was to analyze whether the external match load changes when comparing three different coaches with the same players in the same season. Twenty professional players in a football team participated in this study. The external load was quantified using GPS technology. The season consisted of 33 games in total, of which 13 matches were played under the instructions of coach (C) 1, 7 under C2, and 12 under C3. A linear mixed model was used for statistical analysis. Players performed a higher number of accelerations (*p* = 0.004), accelerations (*p* = 0.001), and decelerations (*p* = 0.019) per minute under C2 compared to C3. No further significant differences were found in total distance covered, distance per minute, distance at different velocities, number of sprints and decelerations, and power plays between the three coaches. In conclusion, this study demonstrates that different coaches may influence specific external load metrics, such as accelerations and decelerations, highlighting the critical role coaches play in shaping player dynamics and overall team strategy.

**Keywords:** team sports; coaching; performance

**Funding:** This research received no external funding.

**References**
  1. Davids K, Araújo D, Correia V, Vilar L. How small-sided and conditioned games enhance acquisition of movement and decision-making skills. Exerc Sport Sci Rev. (2013) 41(3):154–61.
  2. Garganta J. Trends of tactical performance analysis in team sports: bridging the gap between research, training and competition. Rev Port Ciênc Desporto. (2009) 9(1):81–9.
  3. García-Aliaga A, Rivas-González P, Martín-Castellanos A, Cordón-Carmona A, Muriarte Solana D, Mon-López D, et al. ¿Cómo afecta el cambio de entrenador al rendimiento físico de los jugadores de fútbol? Apunts Educ Física Deport. 1 de enero de 2024;(155):50–8.

### 2.66. Impact of Breathing Patterns on Short-Term Recovery (25-Second Rule) in Youth Tennis Players: A Comparative Study

Srinath N.U. ^1^ and Udayanga M.A.S. ^1,^*

^1^  University of Sri Jayewardenepura, Sri Lanka

* Correspondence: sajithudayanga@sjp.ac.lk; Tel.: +94763663246

**Abstract:** Tennis has a tight regulation that states that players can wait a maximum of 25 s between points in a single game [1,2]. This study examines how different breathing patterns affect youth-level tennis players’ short-term recovery during the 25 s rule between points. In this crossover experimental study, fifteen players from three state universities in Sri Lanka (n = 15) made up the study’s sample size (Age = 22.67 ± 1.05 (SD) years, Body Weight = 57.64 ± 8.37 kg, Height = 168.60 ± 12.62 cm, Body Mass Index = 20.33 ± 2.45 kg/m^2^, Competitive Experience = 7.73 ± 2.12 years). The research employed the modified ‘Two-Line Drill Wide Mode’, a standardized tennis drill, to simulate competitive play [1]. The study assessed recovery using the vertical jump, 10 m sprint, and readiness level tests before and after the drill. Three breathing patterns (6 breaths, 9 breaths, and 12 breaths per minute) were randomly assigned to participants during the 25 s period. The Shapiro–Wilk normality and homogeneity of variance tests were used to analyze the pre-test and post-test data. A one-way ANOVA was used for normally distributed data, while the Friedman Test was employed for non-normally distributed data. The pre-test values for vertical jump and for sprint tests were not significant between conditions (*p* = 0.943 and *p* = 0.549, respectively). There were no statistically significant differences between the breathing patterns in the outcomes of the vertical jump test (*p* = 0.997). Similarly, there were no significant differences after the Friedman Test in the 10 m sprint test (*p* = 0.284). However, there was a significant difference (*p* = 0.000) in post-test results among the breathing patterns according to the readiness level test. The 6- (Pre = 8.30 ± 1.00, Post = 7.5 ± 1.10) and 9-breath patterns (Pre = 8.1 ± 0.80, Post = 7.30 ± 1.10) indicated a slight decrease in readiness, but the 12-breath pattern (Pre = 8.40 ± 1.20, Post = 5.50 ± 1.10) showed a decline in readiness. In conclusion, the various breathing patterns (6, 9, and 12 breaths) used in this study did not significantly affect the performance variables (vertical jump, 10 m sprint). However, they significantly impacted psychological variables, such as readiness level.

**Keywords:** recovery; breathing patterns; tennis; 25 s rule

**Funding:** This research received no external funding.

**References**
  1. Morais, J. E., Bragada, J. A., Silva, R., Nevill, A. M., Nakamura, F. Y., & Marinho, D. A. Analysis of the physiological response in junior tennis players during short-term recovery: Understanding the magnitude of recovery until and after the 25-s rule. *Int J Sports Sci Coach* **2023**, 18(4), 1208–1216.
  2. Pluim BM, Jansen MG, Williamson S, Berry C, Camporesi S, Fagher K, Heron N, van Rensburg DC, Moreno-Perez V, Murray A, O’Connor SR. Physical demands of tennis across the different court surfaces, performance levels and sexes: a systematic review with meta-analysis. *Sports medicine* **2023**, 53(4), 807–36.

### 2.67. Impact of Physical Activity and Selected Relaxation Techniques on the Quality of Life of Patients with Irritable Bowel Syndrome (IBS)

Ewa Szura ^1,^*

^1^  University of Applied Sciences in Nysa; ewa.szura@pans.nysa.pl

* Correspondence: ewa.szura@pans.nysa.pl; Tel.: +48609611153

**Abstract:** Gastrointestinal disorder complaints significantly impair the quality of life, often arousing concern for the patient and their family. The lack of effective pharmacological options to alleviate patients’ complaints, at a time of great advances in medicine and pharmacotherapy, prompted the search for alternative methods of treating patients. Irritable bowel syndrome, which affects about 20% of the world’s population, is characterized by abdominal pain, bloating, and bowel movement disorders. Patients often have mental disorders and even symptoms of depression. Difficulties treating or only alleviating symptoms prompted a search for unconventional methods involving the cooperation of doctors, psychologists, and coaches. The purpose of this study was to evaluate the effect of physical exercise on the quality of life of patients with IBS. A total of 41 women with diagnosed irritable bowel syndrome qualified for the study. To assess the effectiveness of the method used, the validated IBS-QOL (irritable bowel syndrome–quality of life) questionnaire was used. The study used an original training program based on the basic steps used in a fitness lesson, yoga positions for improving intestinal motility, and relaxation training: autogenic by Schultz and Jacobson. Twenty-nine women completed the two-month training cycle, in line with the assumptions of the study. The data were analyzed using the Statistica program. The following methods were used: Student’s *t*-test for dependent variables, Wilcoxon pairwise order test, McNemar’s chi2 test, and Spearman’s R correlation coefficient. The questionnaire contained questions grouped into nine dimensions: dysphoria, activity interference, body image, health preoccupation, food avoidance, social response, sexuality, relationships, and overall. Statistically significant improvements were shown in all nine QOL dimensions studied. The results confirmed the existence of a 12.5% relationship between physical activity and the quality of life of IBS patients. Of the QOL dimensions analyzed, the largest difference was shown in the areas of sexuality, health preoccupation, body image, and dysphoria, while the smallest was in the activity interference dimension.

**Keywords:** irritable bowel syndrome; flatulence; physical activity; quality of life

**Funding:** This research received no external funding.

**References**
  1. Pietrzak A., Skrzydło-Radomańska B., Mulak A., Lipinski M., Małecka-Panas E., Reguła J., Rydzewska G. Guidelines on the management of irritable bowel syndrome. *Gastroenterology Review* Poland, 2018; 13 (4): 167–196.
  2. Buono J, Rybon T, Flores N. Health-related quality of life, work productivity, and indirect costs among patients with irritable bowel syndrome with diarrhea. *Health and Quality of Life Outcomes*, 2017; 15 (1): 35.
  3. Patrick D L, Drossman D A, Frederick I O, DiCesare J, Puder K L.: Quality of life in persons with irritable bowel syndrome: development and validation of a new measure. *Dig Dis Sci* 1998; 43(2): 400–11.
  4. Poniewierka E., Szura E., Valach P. Effects of physical activity on the symptoms of irritablebowel syndrome (IBS). *Gastroenetrology Review*, EISSN: 1897-4317 ISSN: 1895-5770; 2024

### 2.68. Feasibility and Effectiveness of Exercise Snacking in Older Adults: A Randomized Controlled Pilot Trial

James F Timmons ^1^, Conor Murphy ^1,3^, Billy Twomey ^3^, Connor Montgomery ^3^ and Brendan Egan ^1,2,^*

^1^  School of Public Health, Physiotherapy and Sports Science, University College Dublin, Ireland. james.timmons1@ucd.ie
^2^  School of Health and Human Performance, Dublin City University, Ireland brendan.egan@dcu.ie
^3^  School of Medicine, Trinity College Dublin Ireland

* Correspondence: brendan.egan@dcu.ie

**Abstract:** Sedentary behavior is a significant contributor to morbidity, particularly in older adults with chronic diseases. Exercise snacking, which involves brief, periodic bouts of higher-intensity exercise, presents a potential intervention. However, its feasibility and effectiveness in this population remain underexplored. This study aimed to evaluate the feasibility and effectiveness of an exercise snacking intervention in older adults enrolled in a Chronic Disease Management (CDM) program. A 4-week randomized controlled pilot trial involving 63 adults aged over 60 years (mean age 73.5 years) who were enrolled in a CDM program was conducted in a primary care setting. Participants were randomly assigned to either an exercise snacking group or an active control group. The intervention consisted of performing five resistance exercises over five minutes, at least twice daily, for four weeks. Outcomes assessed included retention, adherence, physical function (handgrip strength and sit-to-stand repetitions), and self-reported physical activity. Of the 63 participants, 53 completed the study, resulting in a retention rate of 84%. The median adherence rate was 79%. The exercise snacking group showed a non-significant trend towards improved handgrip strength compared to the control group (*p* = 0.117, η^2^p = 0.048). No significant differences were found in sit-to-stand repetitions between the groups (*p* = 0.831, η^2^p = 0.001). However, participants in the exercise group who adhered to the regimen showed significant improvements in sit-to-stand repetitions compared to controls (*p* = 0.041, η^2^p = 0.177). A short-term exercise snacking intervention is feasible and may improve handgrip strength and functional performance in older adults with chronic diseases, particularly among those who adhere to the regimen. Further research with larger sample sizes and strategies to enhance adherence is necessary to confirm these findings and fully explore the intervention’s potential.

**Keywords:** sedentary behavior; exercise snacking; chronic disease management; older adults; physical function

**Funding:** This research received no external funding.

**References**
  1. Perkin, O.J.; McGuigan, P.M.; Stokes, K.A. Exercise Snacking to Improve Muscle Function in Healthy Older Adults: A Pilot Study. *J. Aging Res.* 2019, *7516939*, 1–9. doi:10.1155/2019/7516939.
  2. Fyfe, J.J.; Dalla Via, J.; Jansons, P.; Scott, D.; Daly, R.M. Feasibility and Acceptability of a Remotely Delivered, Home-Based, Pragmatic Resistance ‘Exercise Snacking’ Intervention in Community-Dwelling Older Adults: A Pilot Randomised Controlled Trial. *BMC Geriatrics* 2022, *22*, 521. doi:10.1186/s12877-022-03207-z.
  3. Liang, M.T.C.; Dale, R.B. A Scoping Review of Home-Based Exercise Interventions for Older Adults to Improve Physical Function and Health-Related Quality of Life. *Int. J. Environ. Res. Public Health* 2021, *18*, 10469. doi:10.3390/ijerph182010469.
  4. Western, M.J.; Armstrong, M.E.G.; Islam, I. Promoting Physical Activity in Older Adults through Home-Based Interventions: Systematic Review and Meta-Analysis. *Health Psychol. Rev.* 2021, *15*, 74–97. doi:10.1080/17437199.2020.1747848.

### 2.69. Residual Eccentric Strength Deficits in Amateur Rugby Players with Previous Hamstring Injuries

Luciano Tomaghelli ^1^, Nicol van Dyk ^2,4^ and Eduardo Tondelli ^3,^*

^1^  Physical Activity and Sports Department, Universidad del Salvador, Argentina; Luciano.tomaghelli@usal.edu.ar
^2^  School of Public Health, Physiotherapy and Sport Science, University College Dublin, Dublin, Ireland; nicol.vandyk@ucd.ie
^3^  Research Center in Sports, Physical Activity and Medical Sciences, University of Buenos Aires, Buenos Aires, Argentina; etondelli@fmed.uba.ar
^4^  Section Sports Medicine, Faculty of Health Sciences, University of Pretoria, Pretoria, South Africa

**Abstract:** Hamstring injuries are the most common non-contact injury in amateur rugby union in Argentina [1]. Low levels of hamstring eccentric strength have been identified as a risk factor for hamstring injuries, with long-term residual deficits present in athletes with a history of hamstring injuries [2,3]. Increased hamstring eccentric strength also reduces the risk in the presence of known non-modifiable risk factors such as previous hamstring injury and age [4]. Thus, this study investigated i) whether Argentinean amateur rugby players with a history of hamstring injuries presented lower levels of eccentric hamstring strength and ii) compared hamstring eccentric strength levels between involved and uninvolved lower limbs in players who sustained hamstring injuries. Seventy-six rugby players (BM: 93 ± 14.1 kg; Backs: 37; Fwds: 39) competing in the URBA Primera A participated in the study during the 2023 pre-season. All participants were free of injury at the time. Twenty-five players had hamstring injuries (HI group) at least three months before the study. An involved limb was defined as one that sustained one or more hamstring injuries. The recently injured limb was considered if the subject presented bilateral injuries. Subjects performed three repetitions of the Nordic Hamstring curl exercise while force data were collected from load cells attached to the participants’ ankles (NordBord, VALD, Australia). Average bilateral maximal force and impulse, absolute and relative to body mass, were included in the HI and non-injured groups analysis and between involved and uninvolved limbs in the HI group. Statistical analysis included Welch’s T-Test for the between-group comparisons and Paired T-Test for the between-limb analysis in the HI group. Non-parametric tests were used if assumptions were not satisfied. Results indicated that players in the HI group produced significantly lower absolute force (*p* = 0.003, mean difference: −48.6 N, ES: −0.661) and relative maximal force (*p* = 0.007, mean difference: −0.53 N/kg, ES: −0.614) and absolute (*p* = 0.001, mean difference: −1311 N.s, ES: −0.750) and relative impulse (*p* = 0.003, mean difference: −13.9 N.s/kg, ES: −0.651). The involved limb in the HI group also showed lower levels of absolute (*p* < 0.001, mean difference: −39.9 N, ES: −0.794) and relative force (*p* < 0.001, mean difference: −0.43 N/kg, ES: −0.777) and absolute (*p* = 0.006, mean difference: −299 N.s, ES: −0.563) and relative impulse (*p* = 0.007, mean difference: –3.23 N.s/kg, ES: −0.551). Practitioners working with Argentinean amateur rugby players should consider these findings to reduce reinjury risk in players with previous hamstring injuries.

**Keywords:** injury prevention; hamstring injuries; eccentric strength

**Funding:** This research received no external funding.

**References**
  1. Tondelli, E., Boerio, C., Andreu, M., & Antinori, S. (2022). Impact, incidence and prevalence of musculoskeletal injuries in senior amateur male rugby: epidemiological study. *Physician and Sportsmedicine*, *50*(3), 269–275. https://doi.org/10.1080/00913847.2021.1924045
  2. Fyfe, J. J., Opar, D. A., Williams, M. D., & Shield, A. J. (2013). The role of neuromuscular inhibition in hamstring strain injury recurrence. In *Journal of Electromyography and Kinesiology* (Vol. 23, Issue 3, pp. 523–530). https://doi.org/10.1016/j.jelekin.2012.12.006
  3. Charlton, P. C., Raysmith, B., Wollin, M., Rice, S., Purdam, C., Clark, R. A., & Drew, M. K. (2018). Knee flexion not hip extension strength is persistently reduced following hamstring strain injury in Australian Football athletes: Implications for Periodic Health Examinations. *Journal of Science and Medicine in Sport*, *21*(10), 999–1003. https://doi.org/10.1016/j.jsams.2018.03.014
  4. Timmins, R. G., Bourne, M. N., Shield, A. J., Williams, M. D., Lorenzen, C., & Opar, D. A. (2016). Short biceps femoris fascicles and eccentric knee flexor weakness increase the risk of hamstring injury in elite football (soccer): A prospective cohort study. *British Journal of Sports Medicine*, *50*(24), 1524–1535. https://doi.org/10.1136/bjsports-2015-095362

### 2.70. Strength Power–Velocity Modifications in Young Cross Country Skiers After a Training and Competitive Season

Jesús Torres ^1,^* and Aitor Pinedo-Jauregi ^2^

^1^  Department of Physical Education and Sport, Faculty of Education and Sport, University of Basque Country (UPV/EHU), Vitoria-Gasteiz, 01007, Spain.; jtorres009@ikasle.ehu.eus
^2^  Society, Sports, and Exercise Research Group (GIKAFIT), Department of Physical Education and Sport, Faculty of Education and Sport, University of the Basque Country UPV-EHU, Vitoria-Gasteiz, Spain; Bioaraba, Vitoria-Gasteiz, Spain

* Correspondence: jtorres000@ikasle.ehu.eus; Tel.: +34-652913713

**Abstract:** Cross Country (XC) Ski training and competition involve diverse physiological demands; in the case of strength and power–velocity training (SP-V), a positive effect on XC skiers’ performance has been found before [2]. The aim of this study was to compare SP-V performance between the training and competitive periods (T&CS) and analyze the correlation between handgrip and the rest of the variables. Six male and one female XC skier (16.1 ± 3.0 yr) with more than 2 years of experience in XC ski training participated in the study. Two laboratory tests were performed; the first test (PreCs) was performed before the beginning of the competitive season, and the second test was performed at the end of the competition season (PosCs). The following exercises were analyzed: countermovement Jump (CMJ), handgrip strength (HandS), maximal load in a squat (1RMS) and bench press (1RMP), and speed of 1 m/s in Squat (S1M/S) and bench press (P1M/S). The velocity of load displacement was measured using a rotary encoder, and velocity and maximum repetition (MR) were estimated using interpolation [1]. In T&CS, training consists of ski roller sessions, specific sessions on snow, and SP-V training. To analyze statistical differences, a T-test was performed, and normality was checked using the Shapiro–Wilk test. The results showed statistically significant differences (*p* < 0.05) after T&CS for all variables analyzed, except CMJ. HandS improved from 42.55 to 46.92 Nm. Weight lifted at 1 m/s improved from 63.17 kg to 75.50 kg in Squat and from 24.83 to 27.33 kg in the bench press. In the case of MR, improvements in squat (63.17 kg vs. 75.50 kg) and bench press (51 kg vs. 56.50 kg) were observed. Correlation analyses showed that HandS was significantly correlated with 1RMS, S1M/S, 1RMP, and P1M/S; 1RMP was correlated with 1RMS and S1M/S; and P1M/S was correlated with 1RMS and S1M/S. Correlations were observed in both PreCs and PosCs. The research results suggest that SP-V performance improved at the end of the season; furthermore, the finding of a correlation between handgrip and strength testing suggests that it may be a new method for monitoring performance improvement during the season. This preliminary study opens the door for future research to confirm this, as it may be used in the future to test XC skiers’ performance throughout the XC ski season.

**Keywords:** cross-country ski; strength; force–velocity

**Funding:** This research received no external funding.

**References**
  1. Izquierdo, M., González-Badillo, J. J., Häkkinen, K., Ibáñez, J., Kraemer, W. J., Altadill, A., Eslava, J., & Gorostiaga, E. M. (2006). Effect of loading on unintentional lifting velocity declines during single sets of repetitions to failure during upper and lower extremity muscle actions. *International Journal of Sports Medicine*, *27*(9). https://doi.org/10.1055/s-2005-872825
  2. Stöggl, T., & Holmberg, H. C. (2022). A Systematic Review of the Effects of Strength and Power Training on Performance in Cross-Country Skiers. In *Journal of Sports Science and Medicine* (Vol. 21, Issue 4). https://doi.org/10.52082/jssm.2022.555

### 2.71. Stair-Climbing Versus Machine-Based Resistance Exercise to Improve Muscle Power in Older Adults

Evelien Van Roie ^1,^*, Jannique van Uffelen ^1^ and Christophe Delecluse ^1^

^1^  Physical Activity, Sports and Health Research Group, Department of Movement Sciences, KU Leuven. Leuven, Belgium; Jannique.vanuffelen@kuleuven.be, Christophe.delecluse@kuleuven.be

* Correspondence: Evelien.vanroie@kuleuven.be; Tel.: +3216-379431

**Abstract:** Machine-based resistance training (RT) can reduce age-related loss of muscle power (Pmax) [1]. However, weight-bearing exercises have a higher potential for large-scale implementation. This study investigated whether stair-climbing exercise (STAIR) was non-inferior for improving Pmax compared to machine-based RT in older adults. Functional capacity and cognition were included as secondary outcomes. Community-dwelling older adults (30♂, 16♀; 70.9 ± 4.3 years) were randomly assigned to RT or STAIR (n = 23/group). Supervised lab-based training sessions were performed twice weekly for 12 weeks. In weeks 1–4, exercises were performed at a controlled speed (hypertrophy-oriented; 4 × 12–15 repetitions; 55% of one-repetition maximum (1-RM) in RT; step-up exercise with height of 30–40 cm in STAIR), in weeks 5–12, exercises were performed as fast as possible (power-oriented, 4 × 12 repetitions; 40% of 1-RM for RT and 4 × 2 flights of 6 steps for STAIR; +10% load from week 9 onwards in both groups). Leg-extensor Pmax, functional capacity, and cognition were measured pre- and post-intervention, and Pmax was also measured after 4 weeks of training. Pmax tended to increase more in RT than in STAIR (19.5 ± 12.2% versus 13.7 ± 16.5%, d = 0.39, *p* = 0.086). Non-inferiority analyses of Pmax were inconclusive. All functional capacity tests showed a significant improvement over time (*p* < 0.05). STAIR increased more in stair-ascent performance (d = 0.45–0.61, *p* < 0.05) than RT, while similar improvements were found in both groups for the 10 m fast walk, 5-repetition sit-to-stand performance, and countermovement jump height (interaction effect: *p* > 0.05; time effect: *p* < 0.05). In contrast to the work of Yoon et al. on power training in older adults with cognitive frailty or mild cognitive impairment [2,3], neither RT nor STAIR induced significant gains in general cognitive performance and executive function in our cognitively healthy sample (*p* > 0.05). However, similar improvements in the digit span test forwards were observed in RT and STAIR (time effect: *p* = 0.004). In conclusion, STAIR and RT both induced significant changes in Pmax and functional capacity in older adults. Although RT tended to be superior in terms of gains in leg-extensor power, STAIR has a higher potential for implementation and might therefore be more effective in real life. Further research is necessary to investigate the optimal exercise dose and progression over a longer-term intervention and to examine the effects of STAIR when implemented in a home-based setting with limited expert oversight.

**Keywords:** bench stepping; cognition; force–velocity profile; functional capacity; strength training; weight-bearing exercise

**Funding:** E. Van Roie was supported by the Research Foundation Flanders, Belgium (senior postdoctoral fellowship 12Z5720N).

**References**
  1. Fragala, M.S., Cadore, E.L., Dorgo, S., Izquierdo, M., Kraemer, W.J., Peterson, M.D., Ryan, E.D. Resistance training for older adults: position statement from the National Strength and Conditioning Association. *J Strength Cond Res* **2019**, *33(8)*, 2019–2052.
  2. Yoon, D.H., Kang, D., Kim, H.J., Kim, J.S., Song, H.S., Song, W. Effect of elastic band-based high-speed power training on cognitive function, physical performance and muscle strength in older women with mild cognitive impairment. *Geriatr Gerontol Int* **2017**, *17(5)*, 765–772.
  3. Yoon, D.H., Lee, J.Y., Song, W. Effects of resistance exercise training on cognitive function and physical performance in cognitive frailty: a randomized controlled trial. *J Nutr Health Aging* **2018**, *22(8)*, 944–951.

### 2.72. Can Dietary Habits Influence Body Composition and Fitness in Elite Youth Athletes?

Antonio E. Vélez-Alcázar ^1^, Juan Alfonso García-Roca ^1,^* and Raquel Vaquero-Cristóbal ^2,^*

^1^  Facultad de Deporte, UCAM Universidad Católica de Murcia, 30310 Cartagena, Spain; aevelez@alu.ucam.edu
^2^  Department of Physical Activity and Sport, Faculty of Sport Sciences, University of Murcia, 30720 Murcia, Spain; raquel.vaquero@um.es

* Correspondence: jagarcia@ucam.edu (J.A.G.-R.); raquel.vaquero@um.es (R.V.-C.)

**Abstract:** Several studies have shown that dietary habits may influence body composition and physical performance in athletes. In Mediterranean countries, there is a dietary pattern known as the Mediterranean diet (MD), which is presented as a rich and healthy nutritional pattern. However, there are contradictory results on whether an adequate MD can improve athletes’ performance, and no studies have analyzed this issue. Therefore, the aim of the present research study was to analyze whether adherence to the Mediterranean diet (AMD) could affect anthropometric variables, body composition, and physical fitness in young elite athletes. The sample was selected using a non-probabilistic method of cohabitation among athletes in the Federación de Atletismo de la Región de Murcia (FAMU), Spain. The inclusion criteria were (1) being an athlete in the U-16, U-18, or U-20 categories, and (2) being among the athletes selected for the technification programs of the Athletics Federation of the Region of Murcia and/or competing in the First National Athletics Division. The selected sample consisted of a total of 96 adolescent athletes, of whom 47 were male (age = 18.31 ± 2.31 years) and 49 female (age = 17.27 ± 1.44 years). All athletes self-completed the KIDMED questionnaire to ascertain their AMD, classifying them into poor, medium, and excellent adherence to MD. They also underwent an anthropometric assessment to estimate their body composition. Finally, a physical fitness assessment was carried out, including tests to assess hamstring extensibility, stability, vertical and horizontal jumping power, grip strength, medicine ball throwing, and 30 m sprint. No differences were found as a function of AMD in body mass index (*p* = 0.893), ∑3 Skinfolds (*p* = 0.120), ∑ corrected girths (*p* = 0.146), fat mass (%) (*p* = 0.194), adipose tissue (%) (*p* = 0.057), muscle mass (%) (*p* = 0.313), bone mass (%) (*p* = 0. 814), muscle/bone index (*p* = 0.301), lower limb length (*p* = 0.996), right handgrip (*p* = 0.240), left handgrip (*p* = 0.193), sit-and-reach test (*p* = 0.487), CMJ (*p* = 0.410), SJ (*p* = 0.213), horizontal jump test (*p* = 0.899), medicine ball throw (*p* = 0.381), and 30 m sprint (*p* = 0.326). In conclusion, AMD does not seem to improve the levels of physical fitness, anthropometric parameters, and body composition in adolescents and young high-level athletes.

**Keywords:** Mediterranean diet; athlete; adherence; anthropometry; physical fitness.

**Funding:** This research received no external funding.

**References**
  1. Calella, P.; Gallè, F.; Cerullo, G.; Postiglione, N.; Ricchiuti, R.; Liguori, G.; D’Angelo, S.; Valerio, G. Adherence to Mediterranean Diet among Athletes Participating at the XXX Summer Universiade. Nutr. Health 2023, 29, 645–651.
  2. García-Hermoso, A.; Ezzatvar, Y.; López-Gil, J.F.; Ramírez-Vélez, R.; Olloquequi, J.; Izquierdo, M. Is Adherence to the Mediter- ranean Diet Associated with Healthy Habits and Physical Fitness? A Systematic Review and Meta-Analysis Including 565Â 421 Youths. Br. J. Nutr. 2022, 128, 1433–1444.
  3. De Santi, M.; Callari, F.; Brandi, G.; Toscano, R.V.; Scarlata, L.; Amagliani, G.; Schiavano, G.F. Mediterranean Diet Adherence and Weight Status among Sicilian Middle School Adolescents. Int. J. Food Sci. Nutr. 2020, 71, 1010–1018.

### 2.73. Effect of Very Short-Term Jump Training Based on Force–Velocity Profile on Jump Performance

Amilton Vieira ^1,^* and Martim Bottaro ^2^

^1^  Strength Training Laboratory, University of Brasilia; amiltonvieira@unb.br
^2^  Strength Training Laboratory, University of Brasilia; martim@unb.br

* Correspondence: amiltonvieira@unb.br

**Abstract:** Strength training is of paramount importance for physical development and injury prevention. However, in sports, it is challenging to fit strength workouts into an athlete’s usually congested agenda; therefore, identifying more “efficient” strength training strategies is highly desirable. Studies [1,2] have shown that training programs based on the force–velocity (*Fv*) profile are more efficient, promoting a 7 to 17% improvement in jump height compared to the 2% improvement of a traditional training program. Others [3,4] point out that two to four training sessions, or “very short-term” training programs, can improve aspects of performance. In the present study, we investigated the effectiveness of four jump training sessions, based on the *Fv* profile, on the jump performance of athletes. Participants (n = 27) were randomly allocated into training or control groups. Pre- and post-measures (2 weeks) included vertical jumps with and without overloads (0, 25, 50, 75, and 100% of body mass). Jumps were performed over a force plate, allowing for *Fv* profiling and determination of other mechanical jump metrics. The training group performed four jump training sessions over two weeks (twice a week, at least 48 h apart). Jump training included eight sets of five jumps, according to the individual *Fv* profile (i.e., heavy 75–100% versus light 0–25%). The participants allocated to the control group kept their specific sports training routine. An unpaired *t*-test was used for between-group comparisons. Training promoted a 3–4% increase in all jump metrics compared to the control group; however, only the jump height reached statistical significance, improving by 3.1% (95% CI: 0.1, 6.5, *p* = 0.046). RSImod increased by 4.1% (95% CI: −1.4, 8.8, *p* = 0.075), CM depth by 4.4% (95% CI: −1.2, 10.4, *p* = 0.166), and time to takeoff by 2.6% (95% CI: −1.9, 7.5, *p* = 0.299). The present study demonstrated that the four jump training sessions, based on the *Fv* profile, are an effective strategy for improving jump height by 3%. This result seems pertinent for athletes seeking a quick strategy to boost performance.

**Keywords:** exercise prescription; strength training; vertical jump.

**Funding:** This research was funded by Fundação de Apoio à Pesquisa do Distrito Federal (FAPDF).

**References**
  1. Jiménez-Reyes, P. et al. Effectiveness of an individualized training based on force-velocity profiling during jumping. *Front Physiol* **2016**, *7*, 677.
  2. Jiménez-Reyes, P. et al. Optimized training for jumping performance using the force-velocity imbalance: Individual adaptation kinetics. *PLoS One* **2019**, 14, e0216681.
  3. Cunha, R. et al. Effects of short-term isokinetic training with reciprocal knee extensors agonist and antagonist muscle actions: a controlled and randomized trial. *Braz J Phys Ther* **2013**, 17, 137–145.
  4. Archer, A. et al. Effects of short-term jump squat training with and without chains on strength and power in recreational lifters. *IJKSS* 2016, 4, 18–24.

### 2.74. Measured Force Plate Versus Estimated Force–Velocity–Power Profile: Is There Agreement Between Training Diagnosis?

Amilton Vieira ^1^, Lucas Ugliara ^1^, Davi Domingues ^1,^* and Martim Bottaro ^1^

^1^  Strength and Conditioning Laboratory, Faculty of Physical Education, University of Brasilia (UnB), Brasilia, Brazil; A.V., amiltonvieira@unb.br; L.U., lucasugliaraunb@gmail.com; D.D., davi.domingues@aluno.unb.br; M.B., martim@unb.br

* Correspondence: davi.domingues@aluno.unb.br; Tel.: +55-(61)-98149-1904

**Abstract:** Nobel laureate AV Hill demonstrated that the force–velocity relationship of concentric elbow flexions presents a linear function with a negative slope [1], meaning that the capacity to generate force decreases as muscle group shortening velocity increases. There is still growing interest in investigating the principles of this relationship, which may be used to evaluate athletes’ performance and guide training prescription [2]. Samozino et al. [3]. developed a method for estimating force, velocity, and power parameters during the squat jump test, requiring relatively simple measurements to estimate the force–velocity–power (FVP) profile, such as jump height, push-off distance, and body mass. Samozino’s method has been used in various studies, but its validity has also been questioned [4]. We investigated the level of agreement between actual and estimated FVP diagnosis (i.e., force deficit, velocity deficit, or balanced). We hypothesize that appropriate methodological control may result in trivial-to-small errors in FVP metric estimation, thereby not affecting the estimated diagnostics. After familiarizations, 40 athletes (26 ± 6 years) performed a loaded squat jump protocol with 0, 25, 50, 75, and 100% of their body mass on a force plate at 1000 Hz. The FVP profile was obtained from both the force–time data and simple inputs (i.e., body mass, jump height, and push-off distance) following Samozino’s method. Cohen’s Kappa (*k*) was used to assess the agreement between actual and estimated FVP parameters (i.e., balanced, force deficit, velocity deficit), while Student’s paired *t*-test was used for comparisons. Our analysis showed a lack of agreement between the actual and estimated parameters (*k* = 0.13, CI95% 0.03 to 0.30, *p* = 0.12). The comparisons showed that actual maximal force and force–velocity slope were significantly greater than the estimated parameters, while actual maximal velocity and power were significantly lower than the estimated parameters (*p* < 0.01, ES > 0.8). The disagreement between actual and estimated FVP parameters does not confirm the validity of Samozino’s method. Although the force–velocity profile can be easily estimated by simple devices like a slow-motion camera or a contact mat, practitioners should consider that even minor errors in these measures may be summed in each estimation step, thus dramatically reducing the validity of the estimated FVP diagnosis. Following our findings, coaches and athletes should perform the actual measures of force, velocity, and power to create the athlete’s profile rather than estimating these parameters.

**Keywords:** muscle strength; muscle power; force–velocity relationship; exercise prescription; vertical jump.

**Funding:** This study was supported by the Fundação de Apoio à Pesquisa do Distrito Federal (FAPDF).

**References**
  1. Hill, A.V.; The maximum work and mechanical efficiency of human muscles, and their most economical speed. *J Physiol* **1922**, *56*, 19–41.
  2. Jiménez-Reyes, P. et al.; Effectiveness of an Individualized Training Based on Force-Velocity Profiling during Jumping. *Front Physiol* **2017**, 7.
  3. Samozino, P. et al.; A simple method for measuring force, velocity and power output during squat jump. *J Biomech* **2008**, *41*, 2940–2945.
  4. Hicks, D.S. et al.; Measurement agreement between Samozino’s method and force plate force-velocity profiles during barbell and hexbar countermovement jumps. *J Strength Cond Res* **2022**, *36*, 3290–3300.

### 2.75. Reticulospinal and Corticospinal Responses in Long-Term Strength-Trained and Untrained Individuals

Simon Walker ^1,^*, Meghan Tanel ^1^, Dawson Kidgell ^2^, Sakari Vekki ^1^, Eeli Halonen ^1^, Gonzalo Gomez Guerrero ^1^, Juha Ahtiainen ^1^ and Stuart N Baker ^3^

^1^  NeuroMuscular Research Center, Faculty of Sport and Health Sciences, University of Jyväskylä, Jyväskylä, Finland; simon.walker@jyu.fi
^2^  Monash University Exercise Neuroplasticity Research Unit, School of Primary and Allied Health Care, Monash University, Melbourne, Australia; dawson.kidgell@monash.edu
^3^  Institute of Biosciences, Faculty of Medical Sciences, Newcastle University, Newcastle upon Tyne, United Kingdom; stuart.baker@newcastle.ac.uk

* Correspondence: simon.walker@jyu.fi

**Abstract:** The corticospinal tract has been a primary target for investigating neural adaptations to resistance training. However, given their evolutionary roles, it could be argued that the reticulospinal tract is better suited to underpin the known resistance training-induced increases in motor unit recruitment and discharge rate. Here, a battery of electrophysiological tests was employed to assess cortico and reticulospinal excitability via motor-evoked potential (MEP) amplitude in 15 (11 M/4 F, ~32 y) competitive strength-trained athletes compared to 18 (11 M/7 F, ~32 y) untrained age- and sex-matched controls. Subjects performed maximal unilateral isometric elbow flexion contractions (MVCs), followed by the StartReact test described by [1]. Thereafter, transcranial magnetic stimulation (TMS) targeting the right biceps brachii was used to determine active motor threshold (aMT), assess the PA–current recruitment curve over 100–180% aMT, compare AP–current to PA–current response at 120% aMT, and trigger 50 ms after startle-inducing loud sound (120 dB) at 120% aMT. All TMS responses were normalized to maximal compound action potential (M-max). Strength-trained athletes had greater MVCs (~122 vs. ~84 N·m, *p* = 0.003) and rate of torque development over 0–50 ms (~412 vs. ~263 N·m/s, *p* = 0.016) compared to untrained controls. Startling sound had a lower impact on strength-trained athletes, such that the ratio gain in reaction time was lower than for untrained controls (~1.85 vs. ~2.53, *p* = 0.003). The area under the recruitment curve over 140–180% aMT was greater in strength-trained athletes compared to untrained controls (~2.24 vs. ~1.16 arb.unit, *p* = 0.048), but this was abolished when assessing MEP area over the initial 5 ms (~0.45 vs. ~0.23 arb. unit, *p* = 0.118). AP–current response was enhanced compared to PA–current response in strength-trained athletes (~0.32 vs. ~0.42 MEP area/M-max, *p* = 0.007), but there was no difference in untrained controls (~0.26 vs. ~0.28 MEP area/M-max, *p* = 0.625). No differences were observed in the responses to TMS combined with the startle-inducing sound between the groups. TMS recruitment curve and AP–current responses showed, specifically, that late I-wave excitability was enhanced in strength-trained athletes, whereas there was no difference in early I-wave responses (i.e., 100–120% aMT and <5 ms MEP area). Further, the use of loud, startle-inducing sound, previously shown to specifically discharge reticulospinal tract neurons, had a greater effect on untrained subjects, suggesting that the athletes were already engaging this tract in their voluntary response. Overall, results suggest enhanced reticulospinal but not corticospinal functioning in long-term strength-trained athletes.

**Keywords:** transcranial magnetic stimulation (TMS); StartReact; neurophysiology; neural adaptation

**Funding:** This research was funded by the Research Council of Finland, grant number 350528

**References**
  1. Valls-Sole, J.; Sole, A.; Valldeoriola, F.; Munoz, E., Gonzalez, L.E., Tolosa, E.S. Reaction time and acoustic startle in human subjects. Neurosci Lett 1995, 195, 97–100.

### 2.76. The Effect of Psychological Stress on Heart Rate During Exercise

Ľubica Žiška Böhmerová ^1,^*, Veronika Pindrochová ^2^ and Dušan Hamar ^3^

^1^  Faculty of Physical Education and Sport, Comenius University Bratislava, Slovakia; lubica.bohmerova@uniba.sk
^2^  Faculty of Physical Education and Sport, Comenius University Bratislava, Slovakia; pindrochova3@uniba.sk
^3^  Faculty of Physical Education and Sport, Comenius University Bratislava, Slovakia; dusan.hamar@uniba.sk

* Correspondence: lubica.bohmerova@uniba.sk; Tel.: +421-903-305-022

**Abstract:** The physical load involves a sudden increase in energy requirements that activates the sympathetic system. We were curious about adding another cognitive stressor to this load. The study aimed to evaluate the impact of psychological stress on heart rate during physical exercises of various intensities. Ten young female volunteers participated in the study. The participants’ average age, height, and weight were 23.8 ± 1.0 years, 167.4 ± 6.1 cm, and 59.7 ± 5.5 kg, respectively. The exercise was conducted using a universal cycle ergometer in revolution-independent constant power mode. Individuals performed 3 min exercise loads at intensities of 1 W/kg (light zone), 2 W/kg (medium zone), and 3 W/kg (heavy zone), with 3 min breaks. This procedure was carried out once without and then with psychological stress in the form of the Stroop test. The participants determined whether the written color matched the actual color (e.g., “green” written in red). Images were presented in random order. Heart rate was monitored using a Garmin chest belt connected via Bluetooth to the Cardio Mez application. The order of the test (with or without stress) was assigned in random order. Average heart rate values from the final 2 min of the 3 min exercise periods were used for evaluation. Paired *t*-test and “Cohen’s d” were employed for statistical analyses. The results revealed significantly higher heart rate values (*p* ≤ 0.05) during the Stroop test at rest (85.2 vs. 76.5 bpm), as well as at 1 W/kg (123.6 vs. 116.9 bpm), 2 W/kg (133.9 vs. 127.2 bpm), and 3 W/kg (160.0 vs. 153.3 bpm). Additionally, “Cohen’s d” (d = 0.2) showed only a small effect. It seems that additional psychological stress in the form of the Stroop test further stimulates an increase in the heart rate not only at rest, but also during light, medium, and heavy-intensity exercise.

**Keywords:** physical load; psychological stress; heart rate

**Funding:** This research was supported by VEGA (Grant No. 1/0393/23).

**References**
  1. Bernaciková, M., J. Novotný, I. Burešová, A. Žákovská, 2019. Complex approach to monitoring athletes in the scope of overtraining prevention. In: Journal Studia Sportiva [online]. May 2021; 13(1), 17–26. [cit. 2021-5-11]
  2. Johnson, U., A. Ivarsson, 2017. Psychosocial factors and sport injuries: prediction, prevention and future research directions. In: Curr Opin Psychol. [online]. April 2017; 16, 89–92. [cit. 2017-04-26].
  3. Roche. F. et al., 2024. Anatomy and physiology of the autonomic nervous system: Implication on the choice of diagnostic/monitoring tools in 2023. In: Rev Neurol (Paris). January 2024; 180(1–2), 42–52.

### 2.77. Effects of Multicomponent Training and a Detraining Period on Cognitive and Functional Performance of Older Adults at Risk of Frailty

Ana Moradell ^1,2,3^, Isabel Iguacel ^1,4,5,6^, David Navarrete ^1,2,4,6^, Ángel-Iván Fernández García ^1,2^, Marcela González-Gross ^7,5^, Jorge Pérez-Gómez ^8^, Ignacio Ara ^9^, José Antonio Casajús ^1,2,4,5,10^, Alba Gómez-Cabello ^1,2,11^ and Germán Vicente-Rodríguez ^1,2,4,5,12,^*

^1^  Growth, Exercise, Nutrition and Development (EXER-GENUD) Research Group, Universidad de Zaragoza, Zaragoza, Spain; amoradell@unizar.es; iguacel@unizar.es; dnavarrete@unizar.es; angelivanfg@unizar.es; joseant@unizar.es; agomez@unizar.es
^2^  Exercise and Health in Special Population Spanish Research Net (EXERNET)
^3^  Instituto de investigación Sanitaria de Aragón (IIS Aragón), 50009, Zaragoza, Spain
^4^  Instituto Agroalimentario de Aragón-IA2 (Universidad de Zaragoza-CITA), 50090, Zaragoza, Spain
^5^  Centro de Investigación Biomédica en Red de Fisiopatología de la Obesidad y Nutrición (CIBEROBN), Instituto de Salud Carlos III, 28040 Madrid, Spain
^6^  Department of Physiatry and Nursing, Faculty of Health, University of Zaragoza, 50009 Zaragoza, Spain
^7^  ImFINE Research Group, Universidad Politécnica de Madrid, 28040 Madrid, Spain marcela.gonza-lez.gross@upm.es
^8^  HEME Research Group, University of Extremadura, 10003 Cáceres, Spain jorgepg100@unex.es
^9^  GENUD-Toledo Research Group, Universidad de Castilla-La Mancha, 45071 Toledo, Spain ignacio.ara@uclm.es
^10^  Department of Physiatry and Nursing, Faculty of Medicine, University of Zaragoza, 50009, Zaragoza, Spain
^11^  Centro Universitario de la Defensa, Zaragoza, Spain
^12^  Department of Physiatry and Nursing, Faculty of Health and Sport Sciences, University of Zaragoza, 50009, Zaragoza, Spain

* Correspondence: gervicen@unizar.es

**Abstract:** Similar to physical function, cognitive function deteriorates with ageing. The physical and mental alterations can be assessed using dual-task tests consisting of performing an activity that combines motor tasks with cognitive tasks [1]. Cognitive impairment is associated with the performance of daily activities and is thus a good predictor of future fractures [2]. The aim of this report was to analyze the effects of a 6-month multicomponent exercise program (MCT) and a 4-month detraining period on the functional and cognitive statuses of pre-frail and frail older people. A total of 108 frail and pre-frail individuals over 65 years of age participated. The sample was divided by convenience into a control group (CG) and an intervention group (IG) that underwent a 6-month MCT program followed by a 4-month detraining period. The 71 participants who completed all three assessments were included in the analysis. A dual task Time-Up and Go test, the Mini-Mental State Examination (MMSE), and basic and instrumental daily activities were evaluated. The three assessments were compared using repeated measures ANOVA. The results showed group-by-time interactions between CG and IG (*p* < 0.05). IG improved the performance of dual tasks after the 6-month training (13.7 ± 7.5 vs. 9.9 ± 4.7 s; *p* < 0.05), while performance declined to 11.2 ± 6.1 s after the detraining period, although the results obtained after the detraining period remained significantly higher than the baseline values (13.7 ± 7.5 vs. 11.2 ± 6.1 s; *p* < 0.05). Meanwhile, no changes were observed in CG from baseline (12.0 ± 4.3 s) to the 6-month training (12.0 ± 5.8 s), to detraining (11.6 ± 5.1 s). No significant differences were observed based on either the MMSE or the daily activities questionnaires. MCT had simultaneous beneficial effects on functional and cognitive performance in frail and pre-frail older adults without cognitive impairment. However, improvements in dual tasks may not be transferred to daily life activities or cognitive status alone. Although a 4-month detraining period had a negative impact on dual-task performance, the results remained higher than the baseline values; thus, based on the observed declines in detraining, ongoing exercise programs or shorter break periods are recommended.

**Keywords:** ageing; frailty; functional capacity; cognitive capacity; quality of life; multicomponent training; dual task

**Funding:** This study was funded by “Ministerio de Economía, Industria y Competitividad” (DEP2016-78309-R), “Centro Universitario de la Defensa de Zaragoza” (UZCUD2017-BIO-01),” CI-BER -Consorcio Centro de Investigación Biomédica en Red-“ (CB16/10/00477) and (CB12/03/30038), “Instituto de Salud Carlos III, Ministerio de Ciencia e Innovación and Unión Europea—European Regional Development Fund”, “Universidad de Zaragoza, Programa Propio de Investigación del Vicerrectorado de Política Científica, Proyectos Puente 2022” (UZ2021-BIO-05) and “Consejo Supe-rior de Deportes, Ministerio de Cultura y Deporte, Plan de Recuperación Transformación y Resliencia, Next Generation European Funds” (EXP_75079).3

**References**
  1. Verhaeghen, P.; Steitz, D.; Sliwinski, M.; Cerella, J. Aging and Dual-Task Performance: A Meta-Analysis. Psychol Aging 2003, 18, 443–460, doi:10.1037/0882-7974.18.3.443]
  2. Montero-Odasso, M.; Almeida, Q.J.; Bherer, L.; Burhan, A.M.; Camicioli, R.; Doyon, J.; Fraser, S.; Muir-Hunter, S.; Li, K.Z.H.; Liu-Ambrose, T.; et al. Consensus on Shared Measures of Mobility and Cognition: From the Canadian Consortium on Neuro-degeneration in Aging (CCNA). J Gerontol A Biol Sci Med Sci 2019, 74, 897–909, doi:10.1093/GERONA/GLY148

### 2.78. Influence of Technical and Medical Staff Structures on Injuries in Elite Professional Football

Silvia Garay ^1^ and Laura Nieto ^1,^*

^1^  UCAM 1; sgaray@ucam.edu, lnieto@ucam.edu

* Correspondence: sgaray@ucam.edu

**Abstract:** The incidence of football injuries has risen due to increased physical demands [1]. Decisions regarding the technical and medical staff structure significantly influence team performance. This study had a dual objective: (I) describing the structures of technical and medical staff, and (II) analyzing how these structures impact injuries and performance. To conduct this study, we examined the top 10 teams in La Liga, the Premier League, and the Bundesliga at the end of the 2021/2022 season. Data were gathered from Transfermarkt.com and the clubs’ official websites. Multiple linear regression models were employed to analyze relationships between independent variables (physical trainer, re-adapter, performance manager, medical director, doctor, medical team, physiotherapist, nutritionist, psychologist, and nurse) and dependent variables (total injuries, muscle injuries, joint injuries, contusions, fractures, inflammation, player availability, and season-ending points). Results showed a direct correlation between muscle injuries and the number of physical trainers (*p* = 0.096), indicating that an increase in the number of physical trainers implies an increase in muscle injuries. Additionally, a significant inverse relationship was found between the number of medical team members and muscle injuries (*p* = 0.084), suggesting that more doctors and medical chiefs correlate with fewer muscle injuries. Joint injuries were inversely correlated with the number of nurses (*p* = 0.04), meaning an increase in nurses led to a decrease in joint injuries. Furthermore, concussions (*p* = 0.056) and inflammations (*p* = 0.083) showed positive relationships with the number of re-adapters—more re-adapters were correlated with higher occurrences of concussions and inflammations. Fractures were also positively correlated with performance managers (*p* = 0.046). Lastly, physiotherapists (*p* = 0.001) and nutritionists (*p* = 0.008) were positively associated with points earned by the season’s end. In conclusion, the relationship between the structure of technical and medical staff in professional football and injury incidence is crucial in sports management. Analysis of the top 10 teams in La Liga, the Premier League, and the Bundesliga in 2021/2022 reveals significant impacts on injury rates and athletic performance. Notably, more physical trainers were correlated with increased injuries, which contrasts with expectations. This may be due to intensified training leading to player fatigue [2] or operational differences hindering personalized training. Excessive supervision could also contribute to player stress [3]. Sports managers must make informed decisions to balance training, recovery, and injury prevention effectively and sustainably.

**Keywords:** professional football; management; technical staff; medical staff; injuries; availability; LaLiga; Premier League; Bundesliga

**Funding:** This research received no external funding.

**References**
  1. Ekstrand, J, Hägglund, M., & Waldén, M. (2011). Epidemiology of muscle injuries in professional foot- ball (soccer). American Journal of Sports Medicine, 39(6), 1226–1232.
  2. Gabbett, T. J. (2016). The training—injury prevention paradox: should athletes be training smarter and harder?. British journal of sports medicine, 50(5), 273–280.
  3. Kellmann, M. (2010). Preventing overtraining in athletes in high-intensity sports and stress/recovery monitoring. Scandinavian Journal of Medicine & Science in Sports, 20(s2), 95–102. https://doi.org/10.1111/j.1600-0838.2010.01192.x

### 2.79. Reliability of the Force–Velocity Relationship and Its Association with Physical and Cognitive Function in Institutionalized Older Adults

Héctor Gutiérrez-Reguero ^1,2,3,4^, Javier Leal-Martín ^1,2,3,4,5^, Iván Baltasar-Fernandez ^1,2,3,4^, Ángel Buendía-Romero ^1,2,3,4, 5^ Álvaro Rosado ^6^, Luis M. Alegre ^1,2,3,4^, Ignacio Ara ^1,2,3,4^, Francisco J. García-García ^2,3,4,7^ and Julian Alcazar ^1,2,3,4,^*

^1^  GENUD Toledo Research Group, Faculty of Sports Sciences, University of Castilla-La Mancha, Toledo, 7 Spain
^2^  CIBER on Frailty and Healthy Aging (CIFERFES), Instituto de Salud Carlos III, Madrid, Spain
^3^  Grupo Mixto de Fragilidad y Envejecimiento Exitoso UCLM-SESCAM, Universidad de Castilla-La Mancha-Servicio de Salud de Castilla-La Mancha, Toledo, Spain 11
^4^  Instituto de Investigación Sanitaria de Castilla-La Mancha (IDISCAM), Junta de Comunidades de Castilla-La Mancha (JCCM), Toledo, Spain
^5^  Faculty of Health Sciences, University of Castilla-La Mancha, Toledo. Spain
^6^  Residencia para Personas Mayores Barber de Toledo, Toledo, Spain
^7^  Geriatric Department, Complejo Hospitalario Universitario de Toledo, Toledo, Spain

* Correspondence: julian.alcazar@uclm.es

**Abstract:** The force–velocity (F-V) relationship can be used to assess an individual’s neuromuscular performance and prescribe training intensity, making it a highly valuable tool for trainers in designing tailored training programs (1). The F-V relationship has been proven to be reliable in both athletic populations and older adults (2,3). However, it is still not known whether this tool can be used reliably in older adults living in nursing homes, who may require better control of training intensities due to their prevalent clinical conditions. Therefore, the main objective of this study was to assess the inter-day reliability of the F-V relationship and its association with physical and cognitive function in institutionalized older adults. Twenty-seven nursing home residents (82.9 ± 8.7 years, 63% women) completed four testing sessions over two weeks, separated by 48 h. In the first week, two sessions were conducted to evaluate physical (Short Physical Performance Battery Test) and cognitive (Montreal Cognitive Assessment) functions and to familiarize the participants with the leg press exercise. In the second week, two incremental loaded tests were conducted on the leg press over two different days, both with the same number of loads and absolute intensity (kg). After a warm-up, the participants performed a sequence of sets with increasing loads, performing 2 to 4 repetitions with the intention of lifting the load as fast as possible. A short rest (≈2–3 s) was allowed between repetitions with the same load, and a moderate rest (60–120 s) was allowed between sets with different loads. A linear regression was fitted to collect mean force and velocity values to extrapolate the following F-V variables: estimated maximum isometric force (F0), maximal unloaded velocity (V0), maximum power (Pmax), and the slope of the F-V profile (FVslope). The F-V test ended when the participants reached a load equivalent to ~75% of F0. There were no significant differences between F-V outcomes collected in the two different F-V tests (all *p* > 0.05). Between-day correlations for all variables ranged from Pearson’s r = 0.66 to 0.96 (all *p* < 0.001). Intraclass correlation coefficient values were 0.96 for F0, 0.77 for V0, 0.98 for Pmax, and 0.84 for FVslope (all *p* < 0.001), and the coefficient of variation ranged from 5.5% to 14.9%. Lastly, there was no association between the test–retest differences for all F-V variables and physical and cognitive functions (all *p* > 0.05). In conclusion, the use of the F-V relationship was reliable in institutionalized older adults, independent of physical or cognitive performance.

**Keywords:** aging; F-V profile; strength training; reproducibility; velocity-based training

**Funding:** This work was supported by CIBER—Consorcio Centro de Investigación Biomédica en Red—(CB16/10/00477 and CB16/10/00456), Instituto de Salud Carlos III, Ministerio de Ciencia e Innovación, and Unión Europea—European Regional Development Fund; Plan Propio de Investigación of the University of Castilla-La Mancha and FEDER funds from the European Union (2022-GRIN-34296); and by the Ministerio de Universidades of the Government of Spain (grant number FPU21/04717).

**References**
  1. Campos GE, Luecke TJ, Wendeln HK, Toma K, Hagerman FC, Murray TF, Ragg KE, Ratamess NA, Kraemer WJ, Staron RS. Muscular adaptations in response to three different resistance-training regimens: specificity of repetition maximum training zones. Eur J Appl Physiol. 2002 Nov;88(1–2):50–60. 55
  2. Alcazar J, Rodriguez-Lopez C, Ara I, Alfaro-Acha A, Mañas-Bote A, Guadalupe-Grau A, García-García FJ, Alegre LM. The Force-Velocity Relationship in Older People: Reliability and Validity of a Systematic Procedure. Int J Sports Med. 2017 Dec;38(14):1097–1104.
  3. Lindberg K, Solberg P, Bjørnsen T, Helland C, Rønnestad B, Thorsen Frank M, Haugen T, Østerås S, Kristoffersen M, Midttun M, Sæland F, Paulsen G. Force-velocity profiling in athletes: Reliability and agreement across methods. PLoS One. 2021 Feb 1;16(2):e0245791.

### 2.80. Interrelationships of Strength, Muscle Mass, and Muscle Quality in Knee Extensors of an Active Young Population

**Santos-Pérez, J. ^1^, González-Martín, D. ^1^, Sánchez-García N. ^1^, Reyes-Merino, F. ^1^, Hernández-Murua, J.A. ^2^ and Gallego-Selles, A. ^3^**

^1^  University of León: Campus Vegazana s/n 24071, León, Spain; jsantp@unileon.es
^2^  Escuela Superior de Educación Física, Universidad Autónoma de Sinaloa, Culiacán 80040, México
^3^  Department of Physical Education and Research Institute of Biomedical and Health Sciences (IUIBS), Uni versity of Las Palmas de Gran Canaria, Las Palmas de Gran Canaria, Spain

**Abstract:** Muscle quality, defined as the skeletal muscle’s ability to generate force relative to its mass, plays a critical role in physical performance and overall health. This dynamic relationship is influenced by various factors, including age, gender, and individual characteristics. This study investigated the interplay between muscle mass, strength, and quality in the knee extensors of active young individuals, focusing on potential asymmetries between lower limbs. Forty-three active university students (26 males and 17 females) were evaluated. Knee extensor strength was measured using a strain gauge (Chronojump Boscosystems) connected to a leg extension machine (BH Fitness). Muscle mass was assessed using a GE Lunar Prodigy^®^ densitometer, determining the thigh region of interest, including the pelvic area, for analysis. Maximum voluntary isometric contraction (MVIC) was recorded both unilaterally and bilaterally (knee 90° flexion), and maximum repetition (1RM) was determined unilaterally in an incremental test with progressive loads. Data were analyzed using SPSS software, with significance set at *p* < 0.05, and the results were presented as mean ± SD. Significant strength and muscle mass differences were observed between genders (*p* < 0.05), although no significant differences were found after adjusting for muscle mass. Despite higher muscle mass in males (59.96 ± 6.93 kg vs. 41.17 ± 5.2 kg in females), muscle quality measured by bilateral MVIC was comparable between males (11.37 ± 2.15) and females (10.83 ± 1.7); similar results were obtained for muscle quality calculated unilaterally for 1RM. No differences were found in absolute and relative force asymmetry between genders; 25% of the sample showed bilateral deficits, a slightly lower percentage than described in the literature. Assessment of muscle quality is critical to understanding force-generating capacity in relation to muscle mass in active young people. Developing early intervention strategies could enhance athletic performance and promote long-term muscle health. Future research could use this protocol to explore muscle quality in populations at risk of sarcopenia or muscle weakness.

**Keywords:** muscle quality; physical performance; knee extensor strength

**Funding:** This research was funded by Ministerio de Ciencia e Innovación, grant number 35 PID2021-125354OB-C21. AGS is a beneficiary of the Catalina Ruiz training grant program for researchers from the Consejería de Economía, Conocimiento y Empleo, as well as the Fondo Social Europeo.

**References**
  1. Fragala, M. S.; Kenny, A. M.; Kuchel, G. A. Muscle quality in aging: a multi-dimensional approach to muscle functioning with applications for treatment. Sports Med. 2015, 45, 641–658. https://doi.org/10.1007/s40279-015-0305-z
  2. Kuschel, L. B.; Sonnenburg, D.; Engel, T. Factors of Muscle Quality and Determinants of Muscle Strength: A Systematic Literature Review. Healthcare 2022, 10, 1937. https://doi.org/10.3390/healthcare10101937
  3. Portilla-Cueto, K.; Medina-Pérez, C.; Romero-Pérez, E. M.; Núñez-Othón, G.; Horta-Gim, M. A.; de Paz, J. A. Muscle Quality of Knee Extensors Based on Several Types of Force in Multiple Sclerosis Patients with Varying Degrees of Disability. Medicina 2022, 58, 316. https://doi.org/10.3390/medicina58020316.
  4. Zhang, Y.; Chen, K.; Liu, K.; Wang, Q.; Ma, Y.; Pang, B.; Huang, L.; Ma, Y. New prediction equations for knee isokinetic strength in young and middle-aged non-athletes. BMC Public Health 2023, 23, 2558. https://doi.org/10.1186/s12889-023-17478-7.

### 2.81. Incidence and Economic Impact of Injuries in the Main European Leagues

**Laura Nieto ^1^ and Pedro E. Alcaraz ^2^**

^1^  UCAM 1; lnieto@ucam.edu
^2^  UCAM, CIARD 2; palcaraz@ucam.edu

* Correspondence: lnieto@ucam.edu

**Abstract:** Player availability is key for professional football clubs as it directly impacts the team’s performance. Additionally, not having a player available for selection in a match due to injury places an economic cost on the club. In this regard, [1] estimated that the cost of injuries to the Australian league ranges between AUD 187,990 and AUD 332,680. Eliakim et al. [2] estimated the cost to the Premier League to be around GBP 45 million, while Nieto et al. [4] estimated it to be EUR 45.2 million. These data highlight the importance of quantifying and preventing injuries in professional soccer. Therefore, the aims of this study were 1) to determine the total number of injuries, specifically muscular, fractures, joint, and contusion injuries, and 2) to analyze the economic impact of missed matches due to injuries. We performed a retrospective cross-sectional study for the 2021/2022 season of the Spanish (LaLiga), German (Bundesliga), and English (Premier League) professional football leagues. To estimate the economic cost, we considered the player’s non-availability (as a ratio of matches not played by the player divided by the total matches played by his specific team) and his salary. The total number of injuries in the 2021/2022 season ranged from 1202 (LaLiga) to 1399 (Bundesliga). The sum of missed matches was 2953 for Bundesliga, 3455 for Premier League, and 3030 for LaLiga. The most frequent injury was muscular tendonitis, with a relative weight over the total injuries that ranged from 28% (Bundesliga) to 38% (LaLiga). Joint injuries were the second most frequent type, with a relative weight of 17% in the Bundesliga and the Premier League, and 11% in LaLiga. Contusions were the third most common type of injury, with LaLiga having the lowest rate (7%) and the Bundesliga and the Premier League having 12% and 10% of contusions, respectively. The cost of all injuries was approximately EUR 109.8 M in the Bundesliga, EUR 264.9 M in the Premier League, and EUR 169.9 M in LaLiga. These data highlight the importance of creating programs for preventing muscle injuries and fostering interdisciplinary collaboration among highly skilled professionals. Establishing a well-defined framework and organized structure within the expert team, as well as delineating roles, regulations, and duties for each member, is crucial, as emphasized by Jukic et al. [3]. This approach is essential for mitigating the issue of excessive player absences and minimizing the considerable economic losses incurred by professional football clubs.

**Keywords:** injuries; professional soccer; prevention; player cost; player availability

**Funding:** This research was funded by Fundación Séneca, grant number 22134/PI/22.

**References**
  1. Lu, D., McCall, A., Jones, M., Steinweg, J., Gelis, L., Fransen, J., & Duffield, R. (2021). The financial and performance cost of injuries to teams in Australian professional soccer.J Sci Med Sport, 24(5), 463–467 44
  2. Eliakim, E., Morgulev, E., Lidor, R., & Meckel, Y. (2020). Estimation of injury costs: financial damage of English Premier League teams’ underachievement due to injuries. BMJ Open Sport & Exerci Med; 6(1), e000675
  3. Jukic, I.; Calleja-González, J.; Cuzzolin, F.; Sampaio, J.; Cos, F.; Milanovic, L.; Krakan, I.; Ostojic, S.; Olmo, J.; Requena, B.; et al. (2021). The 360◦ Performance System in Team Sports: Is It Time to Design a “Personalized Jacket” for Team Sports Players? Sports 9(3) 40.
  4. Nieto Torrejón L, Martínez-Serrano A, Villalón JM, Alcaraz PE. Economic impact of muscle injury rate and hamstring strain injuries in professional football clubs. Evidence from LaLiga. PLoS One. 2024 Jun 13;19(6):e0301498.

### 2.82. Acute and Delayed Responses in Women and Men with Similar Relative Strength

Luis Rodiles-Guerrero ^1,2,^*, Juan Sánchez-Valdepeñas ^2,4^, Pedro J. Cornejo-Daza ^1,2,3,4^, Miguel Sánchez-Moreno ^2,3,4^, Fernando Pareja-Blanco ^2,4^ and Beatriz Bachero-Mena ^1,2^

^1^  Department of Human Movement and Sport Performance, University of Seville, 41013 Seville, Spain
^2^  Science Based Training Research Group, Department of Sports and Computer Sciences, Universidad Pablo de Olavide, 41013 Seville, Spain
^3^  Department of Physical Education and Sports, University of Seville, 41013 Seville, Spain
^4^  Faculty of Sport Sciences, Department of Sports and Computer Sciences, Universidad Pablo de Olavide, 41013 Seville, Spain

* Correspondence: lrodiles@us.es

**Abstract:** Sex differences in muscle fiber types [1], neuromuscular characteristics [2], and load–velocity relationships [3] may explain the greater muscle strength reported in men compared to women, as well as the greater muscle endurance observed in women compared to men during some resistance training (RT) tasks. However, whether these sex-related strength differences are maintained when the relative strength [1-repetition maximum (1RM)/body weight] is equalized between sexes remains unknown. This study uses a cross-sectional design to compare the acute mechanical and metabolic responses and the temporal recovery course in women and men with similar relative strength after an RT session that comprised the full squat (SQ) exercise. Twenty-nine participants (14 women and 15 men) performed a single RT bout (load: 70% 1RM, sets: 3, velocity loss: 40%, inter-set rest: 3 min). Participants underwent a battery of tests at different time points: pre-exercise (Pre), post-exercise (Post), 10 min post-exercise (Post-10), and 24 h post-exercise (Post-24). Vertical countermovement jump (CMJ) height and the mean propulsive velocity (MPV) that corresponded to an absolute load of 60% 1RM in the SQ exercise (Load-60) were measured at the different time points. Blood lactate concentration was measured at Pre, Post, and Post-10. The total repetitions displayed a tendency toward a significant “Group × time” interaction (*p* = 0.09). A significant “Time” effect was reported for total repetitions (*p* = 0.001). In this regard, women completed significantly more repetitions in set 1 (9.6 ± 3.0 vs. 7.7 vs. 2.1; *p* = 0.05), set 2 (8.9 ± 3.1 vs. 6.2 ± 1.7; *p* = 0.007), and during the entire session (25.8 ± 6.6 vs. 20.2 ± 5.5; *p* = 0.020) than men. Regarding metabolic and mechanical responses, a significant “Group × time” interaction was observed for lactate concentration (*p* = 0.04), with higher lactate concentration at Post (*p* = 0.03) and Post-10 (*p* = 0.02) in women compared to men. For CMJ, a significant “Time” effect (*p* = 0.009) was reported, displaying similar impairments in both groups. Regarding Load-60, women and men showed a similar decrease in the MPV at Post and Post-10 compared to Pre (*p* < 0.00–0.02). However, women exhibited a decreased MPV until Post-24 (*p* = 0.02), while men had already recovered. The results show that in men and women with similar relative strength, the same training stimulus (represented by the velocity loss) generates very similar levels of immediate mechanical fatigue; however, after 24 h, men exhibit a higher rate of recovery compared to women.

**Keywords:** resistance training; fatigue; metabolic response; sex differences

**Funding:** This research received no external funding.

**References**
  1. Nuzzo, J. L. Sex differences in skeletal muscle fiber types: A meta-analysis. Clin Anat. 2024, 37(1), 81–91.
  2. Voskuil, C. C., Dudar, M. D., & Carr, J. C. Sex differences in fatiguability during single-joint resistance exercise in a resistance-46 trained population. Eur J Appl Physiol. 2024
  3. Pareja-Blanco, F., Walker, S., & Häkkinen, K. Validity of Using Velocity to Estimate Intensity in Resistance Exercises in Men and 48 Women. Int J Sports Med. 2020, 41(14), 1047–1055.

### 2.83. Comparison of Two Training Protocols with Different Velocity Losses in the Set on Bench Press Performance

**Mariscal-Campón G ^1^, Merino-Pérez J ^2^ and Pareja-Blanco F ^2^**

^1^  Faculty of Sport Sciences, Department of Sports and Computer Sciences, Universidad Pablo de Olavide; gonzalomc5423@gmail.com
^2^  Faculty of Sport Sciences, Department of Sports and Computer Sciences, Universidad Pablo de Olavide, 41013 Seville, Spain; merinojavi8@gmail.com

* Correspondence: fparbla@gmail.com; Tel.: +34-653121522

**Abstract:** In published studies on velocity-based training programs, the velocity loss (VL) in the set remains constant in all sessions [1,2]. However, no studies have compared the effects of two different models that vary in the progression of the VL threshold attained in each session. Therefore, the aim of this study was to compare two bench press training programs that differed in the progression of VL using relative intensities from 60% to 75% of 1RM. The sample was divided into the Stable Group (STA, n = 17) and the Undulating Group (UND, n = 15). STA achieved a 25% VL in all training sessions, while UND alternated between 0% and 50% VL thresholds in every training session; thus, both groups reached the same average VL (25%) at each relative intensity. The variables analyzed were 1RM, maximum unloaded velocity (V0), mean propulsive velocity (MPV) with the common absolute loads in both tests (AV), the mean MPV with the absolute loads above and below 0.8 m·s^−1^ common to the pre-test (AV > 0.8 and AV < 0.8), the maximum number of repetitions to failure (MNR), and the mean MPV of the common repetitions performed during the fatigue pre-test (AV-MNR). A “group × time” interaction was observed for AV and AV < 0.8, where STA showed greater gains. A significant time effect was found for all variables analyzed, except AV > 0.8. STA showed significant performance improvements earlier than UND. In conclusion, both models had positive effects on physical performance in the bench press. However, a model where the VL remains stable throughout all sessions has greater gains in strength, as evidenced by the significant differences in some performance variables compared to the undulating model.

**Keywords:** resistance training; velocity-based training; physical performance; programming; fatigue

**Funding:** This research received no external funding.

**References**
  1. Pareja-Blanco F, Alcazar J, Cornejo-Daza PJ, Sánchez-Valdepeñas J, Rodriguez-Lopez C, Hidalgo-de Mora J, Sánchez-Moreno M, Bachero-Mena B, Alegre LM, Ortega-Becerra M. Effects of velocity loss in the bench press exercise on strength gains, neuromuscular adaptations, and muscle hypertrophy. Scand J Med Sci Sports. 2020 Nov;30(11):2154–2166.
  2. Rodiles-Guerrero L, Cornejo-Daza PJ, Sánchez-Valdepeñas J, Alcazar J, Rodriguez-López C, Sánchez-Moreno M, Alegre LM, León-Prados JA, Pareja-Blanco F. Specific Adaptations to 0%, 15%, 25%, and 50% Velocity-Loss Thresholds During Bench Press Training. Int J Sports Physiol Perform. 2022 Jun 20;17(8):1231–1241. doi: 10.1123/ijspp.2021-0481. PMID: 35728808.

### 2.84. Differences in Countermovement Vertical Jump Force–Time Metrics Between Professional and Semi-Professional Male Basketball Players

**Carlos Suárez-Balsera ^1^, Davide Ferioli ^2^, Elena Marín-Cascales ^1,3,4^, Vincenzo Rago ^5^, Konstantinos Spyrou ^1,3,4^, Antonio Martínez-Serrano ^1,3,4^, Debora Di Mauro ^2^, Jose Manuel Marín ^6^, Pedro E. Alcaraz ^1,3^ and Tomás T. Freitas ^1,3,4,7^**

^1^  UCAM Research Center for High Performance Sport, Catholic University of Murcia, Murcia, Spain
^2^  Department of Biomedical Sciences, Dental Sciences, and Morpho-Functional Imaging, University of Messina, Messina, Italy
^3^  Facultad de Deporte, UCAM Universidad Católica de Murcia, Murcia, España
^4^  SCS—Strength & Conditioning Society, Murcia, Spain
^5^  Faculty of Health and Sports Sciences, Universidade Europeia, Lisbon, Portugal
^6^  UCAM Club de Baloncesto, Universidad Católica de Murcia, Murcia, España
^7^  NAR—Nucleus of High Performance in Sport, São Paulo, Brazil

**Abstract:** Basketball is an intermittent team sport encompassing repeated transitions between offensive and defensive phases that involve high-intensity efforts interspersed with low-intensity activities [1]. Among the wide variety of physical and motor skills that basketball players need to be proficient in to be successful, the vertical jump is conceivably one of the most important. Previous studies investigating differences in the countermovement jump (CMJ) metrics based on the level of competitive play, focusing mainly on the concentric phase of the jump and showing contrasting results; specifically, of the different metrics analyzed, the variables absolute peak force and power were the most important at discriminating between competitive playing levels [2]. To date, only two studies have investigated both eccentric and concentric phases’ specific metrics derived from CMJ in basketball to compare playing time [3] and playing positions [4]. However, no information is available about these metrics based on the competitive level. Therefore, this study aimed to compare different force–time CMJ metrics in adult male basketball players after accounting for the competitive playing level (i.e., Professional [PRO] vs. Semi-Professional [SEMI-PRO]). At the beginning of the pre-season, 39 PRO (first division; age, 26.7 [25.7;27.7] years; height, 198 [197;200] cm; body mass, 96.7 [93.9;99.6] kg) and 39 SEMI-PRO (fourth division; age, 19.5 [18.5;20.4] years; height, 197 [195;198] cm; body mass, 88.5 [85.7;91.2] kg) players completed a CMJ test on a force platform (Kistler 9286BA, Kistler Group, Winterthur, Switzerland), and a total of 21 metrics of the concentric and eccentric CMJ phases were analyzed using a specific software (ForceDecks, VALD Performance, Brisbane, Australia). Linear mixed models showed that participants in the PRO group jumped significantly higher (*p* = 0.022) than those in the SEMI-PRO group. No differences were observed in metrics related to the eccentric phase. Values of four concentric-phase metrics (i.e., absolute peak force [*p* < 0.001], absolute and relative peak powers [*p* < 0.001 and *p* = 0.037, respectively], and peak velocity [*p* = 0.036]) were superior in the PRO group compared to the SEMI-PRO group. In conclusion, jump height and four concentric-phase metrics were found to discriminate between competitive levels. Practitioners should conduct thorough CMJ analyses and could potentially consider these metrics for performance profiling and talent identification.

**Keywords:** CMJ; competitive level; force; neuromuscular; team sport

**Funding:** This research received no external funding.

**References**
  1. Petway, A.J., Freitas, T.T., Calleja-González, J., Medina Leal, D., & Alcaraz, P.E. Training load and match-play demands in basketball based on competition level: a systematic review. PLoS ONE. 2020, 15(3), e0229212.
  2. Ferioli, D., Rampinini, E., Bosio, A., La Torre, A., Azzolini, M., & Coutts, A.J. The physical profile of adult male basketball players: differences between competitive levels and playing positions. J Sports Sci. 2018, 36(22), 2567–2574.
  3. Cabarkapa, D., Cabarkapa, D.V., Aleksic, J., Philipp, N.M., Scott, A.A., Johnson, Q. R., & Fry, A. C. Differences in countermove- ment vertical jump force-time metrics between starting and non-starting professional male basketball players. Front Sports Act Living. 2023, 5, 1327379.
  4. Cabarkapa, D., Philipp, N.M., Cabarkapa, D.V., & Fry, A. C. Position-specific differences in countermovement vertical jump force-time metrics in professional male basketball players. Front Sports Act Living. 2023, 5, 1218234.

### 2.85. Concurrent Training in Older People After Valve Replacement Surgery: A Randomized Controlled Study

Ainara Gómez-Hidalgo ^1,2^, Amelia Guadalupe-Grau ^1,2,3^, Irene Rodríguez-Gómez ^1,2,3,4^, Susana Martín-Braojos ^4^, Iván Baltasar-Fernández ^1,2,3,5^, Mikel García-Aguirre ^1,2,3^, Luis M. Alegre ^1,2,3^, Ignacio Ara ^1,2,3^, Francisco José García-García ^2,3,4^ and Ana Alfaro-Acha ^2,3,4,^*

^1^  GENUD Toledo Research Group, Faculty of Sports Sciences, Universidad de Castilla-La Mancha, Toledo, Spain
^2^  Instituto de Investigación Sanitaria de Castilla-La Mancha (IDISCAM), Junta de Comunidades de Castilla-La Mancha (JCCM), Toledo, Spain
^3^  CIBER on Frailty and Healthy Ageing (CIBERFES), Instituto de Salud Carlos III, Madrid, Spain
^4^  Department of Geriatrics, Hospital Virgen del Valle, Complejo Hospitalario Universitario de Toledo, Toledo, Spain
^5^  Faculty of Health Sciences, Universidad de Castilla-La Mancha, Talavera de la Reina, Spain

* Correspondence: anaalfaroacha@yahoo.com

**Abstract:** Cardiovascular diseases are the leading cause of death worldwide, particularly among older adults (1). Mortality from heart disease decreases significantly when patients participate in a Cardiac Rehabilitation Program, which includes physical exercise as a fundamental component (2). This study aims to evaluate the effects of an 8-week combined power-oriented cluster resistance training (CRT) and fast walking interval training (IT) program on anthropometric measures, physical performance, frailty, disability, and biochemical markers in older adults one month after heart valve replacement surgery. Fifteen older adults (aged 70 to 85 years) participated in this study and were randomly divided into two groups: an intervention group (INT; n = 7), which underwent a CRT + IT training program twice weekly for 8 weeks., and a control group (CON; n = 8), which continued their usual lifestyle. Anthropometrics, diastolic (DBP) and systolic blood pressure (SBP), Short Physical Performance Battery (SPPB), Frailty Trait Scale-5 (FTS-5), I. Barthel and I. Lawton questionnaires, as well as serum fasting glucose, hematocrit, hemoglobin, triglycerides, and cholesterol levels were assessed at baseline and after 8 weeks. Linear mixed-effects models were used to assess the effects of the intervention. The INT group experienced improvements in 4 m walking speed (INT from 0.68 ± 0.14 m/s to 0.91 ± 0.14 m/s *p* < 0.05 vs. CON from 0.65 ± 0.14 m/s to 0.70 ± 0.16 m/s *p* > 0.05), sit-to-stand test (STS) (INT from 13.37 ± 2.20 s to 8.46 ± 4.49 s vs. CON from 15.23 ± 3.76 s to 12.12 ± 3.69 s; all *p* < 0.05), FTS-5 (INT from 19.71 ± 3.38 points to 15.21 ± 3.94 points *p* < 0.05 vs. CON from 22.71 ± 2.20 points to 20.86 ± 3.34 points *p* > 0.05), glucose (INT from 95.43 ± 25.44 mg/dL to 94.71 ± 9.86 mg/dL vs. CON from 113.14 ± 16.29 mg/dL to 121.29 ± 11.18 mg/dL; all *p* > 0.05), hemoglobin (INT from 11.79 ± 0.38 gr/dL to 12.81 ± 0.45 gr/dL vs. CON from 11.51 ± 0.85 gr/dL to 12.36 ± 1.11 gr/dL; all *p* < 0.05), and I. Lawton (INT from 5.00 ± 1.28 points to 6.14 ± 1.57 points vs. CON from 4.38 ± 1.19 points to 5.25 ± 1.53 points; all *p* < 0.05) test scores compared to the CON group. No significant differences were observed in anthropometrics, SBP, and DBP measurements. The concurrent training program significantly improved physical fitness, reduced frailty, enhanced disability scores, and optimized blood biochemical markers in older adults following heart valve replacement surgery.

**Keywords:** cardiovascular disease; cardiac rehabilitation program; physical exercise; training; older people

**Funding:** This research was funded by Fondo de Investigaciones Sanitarias Instituto de salud Carlos III, grant number PI18/00724, and co-financed by Fondo Europeo de Desarrollo Regional (FEDER). IRG is supported by a Sara Borrell contract from Instituto de Salud Carlos III (CD23/00236).

**References**
  1. Roth, G. A.; Mensah, G. A.; & Fuster, V. The global burden of cardiovascular diseases and risks: a compass for global action. Journal of the American College of Cardiology 2020, 76(25), 2980–2981.
  2. Balady, G. J.; Williams, M. A.; Ades, P. A.; Bittner, V.; Comoss, P.; Foody, J. A.; Franklin, B.; Sanderson, B.; Southard, D. American Heart Association Exercise, Cardiac Rehabilitation, and Prevention Committee, Council on Clinical Cardiology, Councils on Cardiovascular Nursing, Epidemiology and Prevention, and Nutrition, Physical Activity, and Metabolism, & American Association of Cardiovascular and Pulmonary Rehabilitation. Core components of cardiac rehabilitation/secondary prevention programs: 2007 update: a scientific statement from the American Heart Association Exercise, Cardiac Rehabilitation, and Prevention Committee, the Council on Clinical Cardiology; the Councils on Cardiovascular Nursing, Epidemiology and Prevention, and Nutrition, Physical Activity, and Metabolism; and the American Association of Cardiovascular and Pulmonary Rehabilitation. Journal of cardiopulmonary rehabilitation and prevention 2007, 27(3), 121–129.

### 2.86. Predicting Fracture Risk in Older People: A Retrospective Study of the Impact of Body Composition and Level of Physical Fitness

Jhojan Martínez ^1,2,3^, Ana Moradell ^1,2,3,^ Isabel Iguacel ^1,4,5,6^, David Navarrete ^1,2,4,6,^ Ángel-Iván Fernández García ^1,2^, Marcela González-Gross ^7^, Jorge Pérez-Gómez ^8^, Ignacio Ara ^9^, José Antonio Casajús ^1,2,4,5,10,^ Alba Gómez-Cabello ^1,2,11,^ Germán Vicente-Rodríguez ^1,2,4,5,12,^*

^1^  Growth, Exercise, Nutrition and Development (EXER-GENUD) Research Group, Universidad de Zaragoza, Zaragoza, Spain; amoradell@unizar.es; iguacel@unizar.es; dnavarrete@unizar.es; angelivanfg@unizar.es; joseant@unizar.es; agomez@unizar.es
^2^  Exercise and Health in Special Population Spanish Research Net (EXERNET)
^3^  Instituto de investigación Sanitaria de Aragón (IIS Aragón), 50009, Zaragoza, Spain
^4^  Instituto Agroalimentario de Aragón-IA2 (Universidad de Zaragoza-CITA), 50090, Zaragoza, Spain
^5^  Centro de Investigación Biomédica en Red de Fisiopatología de la Obesidad y Nutrición (CIBEROBN), Instituto de Salud Carlos III, 28040 Madrid, Spain
^6^  Department of Physiatry and Nursing, Faculty of Health, University of Zaragoza, 50009 Zaragoza, Spain
^7^  ImFINE Research Group, Universidad Politécnica de Madrid, 28040 Madrid, Spain; marcela.gonzalez.gross@upm.es
^8^  HEME Research Group, University of Extremadura, 10003 Cáceres, Spain jorgepg100@unex.es
^9^  GENUD-Toledo Research Group, Universidad de Castilla-La Mancha, 45071 Toledo, Spain; ignacio.ara@uclm.es
^10^  Department of Physiatry and Nursing, Faculty of Medicine, University of Zaragoza, 50009, Zaragoza, Spain
^11^  Centro Universitario de la Defensa, Zaragoza, Spain
^12^  Department of Physiatry and Nursing, Faculty of Health and Sport Sciences, University of Zaragoza, 50009, Zaragoza, Spain

* Correspondence: gervicen@unizar.es

**Abstract:** Global ageing increases the proportion of older people, resulting in economic and social challenges [1]. Loss of muscle mass and bone mineral density, together with deteriorating physical fitness, increases the risk of fractures [2,3]. This study examined whether physical fitness and body composition can predict future fracture risk in older people. The longitudinal EXERNET-Elder study assessed 3136 older people in Spain between 2008 and 2016, with complete data for 702 participants included in this analysis. Physical fitness and body composition were assessed, and fractures were recorded using ad hoc questions: “Have you suffered a fracture in the last 10 years?” For data analysis, binomial logistic regression was used to assess whether physical fitness and body composition in 2008 predicted fractures in 2016, testing a general model and one separated by sex. The general model showed that longer time on the gait speed test (*p* < 0.05), higher percentage of fat mass (*p* < 0.05), and greater leg flexibility (*p* < 0.05) were significantly correlated with increased fracture risk. The overall model has an AUC of 0.659, indicating a 65.9% ability to assign a higher probability of fracture to a fractured versus non-fractured person. The sex-specific analyses showed that in men, maximum arm strength was significant only in combination with leg strength (*p* < 0.05), showing that greater limb strength is associated with a lower risk of fracture. The model for men had the same predictive ability as the general model, with an AUC of 0.659. In women, a higher percentage of fat mass (*p* < 0.05) was associated with a higher risk of fractures; a longer time in the gait speed test (*p* < 0.05) and greater leg flexibility (*p* < 0.05) were also predictors. The model for women showed an AUC of 0.614, indicating a lower predictive ability compared to the general model and the model for men. In conclusion, considering variables specific to body composition and physical fitness can help predict fracture risk in older people. Older people with poorer physical fitness and a higher percentage of fat mass have a higher risk of fractures. Gait speed, leg flexibility, and fat mass percentage are key factors in this prediction, with notable differences between men and women. These results indicate where exercise programs should be focused during ageing to decrease fracture risk in this population.

**Keywords:** ageing; fractures; body composition; physical fitness; fat mass; walking speed; flexibility; muscle strength

**Funding:** This study was funded by “Ministerio de Economía, Industria y Competitividad” (DEP2016-78309-R), “Centro Universitario de la Defensa de Zaragoza” (UZCUD2017-BIO-01),” CIBER -Consorcio Centro de Investigación Biomédica en Red-“ (CB16/10/00477) and (CB12/03/30038), “Instituto de Salud Carlos III, Ministerio de Ciencia e Innovación and Unión Europea—European Regional Development Fund”, “Universidad de Zaragoza, Programa Propio de Investigación del Vicerrectorado de Política Científica, Proyectos Puente 2022” (UZ2021-BIO-05) and “Consejo Superior de Deportes, Ministerio de Cultura y Deporte, Plan de Recuperación Transformación y Resliencia, Next Generation European Funds” (EXP_75079).

**References**
  1. Vristea M, Noja GG, Stefea P, Sala AL. The Impact of Population Aging and Public Health Support on EU Labor Markets. International Journal Of Environmental Research And Public Health/International Journal Of Environmental Research And Public Health [Internet]. 24 de febrero de 2020;17(4):1439. Disponible en: https://doi.org/10.3390/ijerph17041439.
  2. Alajlouni DA, Bliuc D, Tran TS, Blank RD, Center JR. Muscle strength and physical performance contribute to and improve fracture risk prediction in older people: A narrative review. Bone [Internet]. 1 de julio de 2023;172:116755. Disponible en: https://doi.org/10.1016/j.bone.2023.116755
  3. Gandham A, Zengin A, Bonham MP, Winzenberg T, Balogun S, Wu F, et al. Incidence and predictors of fractures in older adults withand without obesity defined by body mass index versus body fat percentage. Bone [Internet]. 1 de noviembre de 2020;140:115546. Disponible en: https://doi.org/10.1016/j.bone.2020.115546

### 2.87. Effects of Different Types of Training on the Functional and Cognitive Capacity of Healthy Older People: Partial Results of a Randomized Clinical Trial

Nadyne Rubin ^1^, Eduarda Blanco Rambo ^2^, Caroline Rosa Muraro ^3^, Marcelo Bandeira-Guimarães ^4^, Débora Marques ^5^, Greyse Dornelles Mello ^6^, Andressa Fergutz ^7^, Eduardo Lusa Cadore ^8,^* and Caroline Pietta Dias ^9,^*

^1^  Universidade Federal do Rio Grande do Sul, nadynerubin@yahoo.com.br, eduardarambo@gmail.com caroline.rmuraro@gmail.com, bgbandeira@gmail.com, dedy.marques@hotmail.com, reysedornelles@gmail.com, andressaufrgs1@gmail.com, carolpieta@yahoo.com.br

* Correspondence: edcadore@yahoo.com.br; Tel.: +55-(51)99119365114

**Abstract:** The aging process is accompanied by biopsychosocial changes that contribute to cognitive and functional decline (1), increasing pathological processes (2) and resulting in the development of dementia. Clinical trials suggest that exercise is a neuroprotector of brain function related to memory and learning and is an efficient non-pharmacological approach for preventing cognitive impairment resulting from aging (3). Therefore, the aim of this randomized clinical trial was to compare the effects of different training programs on the functional and cognitive performance of older people. The experimental groups were divided into traditional concurrent training (TCT), concurrent training with dance (TCD), strength training (ST), and aerobic training (AT); a control group (CG) was also included. The training sessions occurred twice a week for 12 weeks, with progressive intensity and volume. All training interventions were designed to reach an equivalent exercise time throughout the training period. Dual task performance (i.e., functional and cognitive) was assessed using the Timed Up and Go Cognitive test (TUGcog). Cognitive function was also assessed by the mini-Mental State Examination (MMSE) and Subjective Memory Perception Questionnaire (MAC-Q). The Geriatric Depression Scale (GDS) was also evaluated. The main effects of time, group, and time × group interaction were analyzed using generalized estimating equations, with Bonferroni post-hoc tests for pairwise comparisons. Significance was accepted when *p* < 0.05. The analyses were conducted in SPSS software. The sample consisted of 56 participants (79% women; 68.3 ± 3.9 years old) who were randomized into the five groups mentioned. After 12 weeks of intervention, there was no time vs. group interaction for any of the assessed outcomes. However, significant time effects were observed for TUGcog (*p* < 0.001) and MMSE (*p* = 0.006). No significant main effects of interaction were observed for MAC-Q and GDS. The present findings demonstrate that all exercise interventions improved cognitive function in older adults after 12 weeks.

**Keywords:** aging; cognition; strength training; endurance training; combined training; multicomponent training; dance

**Funding:** This research was funded by the National Council for Scientific and Technological Development (CNPq) and by the Coordination for the Improvement of Higher Education Personnel (CAPES).

**References**
  1. Moorhouse P, Rockwood K. Frailty and its quantitative clinical evaluation. JRColl Physicians Edinb. 2012; 42(4):333–40.
  2. Isaev NK, Genrikhs EE, Oborina MV, Stelmashook EV. Accelerated aging and aging process in the brain. Reviews in the Neu-rosciences, v.29, n.3, 233–240, 2017.5.
  3. Bashiri H, Enayati M, Bashiri A, Salari AA. Swimming exercise improves cognitive and behavioral disorders in male NMRImice with sporadic Alzheimer-like disease. Physiology & Behavior, v. 223, p. 113003, 2020

### 2.88. Effect of Self-Myofascial Release on Plantar Fascia Compared to Foam Roller on Flexibility and Vertical Jump

Danilo Gaias ^1,^*, Francesco Masala ^1^, Maurizio Fara ^1^ and Antonio Martínez-Serrano ^2,3,4^

^1^  Himalaya Lab; himalayalab23gmail.com
^2^  UCAM Research Center for High Performance Sport, Catholic University of Murcia, Murcia, Spain; amartinez30@ucam.edu
^3^  Strength and Conditioning Society, Murcia, Spain
^4^  Faculty of Sport Sciences, Catholic University of Murcia, Murcia, Spain

* Correspondence: danilo.gaias26@gmail.com; Tel.: +34-03161106

**Abstract:** Self-myofascial release (SMFR) is a treatment performed by applying pressure on one or more muscles using a tool. The benefits of this practice are increased flexibility and enhanced recovery without detrimental effects on performance (1). While information has been provided on the effects on the flexibility and performance outcomes of this treatment using a foam roller (2), little is known about the effects of SFMR on the plantar fascia. Therefore, the aim of this study was to determine the effects of a new tool for SMFR of the plantar fascia (Auramat^®^) on posterior chain flexibility and vertical jump height and to compare those effects with the effects of a SMFR with a more common tool, the foam roller (FR). The study was a randomized, counterbalanced, crossover design, where 20 recreationally active participants (10 males, 10 females; age = 37 ± 9.39 years; body mass = 62 ± 14.00 kg; height = 1.67 ± 9.99 m) attended the laboratory four times over a 2-week period. The first session was the familiarization session to perform the straight leg raise test (SLRT), countermovement jump (CMJ), and SMFR with FR and AUR. In the next sessions, the participants who were randomly assigned to one of three intervention protocols, control (CTR), foam roller (FR), and Auramat^®^ (AUR), performed the protocols in a random succession separated by 48 h. During the testing sessions, SLRT was performed on both dominant (DOM) and non-dominant (NON-DOM) limbs and CMJ pre and post-intervention. A two-way ANOVA with repeated measures for two factors (group and limb) was performed on SLRT. No significant differences for limb (*p* = 0.51), group (*p* = 0.072), and limb*group (*p* = 0.85) were found. %change of pre and post measurements of both limbs was taken as the average of DOM and NON-DOM and used in an ANOVA with repeated measures for one factor, group, on SLRT and CMJ. No significant change was found for flexibility (*p* = 0.072) or CMJ height, although a nearly significant decrease in performance (*p* = 0.063) was observed. SMFR with AUR (SLRT: +0.007% ± 0.03; CMJ: −0.064% ± 0.069) or FR (SLRT: +0.026% ± 0.05; CMJ: −0.036% ± 0.03) had no effect on increasing flexibility or enhancing vertical jump compared to CTR (SLRT: −0.006% ± 0.043; CMJ: −0.027% ± 0.03), maybe because the testing itself served as a warm-up protocol. Despite these results, both treatments seem to show effects that lean toward an increase in flexibility and a decrease in vertical jump height.

**Keywords:** flexibility; vertical jump; self-myofascial release

**Funding:** This research received no external funding.

**References**
  1. Ferreira RM, Martins PN, Goncalves RS. Effects of Self-myofascial Release Instruments on Performance and Recovery: An Umbrella Review. Int J Exerc Sci. 2022;15(3):861–83
  2. Hendricks S, Hill H, Hollander SD, Lombard W, Parker R. Effects of foam rolling on performance and recovery: A systematic review ofthe literature to guide practitioners on the use of foam rolling. J Bodyw Mov Ther. 2020 Apr;24(2):151–174

### 2.89. Joint Torque Variability as a Marker of Neuromuscular Fatigue: A Step Towards Injury Prevention?

Fernando García-Aguilar ^1,^*, Antonio Martínez-Serrano ^2,3,4^, Régis Radaelli ^5^ and Sandro R. Freitas ^6^

^1^  Sports Research Centre, Miguel Hernández University of Elche, Elche, Spain; fernando.garciaa@umh.es
^2^  UCAM Research Center for High Performance Sport, Catholic University of Murcia, Murcia, Spain; amartinez30@ucam.edu
^3^  Strength and Conditioning Society, Murcia, Spain
^4^  Faculty of Sport Sciences, Catholic University of Murcia, Murcia, Spain
^5^  Egas Moniz School of Health & Science and Member of Egas Moniz Center for Interdisciplinary Research, Almada, Portugal; regisradaelli87@gmail.com
^6^  Neuromuscular Research Lab, Faculty of Human Kinetics, University of Lisboa, Cruz Quebrada Dafundo, Portugal; sfreitas@fmh.ulisboa.pt

* Correspondence: fernando.garciaa@umh.es; Tel.: +34-618838352

**Abstract:** Avoiding injury is a common desire of team sports athletes and clubs due to its economic and performance impacts (1). Fatigue, both acute and cumulative, is a major factor in injury development (2). Understanding the neuromuscular effects of fatigue on muscles can aid in injury prevention. The variability of force control has been suggested as a potential tool to detect fatigue, but further research is needed to validate this marker (3). This study aimed to test whether variability in knee flexor force production is a valid indicator of fatigue level. Seventeen male recreational athletes performed a unilateral knee flexor isometric contraction task (lying ventral decubitus, with knee flexed at 30°) at 40% of maximal voluntary isometric contraction (MVIC) until failure. MVIC was performed to detect fatigue effects before and after the task. The full force signal time series was divided into three segments: from 0 to 33% (start), 33 to 66% (middle), and 66 to 100% (end). Each segment was analyzed using both linear [standard deviation (SD) and coefficient of variation (CV)] and non-linear [sample entropy (SaEn) and detrended fluctuation analysis (DFA)] variability approaches. Normality tests and one-way ANOVA (time) were conducted, along with correlation analysis. The protocol resulted in a 21.48 ± 13.55% decrease in MVIC as a fatigue effect. Significant effects (*p* < 0.05) were found for all variables, indicating fatigue-induced changes in variability. Pairwise comparisons revealed significant differences in SD, CV, and SaEn across all time points, while DFA showed differences between the start and both the middle and end of the task, but not between the middle and end. Moderate to strong correlations were found between linear and non-linear variables (0.68 < r < 0.85). In accordance with previous research, variability increased in linear measures and complexity decreased in non-linear measures (decrease in SaEn and increase in DFA). All four variables discriminated between the start (non-fatigue state) and the end (fatigue state), suggesting that these variables reflect changes in the neuromuscular system (4). The strong correlations found indicate that all four variables provide insights into these neuromuscular changes. Further research is needed to understand the differences between the variables and the underlying causes of these variations. This study opens up a line of research to apply this test to athletes to determine the state of their neuromuscular systems. The results suggest that variability may be a marker for identifying fatigue status during a training or playing season.

**Keywords:** neuromuscular; force control; variability; injury; fatigue; knee flexors

**Funding:** This research received no external funding.

**References**
  1. Maniar, N.; Carmichael, D.S.; Hickey, J.T.; et al. Incidence and prevalence of hamstring injuries in field-based team sports: a systematic review and meta-analysis of 5952 injuries from over 7 million exposure hours. Br. J. Sports Med. 2023, 57, 109–116
  2. Bestwick-Stevenson T, Toone R, Neupert E, Edwards K, Kluzek S. Assessment of Fatigue and Recovery in Sport: Narrative Review. Int J Sports Med. 2021;43(14):1151–62.
  3. García-Aguilar, F.; Caballero, C.; Sabido, R.; Moreno, F.J. The use of non-linear tools to analyze the variability of force production as an index of fatigue: A systematic review. Front. Physiol. 2022, 13, 1074652.
  4. Pethick, J.; Tallent, J. The Neuromuscular Fatigue-Induced Loss of Muscle Force Control. Sports 2022, 10, 184.

### 2.90. Voluntary Use of Step-Tracker Mobile Apps by Adolescents Does Not Maintain the Benefits on Physical Activity Level and Adiposity Variables Obtained During a Period of Mandatory Use

Adrián Mateo-Orcajada ^1,^*, Raquel Vaquero-Cristóbal ^2^ and Lucía Abenza-Cano ^1^

^1^  Facultad de Deporte, UCAM Universidad Católica de Murcia, Murcia, Spain; labenza@ucam.edu
^2^  Research Group Movement Sciences and Sport (MS&SPORT), Department of Physical Activity and Sport, Faculty of Sport Sciences, University of Murcia, Murcia, Spain; raquel.vaquero@um.es

* Correspondence: amateo5@ucam.edu; Tel.: +34-968278824

**Abstract:** Mobile apps have become an effective resource for promoting physical activity in the adolescent population [1]. However, most of the interventions that use mobile apps do so by promoting their use from the school center, which has resulted in significant improvements in the level of physical activity and body composition of adolescents [2]. However, it is not known whether these improvements would be maintained when the use of these devices is voluntary. For this reason, the aim of the present investigation was to determine whether the changes achieved in the level of physical activity and adipose variables after a period of mandatory use of step tracker mobile apps were maintained during a period of voluntary use. A total of 376 adolescents between the ages of twelve and sixteen participated in the research (mean age: 13.92 ± 1.91 years). Participants were divided into a control group (CG) and an experimental group (EG), with the latter using the mobile step-tracking apps Strava, Pacer, MapMyWalk, and Pokémon-Go [3] for two ten-week periods after school hours. During the first ten-week period, the use of these devices was mandatory and promoted during the physical education subject, while in the second period, the use was voluntary. The level of physical activity was measured using the Physical Activity Questionnaire for Adolescents, and adipose variables were measured following the protocol of the International Society for Advancement of Kinanthropometry. The adolescents were measured before the period of mandatory use (T1), at the end of this period (T2), and at the end of the second period of voluntary use (T3). The results showed that the level of physical activity in the EG increased between T1 and T2 (*p* < 0.001), but decreased significantly between T2 and T3 (*p* = 0.030). There were no significant differences at any of the time points in the CG. Regarding adipose variables, there was a significant decrease in the sum of 3 skinfolds in the EG between T1 and T2 (*p* = 0.020), and although this variable increased again between T2 and T3, it was not significant (*p* = 0.320). There were no significant differences in this variable in the CG (*p* = 0.660–1.000); additionally, there were no significant differences in fat mass in the EG (*p* = 0.070–1.000) and in the CG (*p* = 0.840–1.000). Therefore, based on the present study, it can be concluded that although there are improvements in the level of physical activity and adipose variables in the adolescents during the period of mandatory use of the mobile apps, these are not maintained when the use of the apps is voluntary.

**Keywords:** adolescents; body composition; mandatory; mobile apps; physical activity level; voluntary

**Funding:** This research received no external funding.

**References**
  1. Schoeppe, S.; Alley, S.; Rebar, A.; Hayman, M.; Bray, N.; Van Lippevelde, W.; Gnam, J.; Bachert, P.; Direito, A.; Vandelanotte, C. Apps to improve diet, physical activity and sedentary behaviour in children and adolescents: a review of quality, features and behaviour change techniques. Int J Behav Nutr Phys Act 2017, 14, 1–10.
  2. Mateo-Orcajada, A.; Abenza-Cano L.; Albaladejo-Saura, M.; Vaquero-Cristóbal, R. Mandatory after-school use of step tracker apps improves physical activity, body composition and fitness of adolescents. Educ Inf Technol 2023, 28, 10235–10266.
  3. Bondaronek, P.; Alkhaldi, G.; Slee, A.; Hamilton, F.; Murray, E. Quality of Publicly Available Physical Activity Apps: Review and Content Analysis. JMIR Mhealth Uhealth 2018, 6, e53.

